# How does the local area deprivation influence life chances for children in poverty in Wales: A record linkage cohort study

**DOI:** 10.1016/j.ssmph.2023.101370

**Published:** 2023-02-23

**Authors:** Amrita Bandyopadhyay, Tony Whiffen, Richard Fry, Sinead Brophy

**Affiliations:** a National Centre for Population Health and Wellbeing Research, Swansea University Medical School, Wales, SA2 8PP, UK; b Administrative Data Research Unit, Swansea University, Wales, SA2 8PP, UK; c Administrative Data Research Unit, Welsh Government, Wales, CF10CF10 3NQ, UK; d Health Data Research UK, Swansea University Medical School, Wales, SA2 8PP, UK

**Keywords:** Local area, Deprivation, Child poverty, Resilience, Education, Record linkage, Cohort study

## Abstract

**Objectives:**

Children growing up in poverty are less likely to achieve in school and more likely to experience mental health problems. This study examined factors in the local area that can help a child overcome the negative impact of poverty.

**Design:**

A longitudinal record linkage retrospective cohort study.

**Participants:**

This study included 159,131 children who lived in Wales and completed their age 16 exams (Key Stage 4 (KS4)) between 2009 and 2016. Free School Meal (FSM) provision was used as an indicator of household-level deprivation. Area-level deprivation was measured using the Welsh Index of Multiple Deprivation (WIMD) 2011. An encrypted unique Anonymous Linking Field was used to link the children with their health- and educational records.

**Outcome measures:**

The outcome variable ‘Profile to Leave Poverty’ (PLP) was constructed based on successful completion of age 16 exams, no mental health condition, no substance and alcohol misuse records in routine data. Logistic regression with stepwise model selection was used to investigate the association between local area deprivation and the outcome variable.

**Results:**

22% of children on FSM achieved PLP compared to 54.9% of non-FSM children. FSM Children from least deprived areas were significantly more likely to achieve PLP (adjusted odds ratio (aOR) - 2.20 (1.93, 2.51)) than FSM children from most deprived areas. FSM children, living in areas with higher community safety, higher relative income, higher access to services, were more likely to achieve PLP than their peers.

**Conclusion:**

The findings indicate that community-level improvements such as increasing safety, connectivity and employment might help in child's education attainment, mental health and reduce risk taking behaviours.

## Highlights

• This novel study indicates the positive effect of community development measures to build resilience of children in poverty.
• Anonymously linked, routine health and education data are used to build a nationally representative cohort of children.
• Child’s resilience profile is developed based on child’s educational attainment, mental health, risk-taking behaviours.

## 1 Introduction

Latest figures suggest that in 2020, 29.3% of children aged between 0 and 19 are living in poverty (i.e. family income below 60% of the median income) in Wales, which is a 1% rise compare to the previous year (Observatory, n.d.). Living in persistent poverty has a detrimental impact on child health, cognitive and behavioural outcomes (Wickham et al., 2016). Child poverty has caused an unprecedented increase in infant mortality in recent years in the UK (Taylor-Robinson et al., 2019). After a steady fall in the last decade (post-2010), the child poverty rate has also now started to increase in the UK (Joyce, 2014; Taylor-Robinson et al., 2019). In the post-recession recovery period (i.e. since 2008) inequality increased because of disproportionately slow recovery for low-income families (Beatty & Fothergill, 2016; Cribb et al., 2018). This is due to real-term cuts in benefits, increasing housing costs and restricted possibilities to improve income from work (e.g. due to salary reductions, freeze in promotions) (Lambie‐Mumford & Green, 2017). As a result, of all children living in relative poverty, the majority are from working families as opposed to workless households (Vizard et al., 2019). Currently 67% of the children in relative poverty are living in households where at least one person is working (Welsh Government, 2019b). A report from *End Child Poverty* carried out by Loughborough University has shown that child poverty is disproportionately rising in the UK's most impoverished areas (Loughborough, 2019). The report shows that in some parts of Wales, children from deprived families are six times more likely to grow up in poverty than their neighbours if they are living in less deprived areas. The latest report from the Welsh Index of Multiple Deprivation (WIMD) 2019 from Welsh Government highlighted ‘deep-rooted’ deprivation by highlighting the areas in Wales which have remained as the top most deprived areas for more than last 15 years, which indicates a lack of social mobility in most of these areas (Welsh Government, 2019a).

A child growing up in a deprived area implies that they are more likely to be provided with insufficient educational support, lack of recreational space (no safe park or playground) and receive poor quality childcare and health support (Galster et al., 2007). This has numerous inevitable long-term consequences such as poorer mental and physical health, lower school achievement, and worse outcomes in adulthood (Featherstone et al., 2019; Galster et al., 2007; Wickham et al., 2016; Wood, 2003). Another study has found that the children in deprived area are at higher risk of early alcohol use (Bandyopadhyay et al., 2022) and early onset of alcohol use increases the risk of alcohol dependence and other illicit drug use in later life (Hingson et al., 2006). Children living in deprived neighbourhoods are less likely to complete high school and achieve higher educational attainment. This creates a significant difference in their earning levels in later life compared to their peers (Galster et al., 2007). Local areas with community safety issues often restrict children from after-school outdoor activities and increases their sedentary behaviours. This significantly contributes to childhood obesity amongst children living in poor neighbourhoods (Cecil-Karb & Grogan-Kaylor, 2009). Family- and area level disadvantageous socio-economic conditions often lead to teenage pregnancy (Penman-Aguilar et al., 2013), which is significantly associated with adverse health outcomes and social consequences (Cook & Cameron, 2017).

Although growing up in a deprived family and local area increase the risk of adverse consequences in their life, some children are more able to beat the odds than their peers despite coming from disadvantage, and show resilience (Sattler & Gershoff, 2019). Studies have investigated various factors that can be linked with overcoming odds, such as moving to a more affluent area in early childhood (Chetty et al., 2016), living in an area with better access to green space (Flouri et al., 2014), safer community areas so that parents allow and encourage their children to be involved in outdoor physical activity (Veitch et al., 2013), and neighbourhood safety that enhances collective socialisation (Marco & Vernon-Feagans, 2013; Minh et al., 2017). Though such evidence is fragmented, it indicates that improvement of the quality, facility and environment of the local area can help the children to build resilience and overcome adversity. Hence it is necessary to develop a holistic understanding of neighbourhoods and prioritise the various aspects of a local area which can help children and their parents to improve their life and overcome poverty.

Family level deprivation as a key indicator of child's poor development has been discussed in literature, but this study focuses on whether a local area level improvement can moderate this relationship. This study investigates the socio-economic determinants of a local area that are associated with the resilience in children using a linked routine data framework. The aim of the study is to develop a holistic understanding of various aspects of a local area which contribute to the resilience of the children and can help children to improve their life. This study has used the deprivation index WIMD 2011 to identify concentrations and variations of several domains of deprivation for small areas in Wales and its impact on children (n = 159,131). This work has developed a profile of children showing resilience despite family level deprivation based on factors which have significant association with improving their lives and overcoming poverty. This has been modelled as ‘Profile to Leave Poverty’ (PLP) and it has been derived based on four major components (education, mental health, alcohol, and substance misuse). The findings of the study can provide important insights for targeted policy development and intervention.

## 2 Method

### 2.1 Sample

**Fig. 1:**
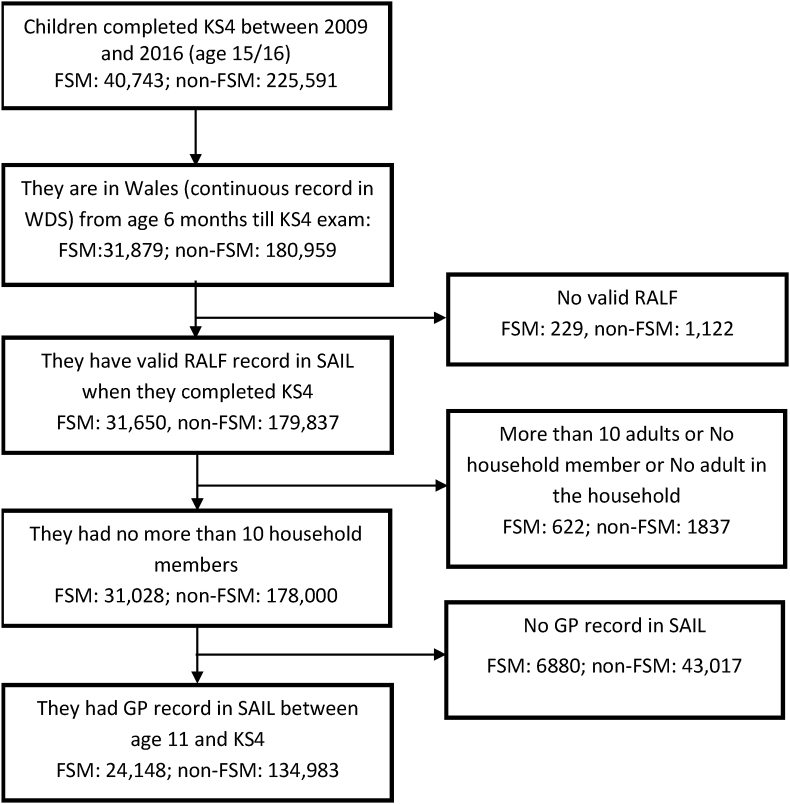
Participants consort diagram (based on Free School Meal eligibility).

The study population was comprised of children who completed their age 16 exams (Key Stage 4 (KS4)), between 2009 and 2016 and had a valid Free School Meal (FSM) record (eligible or not eligible). The selected children were either born or resident of Wales until they completed KS4. The participants were derived by linking Wales Demographic Service Dataset (WDSD) (a Wales-wide administrative register for all individuals with a general practitioner (GP)) and education datasets. Data linkage was performed with the help of an anonymised encrypted linkage key known as Anonymous Linking Field (ALF) provided by trusted third party in the Secure Anonymous Information Linkage (SAIL) databank platform at Swansea University (Ford et al., 2009; Lyons et al., 2009). To enable individuals living in the same household to be anonymously linked, Residential Anonymous Linking Field (RALFs) were created by encrypting individual's address identifiers for the study period (Johnson et al., 2021). The children who did not have a continuous residential record (valid RALF) in WDSD between age six months and KS4 exam (to ensure they lived in Wales throughout the childhood, and we had valid measures of exposures) and primary care record in Welsh Longitudinal General Practice (WLGP) dataset between age 11 and KS4 (when the outcome variable was observed) were excluded from the analysis to ensure the complete data coverage and follow-up period. A detailed participants flow diagram of the study population is provided in Fig. 1.

### 2.2 Exposure variables

In this study local area deprivation was measured by using WIMD 2011 (Welsh Government, 2011) which is the official measure of relative deprivation for small areas in Wales. Lower layer Super Output Areas (LSOA) are the geographic units used to define small areas in Wales and England. There were 1896 LSOAs in Wales and WIMD 2011 ranked all LSOAs (1 most deprived to 1896 least deprived). The study used WIMD 2011 as this was timely with the study period. In this study, individual's residential identifier RALF was linked to LSOAs, which are linked with WIMD rank aggregated into a quintile scale where a lower value denotes greater deprivation. Considering the statistical significance of the categories with respect to the study population and the interpretability of the findings, the study considered WIMD aggregated into a quintile scale instead of by decile. Along with overall WIMD rank, component scores for WIMD domains such as income, community safety, health, access to services, physical environment, housing, but not education have been considered as main exposure variables. For individuals, household level deprivation has been measured by FSM eligibility at KS4 (Taylor, 2018).

### 2.3 Covariates

The other covariates that were included in the study are - living in urban or rural area, number of adults and number of children in the household, living with someone who had depression (diagnosis and/or medication), any household member diagnosed with serious mental illness such as schizophrenia, bipolar disorder (for ICD10 and Read codes see Supplementary material Codes 1), household member who had an alcohol related hospitalisation record (for ICD10 codes see Supplementary material Codes 2) and whether the child needs special education support. Since the study builds a cohort of children who are completing KS4 between 2009 and 2016, hence to adjust the effect of different academic years, their KS4 assessment year (Exam Year) has been considered in the analysis.

### 2.4 Outcome variable

The study aimed to build a profile that can contribute to the resilience of the children. ‘Profile to Leave Poverty’ (PLP) is an indicator of overcoming poverty at the transition between adolescent and early adulthood. The resilience profile of the children known (PLP) has been developed with the four major components such as: a) poor educational attainment, b) developing mental health condition, c) early alcohol use, and d) early substance misuse. The existing literature has already shown the significant association between poverty and these four major components. It has been identified that the children living poverty are more likely to be affected by these four risk factors which will have several detrimental impacts on their later life (this has been discussed in the introduction section). Hence, the study developed a resilience profile by adding all four components where there are positive outcome from all four factors. The PLP has been derived based on the following four criterions –

a. Achieved KS4: If they have successfully completed L2EWM (level 2 English/Welsh Maths– A* to C in 5 GCSE subjects including Maths and English/Welsh)
b. No mental health condition: They have no records of the following conditions - Attention Deficit Hyperactive Disorder, Conduct Disorder, Depression, Serious Mental Illness, Self-harm between age 11 and KS4 assessment
c. No substance misuse: They have no substance misuse record between age 11 and KS4 assessment
d. No alcohol abuse: They have no alcohol related records between age 11 and KS4 assessment

The children who satisfied all four above-mentioned conditions were considered as ‘achieved’ PLP. Those who did not satisfy one of the conditions were considered as ‘not achieved’ PLP, i.e.

PLP ‘achieved’ = KS4 achieved *AND* No mental health condition record *AND* No substance misuse record *AND* No alcohol abuse record

PLP ‘not achieved’ = KS4 not achieved *OR* mental health condition record *OR* substance misuse record *OR* alcohol abuse record

The study population has been linked with relevant education data to obtain the KS4 record. Mental health, substance misuse and alcohol records were derived from hospital admissions dataset known as Patient Episode database in Wales (PEDW), primary care dataset known Welsh Longitudinal General Practice (WLGP) and substance misuse dataset. ICD-10 codes used in PEDW indicate hospital admission due to mental health conditions, substance misuse and alcohol whilst GP-recorded Read codes highlight diagnosis and medication associated with mental health conditions, substance misuse and alcohol in primary care health system. ICD-10 and Read codes are mentioned the Supplementary material Codes 3, 4 & 5.

### 2.5 Statistical analysis

The study primarily aimed to investigate the association between the resilience profile PLP derived by the study and the local area deprivation measured by WIMD among the children living in high household-level deprivation (FSM children). The current study, however, also investigated a similar association among the non-FSM children group, hence FSM-stratified analysis was performed. A supplementary analysis has discussed the interaction between FSM eligibility and WIMD. This study examined if a child's potential to leave poverty can be moderated by improvements in their in local built environment. This is measured by examining the association of the domains of WIMD (e.g. income, community safety, health, access to services, physical environment, housing) on a child's outcome in order to develop insight into the factors that best influence the child's trajectory. Logistic regression models were used to determine the association between local area deprivation measured by WIMD and achieving PLP amongst the children in Wales. The logistic regressor was augmented with stepwise bidirectional (forward and backward) search for optimal model selection (Burnham & Anderson, 2003). This method determines the best model with the minimum Akaike Information Criterion (AIC) and least significant features are excluded at each iteration step. The study has confirmed that there is no major concern around the high degree of correlation between predictor variables in the regression models by multicollinearity test (see Supplementary material collinearity test). Along with the explanatory variables, the stepwise logistic regression models have been adjusted for other covariates – such as exam year, gender, urban/rural classification of the living area, number of adults in the household, number of children in the household, living with someone who had an alcohol problem, living with someone who had depression, living with someone who had serious mental illness, child's special education need requirement – as these factors are also associated with the outcome variable. The odds ratio calculated with this adjustment has been reported throughout this work. The statistical significance of the explanatory variables and covariates have been interpreted by the p value less than 0.05. The data preparation including extraction, cleaning and linkage was performed in Structured Query Language (SQL) on an IBM DB2 platform and analyses were performed in the R statistical language version 3.3.2 (R Core Team, 2018).

## 2.6Ethical approval

This study was approved by the SAIL Databank independent Information Governance Review Panel (IGRP) (project number 0916 – WECC Phase 4).

## 3 Results

Characteristics of the study population by family level poverty as assessed using FSM are presented in Table 1. Those receiving FSM were more likely (compared to non-FSM) to live in a single parent household (29.2% compared to 13.1%, respectively), live with 3 or more other children (18.5% compared to 5.8%, respectively) in the same household or to have special educational needs (36.5% compared to 17.6%). They were also more likely to live with a household member who had an alcohol problem (11% compared to 3.8%), depression (63.3% compared to 39.8%), or a serious mental illness (4.6% compared to 1.2%).

**Table 1:** Characteristics of study population by FSM eligibility.

	FSM		Non-FSM		Difference (95%CI)
	N = 24,148	%	N = 134,983	**%**	
Gender					
Boy	12,175	50.4	68,704	50.9	
Girl	11,973	49.6	66,279	49.1	0.5(-0.2, 1.2)
Living area					
Urban	18,829	78.0	92,749	68.7	9.3(8.7, 9.8)
Rural	5319	22.0	42,234	31.3	
Number of adults in the household					
1	7062	29.2	17,639	13.1	16.2(15.6, 16.8)
2	9058	37.5	63,682	47.2	−9.7 (−10.3, −9.0)
3 and above	8028	33.2	53,662	39.8	−6.5 (−7.2, −5.9)
Number of other children in the household					
0	7079	29.3	57,438	42.6	−13.2 (−13.9, −12.6)
1	7557	31.3	50,774	37.6	−6.3 (−7.0, −5.7)
2	5036	20.9	18,878	14.0	6.9 (6.3, 7.4)
3 and above	4476	18.5	7893	5.8	12.7 (12.2, 13.2)
Living with someone who had alcohol problem					
No	21,499	89.0	129,874	96.2	
Yes	2649	11.0	5109	3.8	7.2 (6.8, 7.6)
Living with someone who had depression					
No	8865	36.7	81255	60.2	
Yes	15283	63.3	53728	39.8	23.5(22.8, 24.1)
Living with someone who had serious mental illness					
No	23,035	95.4	133,371	98.8	
Yes	1113	4.6	1612	1.2	3.4 (3.2, 3.7)
Exam year					
2009	2804	11.6	17,661	13.1	−1.5(-1.9, −1)
2010	2943	12.2	17,686	13.1	−0.9, (−1.4, −0.5)
2011	3125	12.9	17,247	12.8	.2(-0.3, 0.6)
2012	3039	12.6	16,779	12.4	.2(-0.3, 0.6)
2013	3428	14.2	17,511	13.0	1.2(0.8, 1.7)
2014	3110	12.9	16,836	12.5	0.4(-0.1, 0.9)
2015	2938	12.2	15,958	11.8	0.3(-0.1, 0.8)
2016	2761	11.4	15,305	11.3	0.1(-0.3, 0.5)
Special Education Need					
No	15,338	63.5	111,206	82.4	
Yes	8810	36.5	23,777	17.6	18.9 (18.3, 19.5)

Overall Welsh Index of Multiple Deprivation (WIMD)					
1 (Most deprived)	11,395	47.2	26,004	19.3	27.9(27.3, 28.6)
2	5891	24.4	26,724	19.8	4.6(4, 5.2)
3	3678	15.2	27,481	20.4	−5.1(-5.6, −4.6)
4	1865	7.7	24,686	18.3	−10.6(-11, −10.2)
5 (Least deprived)	1319	5.5	30,088	22.3	−16.8(17.2, −16.5)
Income WIMD					
1 (Most deprived)	11,439	47.4	25,539	18.9	28.5(27.8, 29.1)
2	6071	25.1	27,613	20.5	4.7(4.1, 5.3)
3	3599	14.9	27,105	20.1	−5.2(-5.7, −4.7)
4	2018	8.4	26,627	19.7	−11.4(-11.8, −11)
5 (Least deprived)	1021	4.2	28,099	20.8	−16.6(-16.9, −16.3)
Health WIMD					
1 (Most deprived)	10,173	42.1	26,465	19.6	22.5(21.9, 23.2)
2	6359	26.3	27,836	20.6	5.7 (5.1, 6.3)
3	3963	16.4	27,315	20.2	−3.8(-4.3, −3.3)
4	2281	9.4	25,830	19.1	−9.7(-10.1, −9.3)
5 (Least deprived)	1372	5.7	27,537	20.4	−14.7(-15.1, −14.4)
Access to service WIMD					
1 (Most deprived)	1834	7.6	23,492	17.4	−9.8(-10.2, −9.4)
2	3926	16.3	30,234	22.4	−6.1(-6.7, −5.6)
3	6206	25.7	28,194	20.9	4.8(4.2, 5.4)
4	6640	27.5	28,758	21.3	6.2(5.6, 6.8)
5 (Least deprived)	5542	23.0	24,305	18.0	4.9(4.4, 5.5)
Community safety WIMD					
1 (Most deprived)	9828	40.7	24,835	18.4	22.3(21.7, 23.0)
2	6293	26.1	27,291	20.2	5.8(5.3, 6.4)
3	4386	18.2	27,722	20.5	−2.4(-2.9, −1.8)
4	2429	10.1	28,324	21.0	−10.9(-11.4, −10.5)
5 (Least deprived)	1212	5.0	26,811	19.9	−14.8(15.2, 14.5)
Physical environment WIMD					
1 (Most deprived)	5501	22.8	26,282	19.5	3.3(2.7, 3.9)
2	4786	19.8	28,204	20.9	−1.1(-1.6, −0.5)
3	4866	20.2	28,320	21.0	−0.8(-1.4, −0.3)
4	4256	17.6	25,648	19.0	−1.4(-1.9, −0.9)
5 (Least deprived)	4739	19.6	26,529	19.7	0.0(-0.6, 0.5)
Housing WIMD					
1 (Most deprived)	6185	25.6	22,805	16.9	8.7(8.1, 9.3)
2	5422	22.5	25,205	18.7	3.8(3.2, 4.4)
3	5338	22.1	26,437	19.6	2.5(2, 3.1)
4	4756	19.7	27,487	20.4	−0.7(-1.2, −0.1)
5 (Least deprived)	2447	10.1	33,049	24.5	−14.4(-14.8, −13.9)

### 3.1 Outcomes for children on FSM

**Fig. 2:**
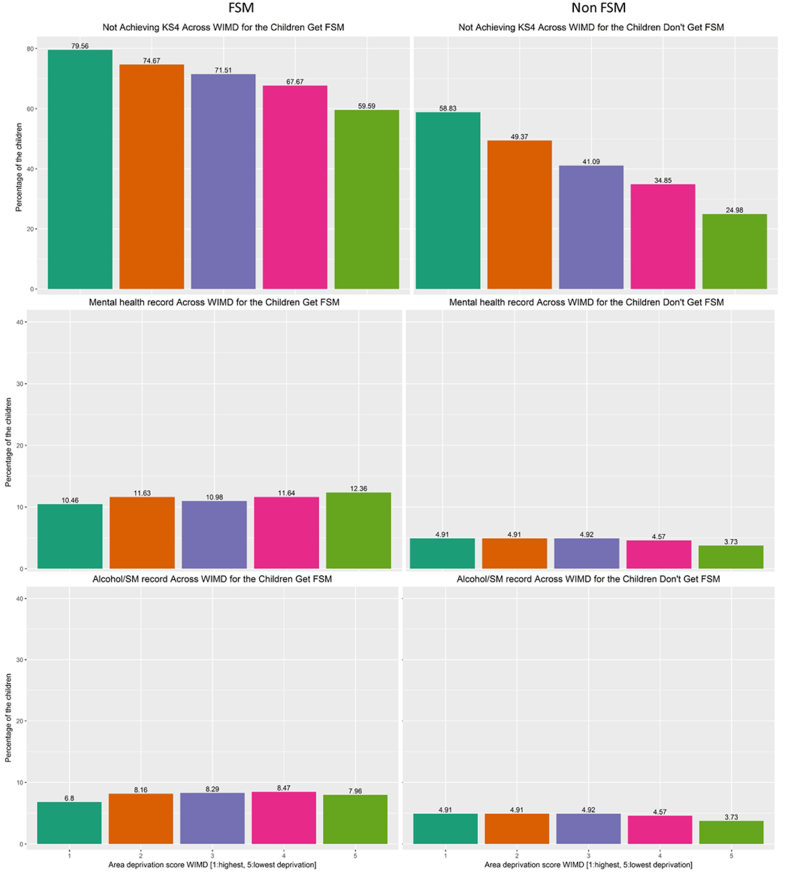
The percentage of the children (FSM and non-FSM) for each component of the outcome variable PLP across all WIMDs.

There were 22% FSM children who achieved PLP compared to 54.9% of non-FSM children (difference: 32.9% (95%CI: 32.3%, 33.5%)). Where children who did not achieve PLP this was mainly due to them not achieving KS4 (75.1% of children on FSM) and due to having a mental health condition (11% of FSM children) (see Table 2). The distribution of children for each component of the PLP across all WIMDs has been presented in Fig. 2.

**Table 2:** Breakdown of achieving PLP outcome variable.

	FSM		Non-FSM		Difference (95%CI)
	N = 24,148	%	N = 134,983	%	
PLP					
achieved	5311	22.0	74060	54.9	−32.9 (−33.5, −32.3)
not achieved	18837	78.0	60923	45.1	

KS4 not achieved:					
Achieved	6005	24.9	79083	58.6	−33.7 (−33.1,– 34.3)
Not achieved	18143	75.1	55900	41.4	
Alcohol record					
No	22645	93.8	129441	95.9	−2.1 (−2.5, −1.8)
yes	1503	6.2	5542	4.1	
Substance misuse record					
No	23709	98.2	134130	99.4	−1.2 (−1.4, −1.0)
yes	439	1.8	853	0.6	
Any mental health condition					
No	21487	89.0	128172	95.0	−6.0 (−6.4, −5.6)
yes	2661	11.0	6811	5.0

### 3.2 Factors associated with achieving PLP for children who are on Free School Meals

Children who lived in a deprived household (based on FSM eligibility) but in the least deprived areas were significantly more likely to achieve PLP (adjusted odds ratio (aOR) 2.20 (1.93, 2.51)) compared to FSM children from the most deprived areas. Living in a household containing less than 3 children, and not living with someone with an alcohol problem or depression were also associated with achieving PLP for children living in high individual-level socio-economic deprivation (see Table 3). For FSM children gender, number of adult household members and living with someone who had serious mental illness were not as significantly associated with the outcome variable, as a result bidirectional model removed them in the iteration steps.

**Table 3:** Logistic regression model of the association between overall WIMD and achieving PLP for the FSM and non-FSM children.

Variables	FSM children			non - FSM children		
	OR	Lower CI	Upper CI	OR	Lower CI	Upper CI
Overall WIMD						
1(Most deprived)	1.00			1.00		
2	1.27	1.17	1.38	1.40	1.35	1.45
3	1.46	1.32	1.60	1.88	1.81	1.95
4	1.76	1.56	1.98	2.34	2.25	2.44
5 (Least deprived)	2.20	1.93	2.51	3.47	3.34	3.61
Exam year						
2009	1.00			1.00		
2010	1.14	0.99	1.32	1.17	1.12	1.22
2011	1.29	1.12	1.49	1.23	1.18	1.29
2012	1.44	1.26	1.66	1.28	1.22	1.34
2013	1.67	1.46	1.91	1.44	1.37	1.50
2014	1.93	1.69	2.22	1.67	1.60	1.75
2015	2.31	2.01	2.65	1.93	1.84	2.03
2016	2.86	2.50	3.28	2.21	2.10	2.32
Gender						
Boys	–			1.00		
Girls	–			1.06	1.03	1.08
Living area						
Urban	1.00			1.00		
Rural	0.94	0.87	1.02	1.04	1.02	1.07
Number of adults in the household						
1	–			1.00		
2	–			1.48	1.43	1.53
3 and above	–			1.28	1.23	1.33
Number of children in the household						
0	1.00			1.00		
1	0.99	0.91	1.08	1.09	1.06	1.12
2	0.95	0.86	1.04	0.94	0.90	0.97
3 and above	0.88	0.80	0.98	0.79	0.75	0.83
Living with someone who had alcohol problem						
No	1.00			1.00		
Yes	0.77	0.68	0.86	0.62	0.58	0.66
Living with someone who had depression						
No	1.00			1.00		
Yes	0.88	0.82	0.94	0.69	0.67	0.71
Living with someone who had serious mental illness						
No	–			1.00		
Yes	–			0.89	0.80	1.00
Special Education Need						
No	1.00			1.00		
Yes	0.12	0.11	0.13	0.14	0.14	0.15

*Intercept for FSM model: 0.27(0.23, 0.30) and non-FSM model: 0.54(0.51, 0.57).

Supplementary work was conducted to investigate the association between WIMD and PLP components individually. It shows that poor children in least deprived areas are doing significantly better in education (aOR for achieving KS4 is 2.53 (2.23, 2.88)) than those living in most deprived areas. However, the trend is not similar for mental health, substance misuse and alcohol problems (see Supplementary Material Table 1).

### 3.3 Factors associated with achieving PLP for children who are not on Free School Meals

Like FSM children, non-FSM children who were living in the least deprived areas were also significantly more likely to achieve PLP (aOR 3.47 (3.34, 3.61)) compared to children living in deprived areas. Non-FSM girls were doing better than boys. The other most statistically significant factors that support these children to achieve were - not living in a single adult household, living with another child in the household and not living with someone with alcohol and mental health conditions.

The supplementary work (Supplementary Material Table 1) showed that non-FSM children living in least deprived areas were doing significantly better in all components of PLP than their peers from the most deprived areas, aOR for achieving KS4 is 3.91 (3.76, 4.06), aOR for not having mental health problems is 1.30 (1.21, 1.41), aOR for having substance misuse and alcohol problems is 1.21 (1.12, 1.32).

### 3.4 The impact of different aspects of area on achieving PLP for children on FSM

**Fig. 3:**
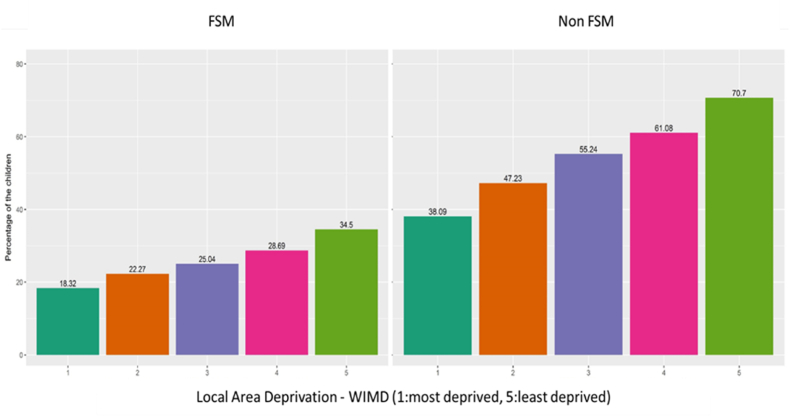
The percentage of the children (FSM and non-FSM) who are achieving PLP across all area level deprivation scores.

**Fig. 4:**
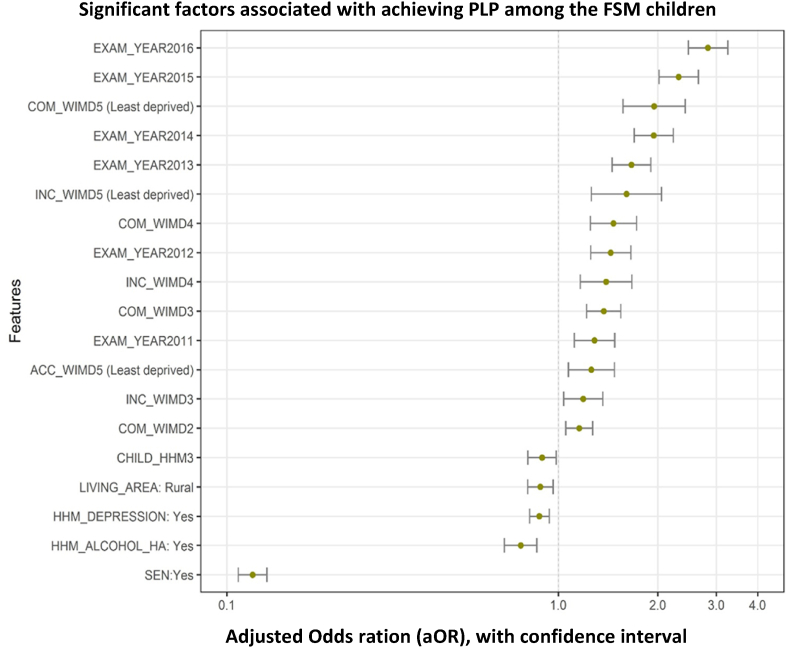
Significant factors associated with achieving PLP among the FSM children. Note: EXAM_YAER = Exam year (between 2009 and 2016), COM_WIMD = Community safety WIMD, INC_WIMD = Income WIMD, ACC_WIMD = Access to service WIMD, CHILD_HHM = Number of children in the household, LIVING_AREA = Living area, HHM_DEPRESSION = Living with someone who had depression, HHM_ALCOHOL = Living with someone who had depression, SEN = Special Education Need.

**Fig. 5:**
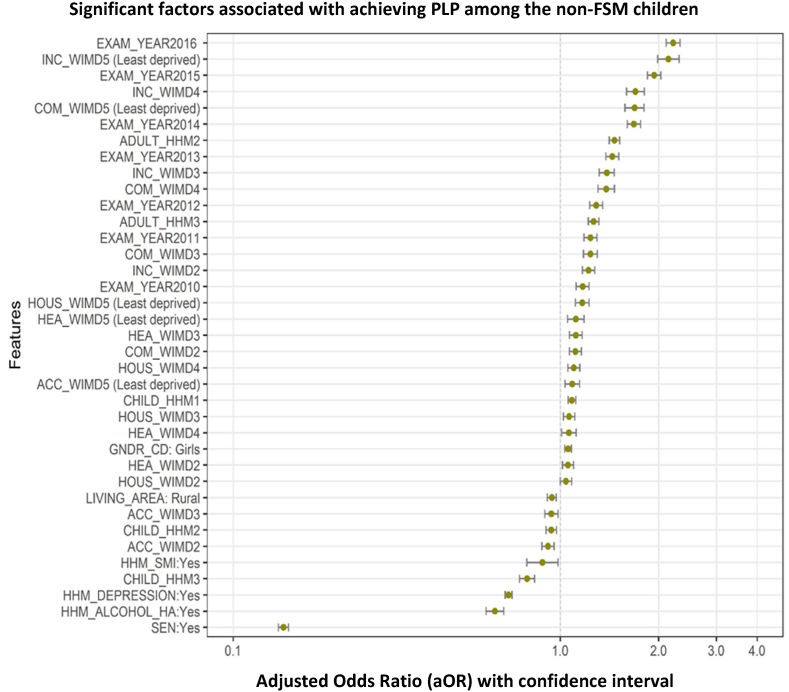
Significant factors associated with achieving PLP among the non-FSM children. Note: EXAM_YAER = Exam year (between 2009 and 2016), INC_WIMD = Income WIMD, COM_WIMD = Community safety WIMD, ADULT_HHM = Number of adults in the household, HOUS_WIMD = Housing WIMD, HEA_WIMD = Health WIMD, ACC_WIMD = Access to service WIMD, CHILD_HHM = Number of children in the household, GNDR_CD = Gender, LIVING_AREA = Living area, HHM_SMI = Living with someone who had serious mental illness, HHM_DEPRESSION = Living with someone who had depression, HHM_ALCOHOL = Living with someone who had depression, SEN = Special Education Need.

Children who were on FSM and living in deprived areas were significantly less likely to achieve PLP than children who were on FSM but living in less deprived areas (18.32% compared to 34.54%) (see Fig. 3). This figure suggests that despite household-level deprivation, children are able to achieve PLP if they are living in more affluent areas. The area components that made the most difference to children's achievement were higher community safety (1.95 times more likely to achieve for FSM children living in the safest areas compared to the least safe areas), higher relative income in the area (e.g. fewer people on benefits and more people in work, 1.61 times more likely to achieve if living in the highest income area compared to the lowest), and relatively higher access to services (1.26 times more likely to achieve if living in areas with high access to services compared to those with low access to services). After adjusting for WIMD domains children from urban areas were more likely to achieve compared to children from rural areas (see Table 4). Area characteristics that did not impact on achieving PLP included general health of people in the area or physical environment (e.g., pollution levels). Fig. 4, Fig. 5 graphically depicts the significant indicators that were associated with achieving PLP for both FSM and non-FSM children.

**Table 4:** Logistic regression model of the association between WIMD components and achieving PLP for the FSM and non-FSM children.

Variables	FSM children			non-FSM children		
	OR	Lower CI	Upper CI	OR	Lower CI	Upper CI
Income WIMD						
1(Most deprived)	1.00			1.00		
2	1.00	0.91	1.11	1.22	1.17	1.27
3	1.19	1.04	1.36	1.39	1.32	1.46
4	1.39	1.16	1.67	1.70	1.60	1.81
5 (Least deprived)	1.61	1.26	2.05	2.14	1.99	2.31
Health WIMD						
1(Most deprived)	1.00			1.00		
2	1.02	0.94	1.12	1.06	1.02	1.10
3	1.11	0.99	1.25	1.12	1.07	1.17
4	0.96	0.82	1.12	1.06	1.01	1.12
5 (Least deprived)	0.89	0.73	1.09	1.12	1.05	1.18
Access to service WIMD						
1 (Most deprived)	1.00			1.00		
2	0.97	0.83	1.13	0.92	0.88	0.96
3	1.05	0.90	1.22	0.94	0.90	0.98
4	1.03	0.88	1.20	0.97	0.92	1.01
5 (Least deprived)	1.26	1.07	1.48	1.09	1.03	1.15
Community safety WIMD						
1 (Most deprived)	1.00			1.00		
2	1.16	1.05	1.27	1.11	1.07	1.16
3	1.37	1.22	1.54	1.24	1.18	1.30
4	1.47	1.25	1.72	1.38	1.30	1.46
5 (Least deprived)	1.95	1.57	2.42	1.69	1.58	1.81
Physical environment WIMD						
1 (Most deprived)	–			1.00		
2	–			1.02	0.98	1.06
3	–			0.97	0.93	1.00
4	–			0.98	0.94	1.02
5 (Least deprived)	–			0.98	0.95	1.02
Housing WIMD						
1 (Most deprived)	–			1.00		
2	–			1.04	1.00	1.08
3	–			1.06	1.02	1.11
4	–			1.10	1.06	1.15
5 (Least deprived)	–			1.17	1.11	1.23
Exam Year						
2009	1.00			1.00		
2010	1.13	0.98	1.31	1.17	1.12	1.23
2011	1.29	1.12	1.48	1.24	1.18	1.30
2012	1.44	1.25	1.65	1.29	1.23	1.35
2013	1.66	1.45	1.90	1.44	1.38	1.51
2014	1.94	1.69	2.23	1.68	1.60	1.76
2015	2.31	2.01	2.65	1.94	1.85	2.03
2016	2.83	2.47	3.25	2.21	2.11	2.32
Gender						
Boys	–			1.00		
Girls	–			1.06	1.03	1.08
Living area						
Urban	1.00			1.00		
Rural	0.88	0.81	0.96	0.94	0.91	0.97
Number of adults in the household						
1	–			1.00		
2	–			1.47	1.41	1.52
3 and above				1.27	1.22	1.31
Number of children in the household						
0	1.00			1.00		
1	1.00	0.92	1.08	1.09	1.06	1.12
2	0.96	0.87	1.05	0.94	0.91	0.97
3 and above	0.89	0.81	0.99	0.79	0.75	0.83
Living with someone who had alcohol problem						
No	1.00			1.00		
Yes	0.77	0.69	0.86	0.63	0.59	0.67
Living with someone who had depression						
No	1.00			1.00		
Yes	0.88	0.82	0.94	0.70	0.68	0.71
Living with someone who had serious mental illness						
No	–			1.00		
Yes	–			0.88	0.79	0.99
Special Education Need						
No	1.00			1.00		
Yes	0.12	0.11	0.13	0.14	0.14	0.15

*Intercept for FSM model: 0.24(0.20–0.29) and non-FSM model: 0.51(0.47–0.55).

### 3.5 Interaction between FSM and overall WIMD

Supplementary analysis describes the interaction between FSM eligibility and overall area-level deprivation as measured by WIMD (Supplementary Material Tables 2 and 3). The interaction model showed that FSM children in the least deprived areas were significantly more likely to achieve PLP than FSM children from the most deprived areas (aOR – 2.19 (1.92–2.50)). It also showed that FSM children living in the most deprived areas were less likely to achieve PLP than non-FSM children from similar areas.

## 4 Discussion

This study found that the area in which children grow up has an important impact on their developmental outcomes, especially at school, suggesting a neighbourhood effect on education irrespective or parental educational attainment (McDool, 2017). Previous research suggested a relatively small association between neighbourhood effects and educational attainment and that family background is more of a factor (Gibbons, 2002). However, the findings from our study suggest that area level improvements have a positive impact on the outcome of the children, and it can moderate the effect of household level deprivation (Fig. 2, Fig. 3). This trend is significant even after adjusting for other household-level factors (Table 3, Table 4). This study highlights specific aspects of neighbourhood characteristics e.g. community safety, area income and connectivity, which impact on children overcoming negative aspects of poverty. In terms of community safety, previous studies have showed that children are more able to undertake outdoor physical activity if they are living in a safer place and this directly contributes to their resilience (Flouri et al., 2014; Veitch et al., 2013). Other evidence indicates that concerns over community safety are a growing reason for dissatisfaction with green spaces (Welsh Government, 2018). Residents of deprived areas are more likely to report poorer safety in green spaces and visit them less frequently (Jones et al., 2009) with potential indirect consequences for physical development of children. Living in an area which feels unsafe due to high crime levels, has a detrimental effect on residents in general (Foster et al., 2013), hence living in an area with minimal crime risk becomes beneficial for child development. Evidence-based measures that improve area safely include neighbourhood watch, street lighting, CCTV, hotspots policing and alley gating (Crime Reduction Toolkit | College of Policing). In addition, this study also found that good access to services such as public transport, food shops, schools, leisure centres and health services are important aspects of the local area that helps children who are in poverty to achieve PLP in their life. There has been evidence that children living in an area with good access to services in day-to-day life has a positive influence on their overall development (Christian et al., 2015). An area with good public transport and good social connectivity is an advantageous environment for the children. This might explain why children in rural areas are less likely to achieve PLP than children in urban areas. The Income domain of WIMD reflects the proportion of the people who are living in the area who are claiming income-related benefits and qualitative evidence indicates that poverty also has an effect on children's experiences at school (Horgan, 2007). This study found that children who are in poverty (indicated by eligibility for FSM) do better when living in an area where fewer people are claiming benefits (e.g., less income-related deprivation in the area). This is also supported by a previous study which shows that if children in poverty have relocated to a less poor areas at an early stage, there is a decrease in the risk of adverse consequences in later life (Chetty et al., 2016). If the social norm is to be in employment this may make it also the ‘norm’ for children to remain in education or seek employment. The additional analysis conducted by the study found that the effect of local area on the child's educational attainment is clearer than its effect on child's mental health or alcohol or substance misuse, particularly for the children in household level deprivation (FSM children). This indicates that mental health and substance misuse might be more associated with individual level deprivation and factors within the family rather than local area and where are education is strongly associated with area level factors. This complex relationship needs further investigation.

This study brings together anonymously linked, routinely collected administrative datasets to build a nationally representative cohort of children and followed the study population longitudinally since birth till they complete KS4. This linked routine data framework facilitates the record linkage for the study population across health, education, and household level data. This is a major strength of the current study as this helps to overcome the limitations of selection and recall bias which are persistent in survey data. Also, data such as WIMD score, education record, FSM eligibility that were used to build the models in the study are available to government and policy-making bodies, hence these models can be exploited for developing intervention plans. However, the limitation of the study can be explained as this study uses person-level data to identify possible impact of non-income-based factors on child development and education outcomes. Aside from proxies for child poverty, such as FSM eligibility, the results indicate the effects of community safety, higher relative income, and access to services in an area on children's ability to achieve PLP. In doing so this study utilises small-area level measures from WIMD which are linked to ONS census geographies. ONS census geographies are designed to maintain best practice is disclosure controls for UK census data and therefore necessarily mask household-level variations in WIMD characteristics. This will introduce an ecological inference fallacy where aggregated data were used as a basis for individuals to make an inference (Hsieh, 2016). Aggregated-level data may not necessarily always be a true reflection of an individual; hence this can be a limitation of the study. However, the findings highlight the impact of broader area-related factors on child development in conjunction with a family level deprivation measure (FSM). Additionally, there is a need for multilevel modelling at various levels such as – LSOA, household and school, which would help to investigate the association of various granular area-level factors on a child's PLP profile. More than 20% of eligible children did not have a full GP record between age 11–16, so were excluded from the study. Also, those who did not have a continuous record in SAIL (WDSD dataset) from age 6 months were excluded. These children may have different characteristics of those included in the study and we cannot extrapolate to children who may have moved in or out of Wales in their early life, and the impact this has on achieving PLP, this might introduce a selection bias in the study. In some cases, there is the possibility that people may select areas, such as those performing well academically may move to be closer to good schools/libraries and that it is the people who chose the area rather than the influence of the area that impacts on education outcomes.

In summary, children who grow up in poverty but have achieved PLP as defined by; achieving qualifications at age 16 and do not have a mental health diagnosis or substance misuse (including alcohol) problems, are those who live in an area with good community safety, have good public transport and access to services and live in an area where people are employed rather than on benefits. The findings of the study are indicative of the fact that intervention in various aspects of a local area such as improving safety, connectivity and more people at work might help local children to achieve PLP in terms of education, mental health and reducing risk-taking behaviours (alcohol/drug use).

## Ethical approval

This study was approved by the SAIL Databank independent Information Governance Review Panel (IGRP) (project number 0916 – WECC Phase 4).

## Funding

This research has been carried out as part of the ADR Wales programme of work. The ADR Wales programme of work is aligned to the priority themes as identified in the Welsh Government's national strategy: Prosperity for All. ADR Wales brings together data science experts at Swansea University Medical School, staff from the Wales Institute of Social and Economic Research, Data and Methods (WISERD) at Cardiff University and specialist teams within the Welsh Government to develop new evidence which supports Prosperity for All by using the SAIL Databank at Swansea University, to link and analyse anonymised data. ADR Wales is part of the Economic and Social Research Council (part of UK Research and Innovation) funded ADR UK (grant ES/S007393/1).

This work was also supported by the National Centre for Population Health and Well-Being Research (NCPHWR) which is funded by Health and Care Research Wales. This work was supported by Health Data Research UK which receives its funding from HDR UK Ltd (NIWA1) funded by the UK Medical Research Council, Engineering and Physical Sciences Research Council, Economic and Social Research Council, Department of Health and Social Care (England), Chief Scientist Office of the Scottish Government Health and Social Care Directorates, Health and Social Care Research and Development Division (Welsh Government), Public Health Agency (Northern Ireland), British Heart Foundation (BHF) and the Welcome Trust.

This work uses data provided by patients and collected by the NHS as part of their care and support. This study used anonymised data held in the Secure Anonymised Information Linkage (SAIL) Databank. We would like to acknowledge all the data providers who enable SAIL to make anonymised data available for research.

## Authors’ contributions

All authors contributed to the study conception and design. Data collection, preparation, and analysis were performed by Amrita Bandyopadhyay. The first draft of the manuscript was written by Amrita Bandyopadhyay. Tony Whiffen, Richard Fry and Sinead Brophy reviewed and edited the drafts. All authors read and approved the final manuscript. Conceptualization: Sinead Brophy and Amrita Bandyopadhyay; Methodology: Amrita Bandyopadhyay and Sinead Brophy; Formal analysis and investigation: Amrita Bandyopadhyay Writing - original draft preparation: Amrita Bandyopadhyay; Writing - review and editing: Tony Whiffen, Richard Fry and Sinead Brophy, Supervision: Sinead Brophy.

## Participant consent

The study did not require participant consent as it utilises the anonymised data.

## Patient and public involvement statement

No patient involved.

## The original protocol

Not applicable.

## STROBE checklist

STROBE checklist has been added as a Supplementary file (Supplementary material STROBE checklist).

## Data sharing statement

The data have been archived in the Secure Anonymised Information Linkage Databank (https://saildatabank.com/0029).

## Declaration of competing interest

The authors declare the following financial interests/personal relationships which may be considered as potential competing interests: Prof Sinead Brophy reports financial support was provided by Administrative Data Research Wales.

## Data Availability

Data will be made available on request.
