# Supplementary material for: How does the local area deprivation influence life chances for children in poverty in Wales: A record linkage cohort study
Source: SSM Popul Health. 2023 Feb 23;22:101370. doi: 10.1016/j.ssmph.2023.101370 (PMC9986621; doi:10.1016/j.ssmph.2023.101370)
Supplement: Multimedia component 1 [file mmc1.pdf]

Table 1: Depression related ICD10 codes

**ICD10 codes**	**Descriptions**
F251	Schizoaffective disorder depressive type
F32	Depressive episode
F320	Mild depressive episode
F321	Moderate depressive episode
F322	Severe depressive episode without psychotic symptoms
F323	Severe depressive episode with psychotic symptoms
F328	Other depressive episodes
F329	Depressive episode, unspecified
F33	Recurrent depressive disorder
F330	Recurrent depressive disorder, current episode mild
F331	Recurrent depressive disorder, current episode moderate
F332	Recurrent depressive disorder, current episode severe without psychotic symptoms
F333	Recurrent depressive disorder, current episode severe with psychotic symptoms
F334	Recurrent depressive disorder, currently in remission
F338	Other recurrent depressive disorders
F339	Recurrent depressive disorder, unspecified
F341	Dysthymia

Table 2: Depression related READ codes

RAED codes	Descriptions
1B17.	Depressed
1B1U.	Symptoms of depression
1BQ..	Loss of capacity for enjoyment
1BT..	Depressed mood
1BU..	Loss of hope for the future
2257	O/E - depressed
E0013	Presenile dementia with depression
E0021	Senile dementia with depression
E112.	Single major depressive episode
E1120	Single major depressive episode, unspecified
E1121	Single major depressive episode, mild
E1122	Single major depressive episode, moderate
E1123	Single major depressive episode, severe, without psychosis
E1124	Single major depressive episode, severe, with psychosis
E1125	Single major depressive episode, partial or unspec remission
E1126	Single major depressive episode, in full remission
E112z	Single major depressive episode NOS
E113.	Recurrent major depressive episode
E1130	Recurrent major depressive episodes, unspecified
E1131	Recurrent major depressive episodes, mild

E1132	Recurrent major depressive episodes, moderate
E1133	Recurrent major depressive episodes, severe, no psychosis
E1134	Recurrent major depressive episodes, severe, with psychosis
E1135	Recurrent major depressive episodes,partial/unspec remission
E1136	Recurrent major depressive episodes, in full remission
E1137	Recurrent depression
E113z	Recurrent major depressive episode NOS
E118.	Seasonal affective disorder
E11y2	Atypical depressive disorder
E11z2	Masked depression
E130.	Reactive depressive psychosis
E135.	Agitated depression
E2003	Anxiety with depression
E204.	Neurotic depression reactive type
E291.	Prolonged depressive reaction
E2B..	Depressive disorder NEC
E2B0.	Postviral depression
E2B1.	Chronic depression
Eu204	[X]Post-schizophrenic depression
Eu251	[X]Schizoaffective disorder, depressive type
Eu32.	[X]Depressive episode
Eu320	[X]Mild depressive episode
Eu321	[X]Moderate depressive episode
Eu322	[X]Severe depressive episode without psychotic symptoms
Eu323	[X]Severe depressive episode with psychotic symptoms
Eu324	[X]Mild depression
Eu325	[X]Major depression, mild
Eu326	[X]Major depression, moderately severe
Eu327	[X]Major depression, severe without psychotic symptoms
Eu328	[X]Major depression, severe with psychotic symptoms
Eu32y	[X]Other depressive episodes
Eu32z	[X]Depressive episode, unspecified
Eu33.	[X]Recurrent depressive disorder
Eu330	[X]Recurrent depressive disorder, current episode mild
Eu331	[X]Recurrent depressive disorder, current episode moderate
Eu332	[X]Recurr depress disorder cur epi severe without psyc sympt
Eu333	[X]Recurrent depress disorder cur epi severe with psyc symp
Eu334	[X]Recurrent depressive disorder, currently in remission
Eu33y	[X]Other recurrent depressive disorders
Eu33z	[X]Recurrent depressive disorder, unspecified
Eu341	[X]Dysthymia
Eu412	[X]Mixed anxiety and depressive disorder

Table 3: Depression - medication READ codes

RAED codes	Descriptions
d11..	CHLORAL HYDRATE
d12..	CLOMETHIAZOLE EDISYLATE [HYPNOTIC]
d13..	*DICHLORALPHENAZONE
d14..	*FLUNITRAZEPAM
d15..	FLURAZEPAM
d16..	LOPRAZOLAM
d17..	LORMETAZEPAM
d18..	NITRAZEPAM
d1a..	TEMAZEPAM [HYPNOTIC]
d1b..	*TRIAZOLAM
d1c..	TRICLOFOS SODIUM
d1d..	ZOPICLONE
d1f..	ZOLPIDEM
d1g..	ZALEPLON
d1h..	MELATONIN
d21..	DIAZEPAM [ANXIOLYTIC]
d22..	ALPRAZOLAM
d23..	BROMAZEPAM
d24..	CHLORDIAZEPOXIDE
d25..	CHLORMEZANONE
d26..	CLOBAZAM
d27..	CLORAZEPATE DIPOTASSIUM
d28..	HYDROXYZINE HCL [ANXIOLYTIC]
d29..	*KETAZOLAM
d2a..	LORAZEPAM [ANXIOLYTIC]
d2b..	*MEDAZEPAM
d2c..	MEPROBAMATE
d2d..	OXAZEPAM
d2e..	*PRAZEPAM
d2f..	BUSPIRONE HYDROCHLORIDE
d2g..	FLUMAZENIL
d71..	AMITRIPTYLINE HYDROCHLORIDE [ANTIDEPRESSANT]
d72..	*BUTRIPTYLINE
d73..	CLOMIPRAMINE HYDROCHLORIDE
d74..	DESIPRAMINE HYDROCHLORIDE
d75..	DOSULEPIN HYDROCHLORIDE
d76..	DOXEPIN
d77..	IMIPRAMINE HYDROCHLORIDE [ANTIDEPRESSANT]
d78..	IPRINDOLE
d79..	LOFEPRAMINE
d7a..	MAPROTILINE HYDROCHLORIDE
d7b..	MIANSERIN HYDROCHLORIDE

d7c..	NORTRIPTYLINE
d7d..	PROTRIPTYLINE HYDROCHLORIDE
d7e..	TRAZODONE HYDROCHLORIDE
d7f..	TRIMIPRAMINE
d7g..	VILOXAZINE HYDROCHLORIDE
d7h..	AMOXAPINE
d81..	PHENELZINE
d83..	ISOCARBOXAZID
d84..	TRANYLCYPROMINE
d85..	MOCLOBEMIDE
d91..	COMPOUND ANTIDEPRESSANTS A-Z
da1..	FLUPENTIXOL [ANTIDEPRESSANT]
da2..	TRYPTOPHAN
da3..	FLUVOXAMINE MALEATE
da4..	FLUOXETINE HYDROCHLORIDE
da5..	SERTRALINE HYDROCHLORIDE
da6..	PAROXETINE HYDROCHLORIDE
da7..	VENLAFAXINE
da9..	CITALOPRAM
daA..	REBOXETINE
daB..	MIRTAZAPINE
daC..	ESCITALOPRAM
daD..	AGOMELATINE
gde..	DULOXETINE

Table 4: Serious Mental Illness related ICD10 codes

**ICD10 codes**	**Descriptions**
F200	Paranoid schizophrenia
F201	Hebephrenic schizophrenia
F202	Catatonic schizophrenia
F203	Undifferentiated schizophrenia
F204	Post-schizophrenic depression
F205	Residual schizophrenia
F206	Simple schizophrenia
F208	Other schizophrenia
F209	Schizophreniaunspecified
F21X	Schizotypal disorder
F220	Delusional disorder
F228	Other persistent delusional disorders
F229	Persistent delusional disorderunspecified
F230	Acute polymorphic psychot disord without symp of schizoph'a
F231	Acute polymorphic psychot disord with symp of schizophrenia
F232	Acute schizophrenia-like psychotic disorder

F233	Other acute predominantly delusional psychotic disorders
F238	Other acute and transient psychotic disorders
F239	Acute and transient psychotic disorderunspecified
F24X	Induced delusional disorder
F250	Schizoaffective disordermanic type
F251	Schizoaffective disorderdepressive type
F252	Schizoaffective disordermixed type
F258	Other schizoaffective disorders
F259	Schizoaffective disorderunspecified
F28X	Other nonorganic psychotic disorders
F29X	Unspecified nonorganic psychosis
F300	Hypomania
F301	Mania without psychotic symptoms
F302	Mania with psychotic symptoms
F308	Other manic episodes
F309	Manic episodeunspecified
F310	Bipolar affective disordercurrent episode hypomanic
F311	Bipolar affect disord cur epi manic without psychotic symp
F312	Bipolar affect disorder cur epi manic with psychotic symp
F313	Bipolar affect disorder cur epi mild or moderate depression
F314	Bipolar affect disord cur epi sev depres without psyc symp
F315	Bipolar affect disord cur epi severe depres with psyc symp
F316	Bipolar affective disordercurrent episode mixed
F317	Bipolar affective disordercurrently in remission
F318	Other bipolar affective disorders
F319	Bipolar affective disorderunspecified
F323	Severe depressive episode with psychotic symptoms
F333	Recurrent depress disorder cur epi severe with psyc symp
F39X	Unspecified mood [affective] disorder

Table 5: Serious mental Illness related READ codes

**RAED codes**	**Descriptions**
E10..	Schizophrenic disorders
E100.	Simple schizophrenia
E1000	Unspecified schizophrenia
E1001	Subchronic schizophrenia
E1002	Chronic schizophrenic
E1003	Acute exacerbation of subchronic schizophrenia
E1004	Acute exacerbation of chronic schizophrenia
E1005	Schizophrenia in remission
E100z	Simple schizophrenia NOS
E101.	Hebephrenic schizophrenia
E1010	Unspecified hebephrenic schizophrenia

E1011	Subchronic hebephrenic schizophrenia
E1012	Chronic hebephrenic schizophrenia
E1013	Acute exacerbation of subchronic hebephrenic schizophrenia
E1014	Acute exacerbation of chronic hebephrenic schizophrenia
E1015	Hebephrenic schizophrenia in remission
E101z	Hebephrenic schizophrenia NOS
E102.	Catatonic schizophrenia
E1020	Unspecified catatonic schizophrenia
E1021	Subchronic catatonic schizophrenia
E1022	Chronic catatonic schizophrenia
E1023	Acute exacerbation of subchronic catatonic schizophrenia
E1024	Acute exacerbation of chronic catatonic schizophrenia
E1025	Catatonic schizophrenia in remission
E102z	Catatonic schizophrenia NOS
E103.	Paranoid schizophrenia
E1030	Unspecified paranoid schizophrenia
E1031	Subchronic paranoid schizophrenia
E1032	Chronic paranoid schizophrenia
E1033	Acute exacerbation of subchronic paranoid schizophrenia
E1034	Acute exacerbation of chronic paranoid schizophrenia
E1035	Paranoid schizophrenia in remission
E103z	Paranoid schizophrenia NOS
E104.	Acute schizophrenic episode
E105.	Latent schizophrenia
E1050	Unspecified latent schizophrenia
E1051	Subchronic latent schizophrenia
E1052	Chronic latent schizophrenia
E1053	Acute exacerbation of subchronic latent schizophrenia
E1054	Acute exacerbation of chronic latent schizophrenia
E1055	Latent schizophrenia in remission
E105z	Latent schizophrenia NOS
E106.	Residual schizophrenia
E107.	Schizo-affective schizophrenia
E1070	Unspecified schizo-affective schizophrenia
E1071	Subchronic schizo-affective schizophrenia
E1072	Chronic schizo-affective schizophrenia
E1073	Acute exacerbation subchronic schizo-affective schizophrenia
E1074	Acute exacerbation of chronic schizo-affective schizophrenia
E1075	Schizo-affective schizophrenia in remission
E107z	Schizo-affective schizophrenia NOS
E10y.	Other schizophrenia
E10y0	Atypical schizophrenia
E10y1	Coenesthopathic schizophrenia
E10yz	Other schizophrenia NOS

E10z.	Schizophrenia NOS
E110.	Manic disorder, single episode
E1100	Single manic episode, unspecified
E1101	Single manic episode, mild
E1102	Single manic episode, moderate
E1103	Single manic episode, severe without mention of psychosis
E1104	Single manic episode, severe, with psychosis
E1105	Single manic episode in partial or unspecified remission
E1106	Single manic episode in full remission
E110z	Manic disorder, single episode NOS
E111.	Recurrent manic episodes
E1110	Recurrent manic episodes, unspecified
E1111	Recurrent manic episodes, mild
E1112	Recurrent manic episodes, moderate
E1113	Recurrent manic episodes, severe without mention psychosis
E1114	Recurrent manic episodes, severe, with psychosis
E1115	Recurrent manic episodes, partial or unspecified remission
E1116	Recurrent manic episodes, in full remission
E111z	Recurrent manic episode NOS
E1124	Single major depressive episode, severe, with psychosis
E1134	Recurrent major depressive episodes, severe, with psychosis
E114.	Bipolar affective disorder, currently manic
E1140	Bipolar affective disorder, currently manic, unspecified
E1141	Bipolar affective disorder, currently manic, mild
E1142	Bipolar affective disorder, currently manic, moderate
E1143	Bipolar affect disord, currently manic, severe, no psychosis
E1144	Bipolar affect disord, currently manic,severe with psychosis
E1145	Bipolar affect disord,currently manic, part/unspec remission
E1146	Bipolar affective disorder, currently manic, full remission
E114z	Bipolar affective disorder, currently manic, NOS
E115.	Bipolar affective disorder, currently depressed
E1150	Bipolar affective disorder, currently depressed, unspecified
E1151	Bipolar affective disorder, currently depressed, mild
E1152	Bipolar affective disorder, currently depressed, moderate
E1153	Bipolar affect disord, now depressed, severe, no psychosis
E1154	Bipolar affect disord, now depressed, severe with psychosis
E1155	Bipolar affect disord, now depressed, part/unspec remission
E1156	Bipolar affective disorder, now depressed, in full remission
E115z	Bipolar affective disorder, currently depressed, NOS
E116.	Mixed bipolar affective disorder
E1160	Mixed bipolar affective disorder, unspecified
E1161	Mixed bipolar affective disorder, mild
E1162	Mixed bipolar affective disorder, moderate
E1163	Mixed bipolar affective disorder, severe, without psychosis

E1164	Mixed bipolar affective disorder, severe, with psychosis
E1165	Mixed bipolar affective disorder, partial/unspec remission
E1166	Mixed bipolar affective disorder, in full remission
E116z	Mixed bipolar affective disorder, NOS
E117.	Unspecified bipolar affective disorder
E1170	Unspecified bipolar affective disorder, unspecified
E1171	Unspecified bipolar affective disorder, mild
E1172	Unspecified bipolar affective disorder, moderate
E1173	Unspecified bipolar affective disorder, severe, no psychosis
E1174	Unspecified bipolar affective disorder,severe with psychosis
E1175	Unspecified bipolar affect disord, partial/unspec remission
E1176	Unspecified bipolar affective disorder, in full remission
E117z	Unspecified bipolar affective disorder, NOS
E11y.	Other and unspecified manic-depressive psychoses
E11y0	Unspecified manic-depressive psychoses
E11y1	Atypical manic disorder
E11y3	Other mixed manic-depressive psychoses
E11yz	Other and unspecified manic-depressive psychoses NOS
E11z.	Other and unspecified affective psychoses
E11z0	Unspecified affective psychoses NOS
E11zz	Other affective psychosis NOS
E12..	Paranoid states
E120.	Simple paranoid state
E121.	Chronic paranoid psychosis
E122.	Paraphrenia
E123.	Shared paranoid disorder
E12y.	Other paranoid states
E12y0	Paranoia querulans
E12yz	Other paranoid states NOS
E12z.	Paranoid psychosis NOS
E13..	Other nonorganic psychoses
E130.	Reactive depressive psychosis
E131.	Acute hysterical psychosis
E132.	Reactive confusion
E133.	Acute paranoid reaction
E134.	Psychogenic paranoid psychosis
E13y.	Other reactive psychoses
E13y0	Psychogenic stupor
E13y1	Brief reactive psychosis
E13yz	Other reactive psychoses NOS
E13z.	Nonorganic psychosis NOS
E2122	Schizotypal personality
Eu2..	[X]Schizophrenia, schizotypal and delusional disorders
Eu20.	[X]Schizophrenia

Eu200	[X]Paranoid schizophrenia
Eu201	[X]Hebephrenic schizophrenia
Eu202	[X]Catatonic schizophrenia
Eu203	[X]Undifferentiated schizophrenia
Eu204	[X]Post-schizophrenic depression
Eu205	[X]Residual schizophrenia
Eu206	[X]Simple schizophrenia
Eu20y	[X]Other schizophrenia
Eu20z	[X]Schizophrenia, unspecified
Eu21.	[X]Schizotypal disorder
Eu22.	[X]Persistent delusional disorders
Eu220	[X]Delusional disorder
Eu221	[X]Delusional misidentification syndrome
Eu222	[X]Cotard syndrome
Eu223	[X]Paranoid state in remission
Eu22y	[X]Other persistent delusional disorders
Eu22z	[X]Persistent delusional disorder, unspecified
Eu23.	[X]Acute and transient psychotic disorders
Eu230	[X]Acute polymorphic psychot disord without symp of schizoph
Eu231	[X]Acute polymorphic psychot disord with symp of schizophren
Eu232	[X]Acute schizophrenia-like psychotic disorder
Eu233	[X]Other acute predominantly delusional psychotic disorders
Eu23y	[X]Other acute and transient psychotic disorders
Eu23z	[X]Acute and transient psychotic disorder, unspecified
Eu24.	[X]Induced delusional disorder
Eu25.	[X]Schizoaffective disorders
Eu250	[X]Schizoaffective disorder, manic type
Eu251	[X]Schizoaffective disorder, depressive type
Eu252	[X]Schizoaffective disorder, mixed type
Eu25y	[X]Other schizoaffective disorders
Eu25z	[X]Schizoaffective disorder, unspecified
Eu26.	[X]Nonorganic psychosis in remission
Eu2y.	[X]Other nonorganic psychotic disorders
Eu2z.	[X]Unspecified nonorganic psychosis
Eu30.	[X]Manic episode
Eu300	[X]Hypomania
Eu301	[X]Mania without psychotic symptoms
Eu302	[X]Mania with psychotic symptoms
Eu30y	[X]Other manic episodes
Eu30z	[X]Manic episode, unspecified
Eu31.	[X]Bipolar affective disorder
Eu310	[X]Bipolar affective disorder, current episode hypomanic
Eu311	[X]Bipolar affect disorder cur epi manic wout psychotic symp
Eu312	[X]Bipolar affect disorder cur epi manic with psychotic symp

Eu313	[X]Bipolar affect disorder cur epi mild or moderate depressn
Eu314	[X]Bipol aff disord, curr epis sev depress, no psychot symp
Eu315	[X]Bipolar affect dis cur epi severe depres with psyc symp
Eu316	[X]Bipolar affective disorder, current episode mixed
Eu317	[X]Bipolar affective disorder, currently in remission
Eu318	[X]Bipolar affective disorder type I
Eu319	[X]Bipolar affective disorder type II
Eu31y	[X]Other bipolar affective disorders
Eu31z	[X]Bipolar affective disorder, unspecified
Eu323	[X]Severe depressive episode with psychotic symptoms
Eu333	[X]Recurrent depress disorder cur epi severe with psyc symp
