# Supplementary material for: How does the local area deprivation influence life chances for children in poverty in Wales: A record linkage cohort study
Source: SSM Popul Health. 2023 Feb 23;22:101370. doi: 10.1016/j.ssmph.2023.101370 (PMC9986621; doi:10.1016/j.ssmph.2023.101370)
Supplement: Multimedia component 2 [file mmc2.pdf]

Table 1: Alcohol related ICD10 codes

ICD10 Codes	Descriptions
E244	Alcohol-induced pseudo-Cushing's syndrome
E512	Wernicke's encephalopathy
F10	Mental and behavioural disorders due to use of alcohol
F100	Mental and behavioural disorders due to use of alcohol
F101	Mental and behavioural disorders due to use of alcohol
F102	Mental and behavioural disorders due to use of alcohol
F103	Mental and behavioural disorders due to use of alcohol
F104	Mental and behavioural disorders due to use of alcohol
F105	Mental and behavioural disorders due to use of alcohol
F106	Mental and behavioural disorders due to use of alcohol
F107	Mental and behavioural disorders due to use of alcohol
F108	Mental and behavioural disorders due to use of alcohol
F109	Mental and behavioural disorders due to use of alcohol
G312	Degeneration of nervous system due to alcohol
G621	Alcoholic polyneuropathy
G721	Alcoholic myopathy
I426	Alcoholic cardiomyopathy
K292	Alcoholic gastritis
K70	Alcoholic liver disease
K700	Alcoholic fatty liver
K701	Alcoholic hepatitis
K702	Alcoholic fibrosis and sclerosis of liver
K703	Alcoholic cirrhosis of liver
K704	Alcoholic hepatic failure
K709	Alcoholic liver disease, unspecified
K852	Alcohol-induced acute pancreatitis
K860	Alcohol-induced chronic pancreatitis
O354	Maternal care for (suspected) damage to fetus from alcohol
R780	Finding of alcohol in blood
T51	Toxic effect of alcohol
T510	Toxic effect: Ethanol
T511	Toxic effect: Methanol
T512	Toxic effect: 2-Propanol
T513	Toxic effect: Fusel oil
T518	Toxic effect: Other alcohols
T519	Toxic effect: Alcohol, unspecified
X45	Accidental poisoning by and exposure to alcohol
X450	Accidental poisoning by and exposure to alcohol
X451	Accidental poisoning by and exposure to alcohol
X452	Accidental poisoning by and exposure to alcohol
X453	Accidental poisoning by and exposure to alcohol
X454	Accidental poisoning by and exposure to alcohol

X455	Accidental poisoning by and exposure to alcohol
X456	Accidental poisoning by and exposure to alcohol
X457	Accidental poisoning by and exposure to alcohol
X458	Accidental poisoning by and exposure to alcohol
X459	Accidental poisoning by and exposure to alcohol
X65	Intentional self-poisoning by and exposure to alcohol
X650	Intentional self-poisoning by and exposure to alcohol
X651	Intentional self-poisoning by and exposure to alcohol
X652	Intentional self-poisoning by and exposure to alcohol
X653	Intentional self-poisoning by and exposure to alcohol
X654	Intentional self-poisoning by and exposure to alcohol
X655	Intentional self-poisoning by and exposure to alcohol
X656	Intentional self-poisoning by and exposure to alcohol
X657	Intentional self-poisoning by and exposure to alcohol
X658	Intentional self-poisoning by and exposure to alcohol
X659	Intentional self-poisoning by and exposure to alcohol
Y15	Poisoning by and exposure to alcohol, undetermined intent
Y150	Poisoning by and exposure to alcohol, undetermined intent
Y151	Poisoning by and exposure to alcohol, undetermined intent
Y152	Poisoning by and exposure to alcohol, undetermined intent
Y153	Poisoning by and exposure to alcohol, undetermined intent
Y154	Poisoning by and exposure to alcohol, undetermined intent
Y155	Poisoning by and exposure to alcohol, undetermined intent
Y156	Poisoning by and exposure to alcohol, undetermined intent
Y157	Poisoning by and exposure to alcohol, undetermined intent
Y158	Poisoning by and exposure to alcohol, undetermined intent
Y159	Poisoning by and exposure to alcohol, undetermined intent
Y573	Alcohol deterrents
Y900	Blood alcohol level of less than 20 mg/100 ml
Y901	Blood alcohol level of 20-39 mg/100 ml
Y902	Blood alcohol level of 40-59 mg/100 ml
Y903	Blood alcohol level of 60-79 mg/100 ml
Y904	Blood alcohol level of 80-99 mg/100 ml
Y905	Blood alcohol level of 100-119 mg/100 ml
Y906	Blood alcohol level of 120-199 mg/100 ml
Y907	Blood alcohol level of 200-239 mg/100 ml
Y908	Blood alcohol level of 240 mg/100 ml or more
Y909	Presence of alcohol in blood, level not specified
Y910	Mild alcohol intoxication
Y911	Moderate alcohol intoxication
Y912	Severe alcohol intoxication
Y913	Very severe alcohol intoxication
Y919	Alcohol involvement, not otherwise specified
Z502	Alcohol rehabilitation

Z714	Alcohol abuse counselling and surveillance
Z721	Alcohol use
