# Supplementary material for: How does the local area deprivation influence life chances for children in poverty in Wales: A record linkage cohort study
Source: SSM Popul Health. 2023 Feb 23;22:101370. doi: 10.1016/j.ssmph.2023.101370 (PMC9986621; doi:10.1016/j.ssmph.2023.101370)
Supplement: Multimedia component 3 [file mmc3.pdf]

Table 1: ADHD related READ codes

READ codes	Descriptions
6A61.	ADHD annual review
8BPT.	Drug therapy for ADHD
8BPT0	Stimulant drug therapy for ADHD
8BPT1	Non-stimulant drug therapy for ADHD
9Ngp.	On drug ther ADHD (attention deficit hyperactivity disorder)
9Ngp0	On stim drug ther ADHD (attention def hyperactivity disordr)
9Ngp1	On non-stimulant drug therapy for ADHD
9Ol8.	ADHD monitoring invitation first letter
9Ol9.	ADHD monitoring invitation second letter
9OlA.	ADHD monitoring invitation third letter
E2E..	Childhood hyperkinetic syndrome
E2E0.	Child attention deficit disorder
E2E00	Attention deficit without hyperactivity
E2E01	Attention deficit with hyperactivity
E2E0z	Child attention deficit disorder NOS
E2E2.	Hyperkinetic conduct disorder
E2Ez.	Hyperkinetic syndrome NOS
Eu90.	[X]Hyperkinetic disorders
Eu900	[X]Disturbance activity/attntn
Eu901	[X]Hyperkinetic conduct disord
Eu90y	[X]Oth hyperkinetic disorders
Eu90z	[X]Hyperkinetic disorder, unsp
Eu9y7	[X]Attention deficit disorder
dc1..	DEXAMFETAMINE SULPHATE
dc11.	*DEXEDRINE 5mg tablets
dc1v.	DEXAMFET SULF 1mg/mL oral soln
dc1w.	DEXAMFETAMINE SULPH 5mg tabs
dc1x.	*DEXAMPHETAMINE 7.5mg m/r caps
dc1y.	*DEXAMPHETAMINE 12.5mg caps
dc1z.	*DEXAMPHETAMINE 20mg m/r caps
dw...	DRUGS USED TO TREAT HYPERACTIVITY DISORDERS
dw1..	METHYLPHENIDATE
dw11.	METHYLPHENIDATE HCL 10mg tabs
dw12.	RITALIN 10mg tablets
dw13.	*EQUASYM 5mg tablets
dw14.	*EQUASYM 20mg tablets
dw15.	*EQUASYM 10mg tablets
dw16.	EQUASYM XL 20mg m/r capsules
dw17.	CONCERTA XL 18mg m/r tablets
dw18.	CONCERTA XL 36mg m/r tablets
dw1C.	EQUASYM XL 10mg m/r capsules
dw1D.	EQUASYM XL 30mg m/r capsules

dw1E.	MEDIKINET XL 10mg m/r capsules
dw1F.	MEDIKINET XL 20mg m/r capsules
dw1G.	MEDIKINET XL 30mg m/r capsules
dw1H.	MEDIKINET XL 40mg m/r capsules
dw1I.	CONCERTA XL 27mg m/r tablets
dw1J.	MEDIKINET 5mg tablets
dw1K.	MEDIKINET 10mg tablets
dw1L.	MEDIKINET 20mg tablets
dw1M.	MEDIKINET XL 5mg m/r capsules
dw1N.	MEDIKINET XL 50mg m/r capsules
dw1O.	MEDIKINET XL 60mg m/r capsules
dw1U.	CONCERTA XL 54mg m/r tablets
dw1n.	METHYLPHENIDATE 54mg m/r tabs
dw1o.	METHYLPHENIDATE 50mg m/r caps
dw1p.	METHYLPHENIDATE 60mg m/r caps
dw1q.	METHYLPHENIDATE 5mg m/r caps
dw1r.	METHYLPHENIDATE 27mg m/r tabs
dw1s.	METHYLPHENIDATE 40mg m/r caps
dw1t.	METHYLPHENIDATE 10mg m/r caps
dw1u.	METHYLPHENIDATE 30mg m/r caps
dw1v.	METHYLPHENIDATE 36mg m/r tabs
dw1w.	METHYLPHENIDATE 18mg m/r tabs
dw1x.	METHYLPHENIDATE 20mg m/r caps
dw1y.	METHYLPHENIDATE HCL 5mg tabs
dw1z.	METHYLPHENIDATE HCL 20mg tabs
dw2..	ATOMOXETINE
dw21.	STRATTERA 10mg capsules
dw22.	STRATTERA 18mg capsules
dw23.	STRATTERA 25mg capsules
dw24.	STRATTERA 40mg capsules
dw25.	STRATTERA 60mg capsules
dw26.	STRATTERA 80mg capsules
dw27.	STRATTERA 100mg capsules
dw28.	STRATTERA 4mg/mL oral solution
dw2s.	ATOMOXETINE 4mg/mL oral soln
dw2t.	ATOMOXETINE 100mg capsules
dw2u.	ATOMOXETINE 80mg capsules
dw2v.	ATOMOXETINE 60mg capsules
dw2w.	ATOMOXETINE 40mg capsules
dw2x.	ATOMOXETINE 25mg capsules
dw2y.	ATOMOXETINE 18mg capsules
dw2z.	ATOMOXETINE 10mg capsules
dw3..	LISDEXAMFETAMINE
dw3x.	LISDEXAMFETAMINE DIM 70mg caps

dw3y.	LISDEXAMFETAMINE DIM 50mg caps
dw3z.	LISDEXAMFETAMINE DIM 30mg caps

Table 2: Conduct Disorder related READ codes

READ codes	Descriptions
E2731	Head-banging
E293z	Adjustment reaction with predominant disturbance conduct NOS
E2C..	Disturbance of conduct NEC
E2C0.	Aggressive unsocial conduct disorder
E2C00	Aggressive outburst
E2C01	Anger reaction
E2C0z	Aggressive unsocial conduct disorder NOS
E2C1.	Nonaggressive unsocial conduct disorder
E2C10	Unsocial childhood truancy
E2C11	Solitary stealing
E2C12	Tantrums
E2C1z	Nonaggressive unsocial conduct disorder NOS
E2C2.	Socialised conduct disorder
E2C2z	Socialised conduct disorder NOS
E2C4z	Mixed disturbance of conduct and emotion NOS
E2Cy.	Other conduct disturbances
E2Cyz	Other conduct disturbances NOS
E2Cz.	Unspecified disturbance of conduct
E2Czz	Disturbance of conduct NOS
E2Dy0	Childhood and adolescent oppositional disorder
E2E2.	Hyperkinetic conduct disorder
Eu9..	[X]Behavioural/emotional disords onset childhood/adolescence
Eu901	[X]Hyperkinetic conduct disorder
Eu91.	[X]Conduct disorders
Eu910	[X]Conduct disorder confined to the family context
Eu911	[X]Unsocialized conduct disorder
Eu912	[X]Socialized conduct disorder
Eu913	[X]Oppositional defiant disorder
Eu91y	[X]Other conduct disorders
Eu91z	[X]Conduct disorder, unspecified
Eu92.	[X]Mixed disorders of conduct and emotions
Eu920	[X]Depressive conduct disorder
Eu92y	[X]Other mixed disorders of conduct and emotions
Eu92z	[X]Mixed disorder of conduct and emotions, unspecified
Eu941	[X]Reactive attachment disorder of childhood

Table 3: Self harm related ICD10 codes

ICD10 codes	Descriptions
X60	Inten self pois/expos to nonopiod analges antipy & antirheum
X600	Inten self pois/expos to nonopiod analges antipy & antirheum
X601	Inten self pois/expos to nonopiod analges antipy & antirheum
X602	Inten self pois/expos to nonopiod analges antipy & antirheum
X603	Inten self pois/expos to nonopiod analges antipy & antirheum
X604	Inten self pois/expos to nonopiod analges antipy & antirheum
X605	Inten self pois/expos to nonopiod analges antipy & antirheum
X606	Inten self pois/expos to nonopiod analges antipy & antirheum
X607	Inten self pois/expos to nonopiod analges antipy & antirheum
X608	Inten self pois/expos to nonopiod analges antipy & antirheum
X609	Inten self pois/expos to nonopiod analges antipy & antirheum
X61	Int sf pois/expo ant-epi sed-hyp ant park & psy'trop dgs NEC
X610	Int sf pois/expo ant-epi sed-hyp ant park & psy'trop dgs NEC
X611	Int sf pois/expo ant-epi sed-hyp ant park & psy'trop dgs NEC
X612	Int sf pois/expo ant-epi sed-hyp ant park & psy'trop dgs NEC
X613	Int sf pois/expo ant-epi sed-hyp ant park & psy'trop dgs NEC
X614	Int sf pois/expo ant-epi sed-hyp ant park & psy'trop dgs NEC
X615	Int sf pois/expo ant-epi sed-hyp ant park & psy'trop dgs NEC
X616	Int sf pois/expo ant-epi sed-hyp ant park & psy'trop dgs NEC
X617	Int sf pois/expo ant-epi sed-hyp ant park & psy'trop dgs NEC
X618	Int sf pois/expo ant-epi sed-hyp ant park & psy'trop dgs NEC
X619	Int sf pois/expo ant-epi sed-hyp ant park & psy'trop dgs NEC
X62	Inten sf pois/expos narcots & psy'dysleptics [halluc'ns]NEC
X620	Inten sf pois/expos narcots & psy'dysleptics [halluc'ns]NEC
X621	Inten sf pois/expos narcots & psy'dysleptics [halluc'ns]NEC
X622	Inten sf pois/expos narcots & psy'dysleptics [halluc'ns]NEC
X623	Inten sf pois/expos narcots & psy'dysleptics [halluc'ns]NEC
X624	Inten sf pois/expos narcots & psy'dysleptics [halluc'ns]NEC
X625	Inten sf pois/expos narcots & psy'dysleptics [halluc'ns]NEC
X626	Inten sf pois/expos narcots & psy'dysleptics [halluc'ns]NEC
X627	Inten sf pois/expos narcots & psy'dysleptics [halluc'ns]NEC
X628	Inten sf pois/expos narcots & psy'dysleptics [halluc'ns]NEC
X629	Inten sf pois/expos narcots & psy'dysleptics [halluc'ns]NEC
X63	Intent self-poison/expos oth drug act on autonom nervous sys
X630	Intent self-poison/expos oth drug act on autonom nervous sys
X631	Intent self-poison/expos oth drug act on autonom nervous sys
X632	Intent self-poison/expos oth drug act on autonom nervous sys
X633	Intent self-poison/expos oth drug act on autonom nervous sys
X634	Intent self-poison/expos oth drug act on autonom nervous sys
X635	Intent self-poison/expos oth drug act on autonom nervous sys

X636	Intent self-poison/expos oth drug act on autonom nervous sys
X637	Intent self-poison/expos oth drug act on autonom nervous sys
X638	Intent self-poison/expos oth drug act on autonom nervous sys
X639	Intent self-poison/expos oth drug act on autonom nervous sys
X64	Int self-poison/expos to oth & unsp drugs medics & biol subs
X640	Int self-poison/expos to oth & unsp drugs medics & biol subs
X641	Int self-poison/expos to oth & unsp drugs medics & biol subs
X642	Int self-poison/expos to oth & unsp drugs medics & biol subs
X643	Int self-poison/expos to oth & unsp drugs medics & biol subs
X644	Int self-poison/expos to oth & unsp drugs medics & biol subs
X645	Int self-poison/expos to oth & unsp drugs medics & biol subs
X646	Int self-poison/expos to oth & unsp drugs medics & biol subs
X647	Int self-poison/expos to oth & unsp drugs medics & biol subs
X648	Int self-poison/expos to oth & unsp drugs medics & biol subs
X649	Int self-poison/expos to oth & unsp drugs medics & biol subs
X65	Intentional self-poisoning by and exposure to alcohol
X650	Intentional self-poisoning by and exposure to alcohol
X651	Intentional self-poisoning by and exposure to alcohol
X652	Intentional self-poisoning by and exposure to alcohol
X653	Intentional self-poisoning by and exposure to alcohol
X654	Intentional self-poisoning by and exposure to alcohol
X655	Intentional self-poisoning by and exposure to alcohol
X656	Intentional self-poisoning by and exposure to alcohol
X657	Intentional self-poisoning by and exposure to alcohol
X658	Intentional self-poisoning by and exposure to alcohol
X659	Intentional self-poisoning by and exposure to alcohol
X66	Intent self-poison/expos org solvs+halogen hydrocarbon vapor
X660	Intent self-poison/expos org solvs+halogen hydrocarbon vapor
X661	Intent self-poison/expos org solvs+halogen hydrocarbon vapor
X662	Intent self-poison/expos org solvs+halogen hydrocarbon vapor
X663	Intent self-poison/expos org solvs+halogen hydrocarbon vapor
X664	Intent self-poison/expos org solvs+halogen hydrocarbon vapor
X665	Intent self-poison/expos org solvs+halogen hydrocarbon vapor
X666	Intent self-poison/expos org solvs+halogen hydrocarbon vapor
X667	Intent self-poison/expos org solvs+halogen hydrocarbon vapor
X668	Intent self-poison/expos org solvs+halogen hydrocarbon vapor
X669	Intent self-poison/expos org solvs+halogen hydrocarbon vapor
X67	Intent self-poisoning by and expos to oth gases and vapours
X670	Intent self-poisoning by and expos to oth gases and vapours
X671	Intent self-poisoning by and expos to oth gases and vapours
X672	Intent self-poisoning by and expos to oth gases and vapours
X673	Intent self-poisoning by and expos to oth gases and vapours
X674	Intent self-poisoning by and expos to oth gases and vapours
X675	Intent self-poisoning by and expos to oth gases and vapours

X676	Intent self-poisoning by and expos to oth gases and vapours
X677	Intent self-poisoning by and expos to oth gases and vapours
X678	Intent self-poisoning by and expos to oth gases and vapours
X679	Intent self-poisoning by and expos to oth gases and vapours
X68	Intentional self-poisoning by and exposure to pesticides
X680	Intentional self-poisoning by and exposure to pesticides
X681	Intentional self-poisoning by and exposure to pesticides
X682	Intentional self-poisoning by and exposure to pesticides
X683	Intentional self-poisoning by and exposure to pesticides
X684	Intentional self-poisoning by and exposure to pesticides
X685	Intentional self-poisoning by and exposure to pesticides
X686	Intentional self-poisoning by and exposure to pesticides
X687	Intentional self-poisoning by and exposure to pesticides
X688	Intentional self-poisoning by and exposure to pesticides
X689	Intentional self-poisoning by and exposure to pesticides
X69	Intent self-poison/expos oth and unspec chems and nox subs
X690	Intent self-poison/expos oth and unspec chems and nox subs
X691	Intent self-poison/expos oth and unspec chems and nox subs
X692	Intent self-poison/expos oth and unspec chems and nox subs
X693	Intent self-poison/expos oth and unspec chems and nox subs
X694	Intent self-poison/expos oth and unspec chems and nox subs
X695	Intent self-poison/expos oth and unspec chems and nox subs
X696	Intent self-poison/expos oth and unspec chems and nox subs
X697	Intent self-poison/expos oth and unspec chems and nox subs
X698	Intent self-poison/expos oth and unspec chems and nox subs
X699	Intent self-poison/expos oth and unspec chems and nox subs
X70	Intent self-harm by hanging strangulation and suffocation
X700	Intent self-harm by hanging strangulation and suffocation
X701	Intent self-harm by hanging strangulation and suffocation
X702	Intent self-harm by hanging strangulation and suffocation
X703	Intent self-harm by hanging strangulation and suffocation
X704	Intent self-harm by hanging strangulation and suffocation
X705	Intent self-harm by hanging strangulation and suffocation
X706	Intent self-harm by hanging strangulation and suffocation
X707	Intent self-harm by hanging strangulation and suffocation
X708	Intent self-harm by hanging strangulation and suffocation
X709	Intent self-harm by hanging strangulation and suffocation
X71	Intentional self-harm by drowning and submersion
X710	Intentional self-harm by drowning and submersion
X711	Intentional self-harm by drowning and submersion
X712	Intentional self-harm by drowning and submersion
X713	Intentional self-harm by drowning and submersion
X714	Intentional self-harm by drowning and submersion
X715	Intentional self-harm by drowning and submersion

X716	Intentional self-harm by drowning and submersion
X717	Intentional self-harm by drowning and submersion
X718	Intentional self-harm by drowning and submersion
X719	Intentional self-harm by drowning and submersion
X72	Intentional self-harm by handgun discharge
X720	Intentional self-harm by handgun discharge
X721	Intentional self-harm by handgun discharge
X722	Intentional self-harm by handgun discharge
X723	Intentional self-harm by handgun discharge
X724	Intentional self-harm by handgun discharge
X725	Intentional self-harm by handgun discharge
X726	Intentional self-harm by handgun discharge
X727	Intentional self-harm by handgun discharge
X728	Intentional self-harm by handgun discharge
X729	Intentional self-harm by handgun discharge
X73	Intent self-harm by rifle shotgun & larger firearm discharge
X730	Intent self-harm by rifle shotgun & larger firearm discharge
X731	Intent self-harm by rifle shotgun & larger firearm discharge
X732	Intent self-harm by rifle shotgun & larger firearm discharge
X733	Intent self-harm by rifle shotgun & larger firearm discharge
X734	Intent self-harm by rifle shotgun & larger firearm discharge
X735	Intent self-harm by rifle shotgun & larger firearm discharge
X736	Intent self-harm by rifle shotgun & larger firearm discharge
X737	Intent self-harm by rifle shotgun & larger firearm discharge
X738	Intent self-harm by rifle shotgun & larger firearm discharge
X739	Intent self-harm by rifle shotgun & larger firearm discharge
X74	Intent self-harm by other and unspecified firearm discharge
X740	Intent self-harm by other and unspecified firearm discharge
X741	Intent self-harm by other and unspecified firearm discharge
X742	Intent self-harm by other and unspecified firearm discharge
X743	Intent self-harm by other and unspecified firearm discharge
X744	Intent self-harm by other and unspecified firearm discharge
X745	Intent self-harm by other and unspecified firearm discharge
X746	Intent self-harm by other and unspecified firearm discharge
X747	Intent self-harm by other and unspecified firearm discharge
X748	Intent self-harm by other and unspecified firearm discharge
X749	Intent self-harm by other and unspecified firearm discharge
X75	Intentional self-harm by explosive material
X750	Intentional self-harm by explosive material
X751	Intentional self-harm by explosive material
X752	Intentional self-harm by explosive material
X753	Intentional self-harm by explosive material
X754	Intentional self-harm by explosive material
X755	Intentional self-harm by explosive material

X756	Intentional self-harm by explosive material
X757	Intentional self-harm by explosive material
X758	Intentional self-harm by explosive material
X759	Intentional self-harm by explosive material
X76	Intentional self-harm by smoke fire and flames
X760	Intentional self-harm by smoke fire and flames
X761	Intentional self-harm by smoke fire and flames
X762	Intentional self-harm by smoke fire and flames
X763	Intentional self-harm by smoke fire and flames
X764	Intentional self-harm by smoke fire and flames
X765	Intentional self-harm by smoke fire and flames
X766	Intentional self-harm by smoke fire and flames
X767	Intentional self-harm by smoke fire and flames
X768	Intentional self-harm by smoke fire and flames
X769	Intentional self-harm by smoke fire and flames
X77	Intentional self-harm by steam hot vapours and hot objects
X770	Intentional self-harm by steam hot vapours and hot objects
X771	Intentional self-harm by steam hot vapours and hot objects
X772	Intentional self-harm by steam hot vapours and hot objects
X773	Intentional self-harm by steam hot vapours and hot objects
X774	Intentional self-harm by steam hot vapours and hot objects
X775	Intentional self-harm by steam hot vapours and hot objects
X776	Intentional self-harm by steam hot vapours and hot objects
X777	Intentional self-harm by steam hot vapours and hot objects
X778	Intentional self-harm by steam hot vapours and hot objects
X779	Intentional self-harm by steam hot vapours and hot objects
X78	Intentional self-harm by sharp object
X780	Intentional self-harm by sharp object
X781	Intentional self-harm by sharp object
X782	Intentional self-harm by sharp object
X783	Intentional self-harm by sharp object
X784	Intentional self-harm by sharp object
X785	Intentional self-harm by sharp object
X786	Intentional self-harm by sharp object
X787	Intentional self-harm by sharp object
X788	Intentional self-harm by sharp object
X789	Intentional self-harm by sharp object
X79	Intentional self-harm by blunt object
X790	Intentional self-harm by blunt object
X791	Intentional self-harm by blunt object
X792	Intentional self-harm by blunt object
X793	Intentional self-harm by blunt object
X794	Intentional self-harm by blunt object
X795	Intentional self-harm by blunt object

X796	Intentional self-harm by blunt object
X797	Intentional self-harm by blunt object
X798	Intentional self-harm by blunt object
X799	Intentional self-harm by blunt object
X80	Intentional self-harm by jumping from a high place
X800	Intentional self-harm by jumping from a high place
X801	Intentional self-harm by jumping from a high place
X802	Intentional self-harm by jumping from a high place
X803	Intentional self-harm by jumping from a high place
X804	Intentional self-harm by jumping from a high place
X805	Intentional self-harm by jumping from a high place
X806	Intentional self-harm by jumping from a high place
X807	Intentional self-harm by jumping from a high place
X808	Intentional self-harm by jumping from a high place
X809	Intentional self-harm by jumping from a high place
X81	Intent self-harm by jumping or lying before moving object
X810	Intent self-harm by jumping or lying before moving object
X811	Intent self-harm by jumping or lying before moving object
X812	Intent self-harm by jumping or lying before moving object
X813	Intent self-harm by jumping or lying before moving object
X814	Intent self-harm by jumping or lying before moving object
X815	Intent self-harm by jumping or lying before moving object
X816	Intent self-harm by jumping or lying before moving object
X817	Intent self-harm by jumping or lying before moving object
X818	Intent self-harm by jumping or lying before moving object
X819	Intent self-harm by jumping or lying before moving object
X82	Intentional self-harm by crashing of motor vehicle
X820	Intentional self-harm by crashing of motor vehicle
X821	Intentional self-harm by crashing of motor vehicle
X822	Intentional self-harm by crashing of motor vehicle
X823	Intentional self-harm by crashing of motor vehicle
X824	Intentional self-harm by crashing of motor vehicle
X825	Intentional self-harm by crashing of motor vehicle
X826	Intentional self-harm by crashing of motor vehicle
X827	Intentional self-harm by crashing of motor vehicle
X828	Intentional self-harm by crashing of motor vehicle
X829	Intentional self-harm by crashing of motor vehicle
X83	Intentional self-harm by other specified means
X830	Intentional self-harm by other specified means
X831	Intentional self-harm by other specified means
X832	Intentional self-harm by other specified means
X833	Intentional self-harm by other specified means
X834	Intentional self-harm by other specified means
X835	Intentional self-harm by other specified means

X836	Intentional self-harm by other specified means
X837	Intentional self-harm by other specified means
X838	Intentional self-harm by other specified means
X839	Intentional self-harm by other specified means
X84	Intentional self-harm by unspecified means
X840	Intentional self-harm by unspecified means
X841	Intentional self-harm by unspecified means
X842	Intentional self-harm by unspecified means
X843	Intentional self-harm by unspecified means
X844	Intentional self-harm by unspecified means
X845	Intentional self-harm by unspecified means
X846	Intentional self-harm by unspecified means
X847	Intentional self-harm by unspecified means
X848	Intentional self-harm by unspecified means
X849	Intentional self-harm by unspecified means
Y10	Pois/expos nonopiod analges antipy & antirheum undet intent
Y100	Pois/expos nonopiod analges antipy & antirheum undet intent
Y101	Pois/expos nonopiod analges antipy & antirheum undet intent
Y102	Pois/expos nonopiod analges antipy & antirheum undet intent
Y103	Pois/expos nonopiod analges antipy & antirheum undet intent
Y104	Pois/expos nonopiod analges antipy & antirheum undet intent
Y105	Pois/expos nonopiod analges antipy & antirheum undet intent
Y106	Pois/expos nonopiod analges antipy & antirheum undet intent
Y107	Pois/expos nonopiod analges antipy & antirheum undet intent
Y108	Pois/expos nonopiod analges antipy & antirheum undet intent
Y109	Pois/expos nonopiod analges antipy & antirheum undet intent
Y11	Pois/expos ant-epi sed-hyp ant-park/psy'trp dgs undet intent
Y110	Pois/expos ant-epi sed-hyp ant-park/psy'trp dgs undet intent
Y111	Pois/expos ant-epi sed-hyp ant-park/psy'trp dgs undet intent
Y112	Pois/expos ant-epi sed-hyp ant-park/psy'trp dgs undet intent
Y113	Pois/expos ant-epi sed-hyp ant-park/psy'trp dgs undet intent
Y114	Pois/expos ant-epi sed-hyp ant-park/psy'trp dgs undet intent
Y115	Pois/expos ant-epi sed-hyp ant-park/psy'trp dgs undet intent
Y116	Pois/expos ant-epi sed-hyp ant-park/psy'trp dgs undet intent
Y117	Pois/expos ant-epi sed-hyp ant-park/psy'trp dgs undet intent
Y118	Pois/expos ant-epi sed-hyp ant-park/psy'trp dgs undet intent
Y119	Pois/expos ant-epi sed-hyp ant-park/psy'trp dgs undet intent
Y12	Pois/expos narcotics & psy'dys'tics [halluc'ns] undet intent
Y120	Pois/expos narcotics & psy'dys'tics [halluc'ns] undet intent
Y121	Pois/expos narcotics & psy'dys'tics [halluc'ns] undet intent
Y122	Pois/expos narcotics & psy'dys'tics [halluc'ns] undet intent
Y123	Pois/expos narcotics & psy'dys'tics [halluc'ns] undet intent
Y124	Pois/expos narcotics & psy'dys'tics [halluc'ns] undet intent
Y125	Pois/expos narcotics & psy'dys'tics [halluc'ns] undet intent

Y126	Pois/expos narcotics & psy'dys'tics [halluc'ns] undet intent
Y127	Pois/expos narcotics & psy'dys'tics [halluc'ns] undet intent
Y128	Pois/expos narcotics & psy'dys'tics [halluc'ns] undet intent
Y129	Pois/expos narcotics & psy'dys'tics [halluc'ns] undet intent
Y13	Pois/expos oth drgs acting on autonom nerv syst undet intent
Y130	Pois/expos oth drgs acting on autonom nerv syst undet intent
Y131	Pois/expos oth drgs acting on autonom nerv syst undet intent
Y132	Pois/expos oth drgs acting on autonom nerv syst undet intent
Y133	Pois/expos oth drgs acting on autonom nerv syst undet intent
Y134	Pois/expos oth drgs acting on autonom nerv syst undet intent
Y135	Pois/expos oth drgs acting on autonom nerv syst undet intent
Y136	Pois/expos oth drgs acting on autonom nerv syst undet intent
Y137	Pois/expos oth drgs acting on autonom nerv syst undet intent
Y138	Pois/expos oth drgs acting on autonom nerv syst undet intent
Y139	Pois/expos oth drgs acting on autonom nerv syst undet intent
Y14	Pois/expos oth /unsp drugs medics/biol subs undet intent
Y140	Pois/expos oth /unsp drugs medics/biol subs undet intent
Y141	Pois/expos oth /unsp drugs medics/biol subs undet intent
Y142	Pois/expos oth /unsp drugs medics/biol subs undet intent
Y143	Pois/expos oth /unsp drugs medics/biol subs undet intent
Y144	Pois/expos oth /unsp drugs medics/biol subs undet intent
Y145	Pois/expos oth /unsp drugs medics/biol subs undet intent
Y146	Pois/expos oth /unsp drugs medics/biol subs undet intent
Y147	Pois/expos oth /unsp drugs medics/biol subs undet intent
Y148	Pois/expos oth /unsp drugs medics/biol subs undet intent
Y149	Pois/expos oth /unsp drugs medics/biol subs undet intent
Y15	Poisoning by and exposure to alcohol undetermined intent
Y150	Poisoning by and exposure to alcohol undetermined intent
Y151	Poisoning by and exposure to alcohol undetermined intent
Y152	Poisoning by and exposure to alcohol undetermined intent
Y153	Poisoning by and exposure to alcohol undetermined intent
Y154	Poisoning by and exposure to alcohol undetermined intent
Y155	Poisoning by and exposure to alcohol undetermined intent
Y156	Poisoning by and exposure to alcohol undetermined intent
Y157	Poisoning by and exposure to alcohol undetermined intent
Y158	Poisoning by and exposure to alcohol undetermined intent
Y159	Poisoning by and exposure to alcohol undetermined intent
Y16	Poison/expos org solvs halogen h'carb & vaps undet intent
Y160	Poison/expos org solvs halogen h'carb & vaps undet intent
Y161	Poison/expos org solvs halogen h'carb & vaps undet intent
Y162	Poison/expos org solvs halogen h'carb & vaps undet intent
Y163	Poison/expos org solvs halogen h'carb & vaps undet intent
Y164	Poison/expos org solvs halogen h'carb & vaps undet intent
Y165	Poison/expos org solvs halogen h'carb & vaps undet intent

Y166	Poison/expos org solvs halogen h'carb & vaps undet intent
Y167	Poison/expos org solvs halogen h'carb & vaps undet intent
Y168	Poison/expos org solvs halogen h'carb & vaps undet intent
Y169	Poison/expos org solvs halogen h'carb & vaps undet intent
Y17	Poison'g/expos to oth gases and vapours undetermined intent
Y170	Poison'g/expos to oth gases and vapours undetermined intent
Y171	Poison'g/expos to oth gases and vapours undetermined intent
Y172	Poison'g/expos to oth gases and vapours undetermined intent
Y173	Poison'g/expos to oth gases and vapours undetermined intent
Y174	Poison'g/expos to oth gases and vapours undetermined intent
Y175	Poison'g/expos to oth gases and vapours undetermined intent
Y176	Poison'g/expos to oth gases and vapours undetermined intent
Y177	Poison'g/expos to oth gases and vapours undetermined intent
Y178	Poison'g/expos to oth gases and vapours undetermined intent
Y179	Poison'g/expos to oth gases and vapours undetermined intent
Y18	Poisoning by and exposure to pesticides undetermined intent
Y180	Poisoning by and exposure to pesticides undetermined intent
Y181	Poisoning by and exposure to pesticides undetermined intent
Y182	Poisoning by and exposure to pesticides undetermined intent
Y183	Poisoning by and exposure to pesticides undetermined intent
Y184	Poisoning by and exposure to pesticides undetermined intent
Y185	Poisoning by and exposure to pesticides undetermined intent
Y186	Poisoning by and exposure to pesticides undetermined intent
Y187	Poisoning by and exposure to pesticides undetermined intent
Y188	Poisoning by and exposure to pesticides undetermined intent
Y189	Poisoning by and exposure to pesticides undetermined intent
Y19	Poison/expos oth/unsp chemicals & noxious subs undet intent
Y190	Poison/expos oth/unsp chemicals & noxious subs undet intent
Y191	Poison/expos oth/unsp chemicals & noxious subs undet intent
Y192	Poison/expos oth/unsp chemicals & noxious subs undet intent
Y193	Poison/expos oth/unsp chemicals & noxious subs undet intent
Y194	Poison/expos oth/unsp chemicals & noxious subs undet intent
Y195	Poison/expos oth/unsp chemicals & noxious subs undet intent
Y196	Poison/expos oth/unsp chemicals & noxious subs undet intent
Y197	Poison/expos oth/unsp chemicals & noxious subs undet intent
Y198	Poison/expos oth/unsp chemicals & noxious subs undet intent
Y199	Poison/expos oth/unsp chemicals & noxious subs undet intent
Y20	Hanging strangulation and suffocation undetermined intent
Y200	Hanging strangulation and suffocation undetermined intent
Y201	Hanging strangulation and suffocation undetermined intent
Y202	Hanging strangulation and suffocation undetermined intent
Y203	Hanging strangulation and suffocation undetermined intent
Y204	Hanging strangulation and suffocation undetermined intent
Y205	Hanging strangulation and suffocation undetermined intent

Y206	Hanging strangulation and suffocation undetermined intent
Y207	Hanging strangulation and suffocation undetermined intent
Y208	Hanging strangulation and suffocation undetermined intent
Y209	Hanging strangulation and suffocation undetermined intent
Y21	Drowning and submersion undetermined intent
Y210	Drowning and submersion undetermined intent
Y211	Drowning and submersion undetermined intent
Y212	Drowning and submersion undetermined intent
Y213	Drowning and submersion undetermined intent
Y214	Drowning and submersion undetermined intent
Y215	Drowning and submersion undetermined intent
Y216	Drowning and submersion undetermined intent
Y217	Drowning and submersion undetermined intent
Y218	Drowning and submersion undetermined intent
Y219	Drowning and submersion undetermined intent
Y22	Handgun discharge undetermined intent
Y220	Handgun discharge undetermined intent
Y221	Handgun discharge undetermined intent
Y222	Handgun discharge undetermined intent
Y223	Handgun discharge undetermined intent
Y224	Handgun discharge undetermined intent
Y225	Handgun discharge undetermined intent
Y226	Handgun discharge undetermined intent
Y227	Handgun discharge undetermined intent
Y228	Handgun discharge undetermined intent
Y229	Handgun discharge undetermined intent
Y23	Rifle shotgun & larger firearm discharge undetermined intent
Y230	Rifle shotgun & larger firearm discharge undetermined intent
Y231	Rifle shotgun & larger firearm discharge undetermined intent
Y232	Rifle shotgun & larger firearm discharge undetermined intent
Y233	Rifle shotgun & larger firearm discharge undetermined intent
Y234	Rifle shotgun & larger firearm discharge undetermined intent
Y235	Rifle shotgun & larger firearm discharge undetermined intent
Y236	Rifle shotgun & larger firearm discharge undetermined intent
Y237	Rifle shotgun & larger firearm discharge undetermined intent
Y238	Rifle shotgun & larger firearm discharge undetermined intent
Y239	Rifle shotgun & larger firearm discharge undetermined intent
Y24	Other and unspecified firearm discharge undetermined intent
Y240	Other and unspecified firearm discharge undetermined intent
Y241	Other and unspecified firearm discharge undetermined intent
Y242	Other and unspecified firearm discharge undetermined intent
Y243	Other and unspecified firearm discharge undetermined intent
Y244	Other and unspecified firearm discharge undetermined intent
Y245	Other and unspecified firearm discharge undetermined intent

Y246	Other and unspecified firearm discharge undetermined intent
Y247	Other and unspecified firearm discharge undetermined intent
Y248	Other and unspecified firearm discharge undetermined intent
Y249	Other and unspecified firearm discharge undetermined intent
Y25	Contact with explosive material undetermined intent
Y250	Contact with explosive material undetermined intent
Y251	Contact with explosive material undetermined intent
Y252	Contact with explosive material undetermined intent
Y253	Contact with explosive material undetermined intent
Y254	Contact with explosive material undetermined intent
Y255	Contact with explosive material undetermined intent
Y256	Contact with explosive material undetermined intent
Y257	Contact with explosive material undetermined intent
Y258	Contact with explosive material undetermined intent
Y259	Contact with explosive material undetermined intent
Y26	Exposure to smoke fire and flames undetermined intent
Y260	Exposure to smoke fire and flames undetermined intent
Y261	Exposure to smoke fire and flames undetermined intent
Y262	Exposure to smoke fire and flames undetermined intent
Y263	Exposure to smoke fire and flames undetermined intent
Y264	Exposure to smoke fire and flames undetermined intent
Y265	Exposure to smoke fire and flames undetermined intent
Y266	Exposure to smoke fire and flames undetermined intent
Y267	Exposure to smoke fire and flames undetermined intent
Y268	Exposure to smoke fire and flames undetermined intent
Y269	Exposure to smoke fire and flames undetermined intent
Y27	Contact with steam hot vapours/objects undetermined intent
Y270	Contact with steam hot vapours/objects undetermined intent
Y271	Contact with steam hot vapours/objects undetermined intent
Y272	Contact with steam hot vapours/objects undetermined intent
Y273	Contact with steam hot vapours/objects undetermined intent
Y274	Contact with steam hot vapours/objects undetermined intent
Y275	Contact with steam hot vapours/objects undetermined intent
Y276	Contact with steam hot vapours/objects undetermined intent
Y277	Contact with steam hot vapours/objects undetermined intent
Y278	Contact with steam hot vapours/objects undetermined intent
Y279	Contact with steam hot vapours/objects undetermined intent
Y28	Contact with sharp object undetermined intent
Y280	Contact with sharp object undetermined intent
Y281	Contact with sharp object undetermined intent
Y282	Contact with sharp object undetermined intent
Y283	Contact with sharp object undetermined intent
Y284	Contact with sharp object undetermined intent
Y285	Contact with sharp object undetermined intent

Y286	Contact with sharp object undetermined intent
Y287	Contact with sharp object undetermined intent
Y288	Contact with sharp object undetermined intent
Y289	Contact with sharp object undetermined intent
Y29	Contact with blunt object undetermined intent
Y290	Contact with blunt object undetermined intent
Y291	Contact with blunt object undetermined intent
Y292	Contact with blunt object undetermined intent
Y293	Contact with blunt object undetermined intent
Y294	Contact with blunt object undetermined intent
Y295	Contact with blunt object undetermined intent
Y296	Contact with blunt object undetermined intent
Y297	Contact with blunt object undetermined intent
Y298	Contact with blunt object undetermined intent
Y299	Contact with blunt object undetermined intent
Y30	Falling jumping/pushed from high place undetermined intent
Y300	Falling jumping/pushed from high place undetermined intent
Y301	Falling jumping/pushed from high place undetermined intent
Y302	Falling jumping/pushed from high place undetermined intent
Y303	Falling jumping/pushed from high place undetermined intent
Y304	Falling jumping/pushed from high place undetermined intent
Y305	Falling jumping/pushed from high place undetermined intent
Y306	Falling jumping/pushed from high place undetermined intent
Y307	Falling jumping/pushed from high place undetermined intent
Y308	Falling jumping/pushed from high place undetermined intent
Y309	Falling jumping/pushed from high place undetermined intent
Y31	Falling lying running before/into moving obj undet intent
Y310	Falling lying running before/into moving obj undet intent
Y311	Falling lying running before/into moving obj undet intent
Y312	Falling lying running before/into moving obj undet intent
Y313	Falling lying running before/into moving obj undet intent
Y314	Falling lying running before/into moving obj undet intent
Y315	Falling lying running before/into moving obj undet intent
Y316	Falling lying running before/into moving obj undet intent
Y317	Falling lying running before/into moving obj undet intent
Y318	Falling lying running before/into moving obj undet intent
Y319	Falling lying running before/into moving obj undet intent
Y32	Crashing of motor vehicle undetermined intent
Y320	Crashing of motor vehicle undetermined intent
Y321	Crashing of motor vehicle undetermined intent
Y322	Crashing of motor vehicle undetermined intent
Y323	Crashing of motor vehicle undetermined intent
Y324	Crashing of motor vehicle undetermined intent
Y325	Crashing of motor vehicle undetermined intent

Y326	Crashing of motor vehicle undetermined intent
Y327	Crashing of motor vehicle undetermined intent
Y328	Crashing of motor vehicle undetermined intent
Y329	Crashing of motor vehicle undetermined intent
Y33	Other specified events undetermined intent
Y330	Other specified events undetermined intent
Y331	Other specified events undetermined intent
Y332	Other specified events undetermined intent
Y333	Other specified events undetermined intent
Y334	Other specified events undetermined intent
Y335	Other specified events undetermined intent
Y336	Other specified events undetermined intent
Y337	Other specified events undetermined intent
Y338	Other specified events undetermined intent
Y339	Other specified events undetermined intent
Y34	Unspecified event undetermined intent
Y340	Unspecified event undetermined intent
Y341	Unspecified event undetermined intent
Y342	Unspecified event undetermined intent
Y343	Unspecified event undetermined intent
Y344	Unspecified event undetermined intent
Y345	Unspecified event undetermined intent
Y346	Unspecified event undetermined intent
Y347	Unspecified event undetermined intent
Y348	Unspecified event undetermined intent
Y349	Unspecified event undetermined intent
Y870	Sequelae of intentional self-harm
Y872	Sequelae of events of undetermined intent
Z915	Personal history of self-harm

Table 4: Self harm related READ codes

READ codes	Descriptions
14K1.	Intentional overdose of prescription only medication
SL...	Poisoning
SL90.	Antidepressant poisoning
SL900	Amitriptyline poisoning
SL901	Imipramine poisoning
SL902	Monoamine oxidase inhibitor poisoning
SL903	Trazodone poisoning
SL90z	Anti-depressant poisoning NOS
TK...	Suicide and selfinflicted injury
TK0..	Suicide + selfinflicted poisoning by solid/liquid substances
TK01.	Suicide + selfinflicted poisoning by barbiturates

TK010	Suicide and self inflicted injury by Amylobarbitone
TK011	Suicide and self inflicted injury by Barbitone
TK014	Suicide and self inflicted injury by Phenobarbitone
TK02.	Suicide + selfinflicted poisoning by oth sedatives/hypnotics
TK03.	Suicide + selfinflicted poisoning tranquilliser/psychotropic
TK04.	Suicide + selfinflicted poisoning by other drugs/medicines
TK05.	Suicide + selfinflicted poisoning by drug or medicine NOS
TK06.	Suicide + selfinflicted poisoning by agricultural chemical
TK07.	Suicide + selfinflicted poisoning by corrosive/caustic subst
TK0z.	Suicide + selfinflicted poisoning by solid/liquid subst NOS
TK1..	Suicide + selfinflicted poisoning by gases in domestic use
TK10.	Suicide + selfinflicted poisoning by gas via pipeline
TK11.	Suicide + selfinflicted poisoning by liquified petrol gas
TK1y.	Suicide and selfinflicted poisoning by other utility gas
TK1z.	Suicide + selfinflicted poisoning by domestic gases NOS
TK2..	Suicide + selfinflicted poisoning by other gases and vapours
TK20.	Suicide + selfinflicted poisoning by motor veh exhaust gas
TK21.	Suicide and selfinflicted poisoning by other carbon monoxide
TK2z.	Suicide + selfinflicted poisoning by gases and vapours NOS
TK3..	Suicide + selfinflicted injury by hang/strangulate/suffocate
TK30.	Suicide and selfinflicted injury by hanging
TK31.	Suicide + selfinflicted injury by suffocation by plastic bag
TK3y.	Suicide + selfinflicted inj oth mean hang/strangle/suffocate
TK3z.	Suicide + selfinflicted inj by hang/strangle/suffocate NOS
TK4..	Suicide and selfinflicted injury by drowning
TK5..	Suicide and selfinflicted injury by firearms and explosives
TK51.	Suicide and selfinflicted injury by shotgun
TK52.	Suicide and selfinflicted injury by hunting rifle
TK53.	Suicide and selfinflicted injury by military firearms
TK54.	Suicide and selfinflicted injury by other firearm
TK5z.	Suicide and selfinflicted injury by firearms/explosives NOS
TK6..	Suicide and selfinflicted injury by cutting and stabbing
TK60.	Suicide and selfinflicted injury by cutting
TK601	Self inflicted lacerations to wrist
TK61.	Suicide and selfinflicted injury by stabbing
TK6z.	Suicide and selfinflicted injury by cutting and stabbing NOS
TK7..	Suicide and selfinflicted injury by jumping from high place
TK70.	Suicide+selfinflicted injury-jump from residential premises
TK71.	Suicide+selfinflicted injury-jump from oth manmade structure
TK72.	Suicide+selfinflicted injury-jump from natural sites
TK7z.	Suicide+selfinflicted injury-jump from high place NOS
TKx..	Suicide and selfinflicted injury by other means
TKx0.	Suicide + selfinflicted injury-jump/lie before moving object
TKx00	Suicide + selfinflicted injury-jumping before moving object

TKx1.	Suicide and selfinflicted injury by burns or fire
TKx2.	Suicide and selfinflicted injury by scald
TKx3.	Suicide and selfinflicted injury by extremes of cold
TKx4.	Suicide and selfinflicted injury by electrocution
TKx5.	Suicide and selfinflicted injury by crashing motor vehicle
TKx6.	Suicide and selfinflicted injury by crashing of aircraft
TKx7.	Suicide and selfinflicted injury caustic subst, excl poison
TKxy.	Suicide and selfinflicted injury by other specified means
TKxz.	Suicide and selfinflicted injury by other means NOS
TKz..	Suicide and selfinflicted injury NOS
U2...	[X]Intentional self-harm
U20..	[X]Intentional self poisoning/exposure to noxious substances
U200.	[X]Intent self poison/exposure to nonopioid analgesic
U2000	[X]Int self poison/exposure to nonopioid analgesic at home
U2001	[X]Intent self poison nonopioid analgesic at res institut
U2002	[X]Int self poison nonopioid analges school/pub admin area
U2003	[X]Int self poison nonopioid analges in sport/athletic area
U2004	[X]Intent self pois nonopioid analgesic in street/highway
U2005	[X]Intent self pois nonopioid analgesic trade/service area
U2006	[X]Int self pois nonopioid analgesic indust/construct area
U2007	[X]Int self poison/exposure to nonopioid analgesic on farm
U200y	[X]Int self poison nonopioid analgesic other spec place
U200z	[X]Intent self poison nonopioid analgesic unspecif place
U201.	[X]Intent self poison/exposure to antiepileptic
U2010	[X]Int self poison/exposure to antiepileptic at home
U2011	[X]Intent self poison antiepileptic at res institut
U2012	[X]Intent self pois nonopioid analges school/pub admin area
U2013	[X]Int self poison antiepileptic in sport/athletic area
U2014	[X]Intent self pois antiepileptic in street/highway
U2015	[X]Intent self pois antiepileptic trade/service area
U2016	[X]Int self poison antiepileptic indust/construct area
U2017	[X]Int self poison/exposure to antiepileptic on farm
U201y	[X]Intent self poison antiepileptic other spec place
U201z	[X]Intent self poison antiepileptic unspecif place
U202.	[X]Intent self poison/exposure to sedative hypnotic
U2020	[X]Int self poison/exposure to sedative hypnotic at home
U2021	[X]Intent self poison sedative hypnotic at res institut
U2022	[X]Int self poison sedative hypnotic school/pub admin area
U2023	[X]Int self poison sedative hypnotic in sport/athletic area
U2024	[X]Intent self pois sedative hypnotic in street/highway
U2025	[X]Intent self pois sedative hypnotic trade/service area
U2026	[X]Int self pois sedative hypnotic indust/construct area
U2027	[X]Int self poison/exposure to sedative hypnotic on farm
U202y	[X]Int self poison sedative hypnotic other spec place

U202z	[X]Intent self poison sedative hypnotic unspecif place
U203.	[X]Intent self poison/exposure to antiparkinson drug
U2030	[X]Int self poison/exposure to antiparkinson drug at home
U2031	[X]Intent self poison antiparkinson drug at res institut
U2032	[X]Int self poison antparkinson drug school/pub admin area
U2033	[X]Int self poison antparkinson drug in sport/athletic area
U2034	[X]Intent self pois antiparkinson drug in street/highway
U2035	[X]Intent self pois antiparkinson drug trade/service area
U2036	[X]Int self pois antiparkinson drug indust/construct area
U2037	[X]Int self poison/exposure to antiparkinson drug on farm
U203y	[X]Int self poison antiparkinson drug other spec place
U203z	[X]Intent self poison antiparkinson drug unspecif place
U204.	[X]Intent self poison/exposure to psychotropic drug
U2040	[X]Int self poison/exposure to psychotropic drug at home
U2041	[X]Intent self poison psychotropic drug at res institut
U2042	[X]Int self poison psychotropic drug school/pub admin area
U2043	[X]Int self poison psychotropic drug in sport/athletic area
U2044	[X]Intent self pois psychotropic drug in street/highway
U2045	[X]Intent self pois psychotropic drug trade/service area
U2046	[X]Int self pois psychotropic drug indust/construct area
U2047	[X]Int self poison/exposure to psychotropic drug on farm
U204y	[X]Int self poison psychotropic drug other spec place
U204z	[X]Intent self poison psychotropic drug unspecif place
U205.	[X]Intent self poison/exposure to narcotic drug
U2050	[X]Int self poison/exposure to narcotic drug at home
U2051	[X]Intent self poison narcotic drug at res institut
U2052	[X]Int self poison narcotic drug school/pub admin area
U2053	[X]Int self poison narcotic drug in sport/athletic area
U2054	[X]Intent self pois narcotic drug in street/highway
U2055	[X]Intent self pois narcotic drug trade/service area
U2056	[X]Int self pois narcotic drug indust/construct area
U2057	[X]Int self poison/exposure to narcotic drug on farm
U205y	[X]Int self poison narcotic drug other spec place
U205z	[X]Intent self poison narcotic drug unspecif place
U206.	[X]Intent self poison/exposure to hallucinogen
U2060	[X]Int self poison/exposure to hallucinogen at home
U2061	[X]Intent self poison hallucinogen at res institut
U2062	[X]Int self poison hallucinogenschool/pub admin area
U2063	[X]Int self poison hallucinogenin sport/athletic area
U2064	[X]Intent self pois hallucinogen in street/highway
U2065	[X]Intent self pois hallucinogen trade/service area
U2066	[X]Int self pois hallucinogen indust/construct area
U2067	[X]Int self poison/exposure to hallucinogen on farm
U206y	[X]Int self poison hallucinogen other spec place

U206z	[X]Intent self poison hallucinogen unspecif place
U207.	[X]Intent self poison/exposure to oth autonomic drug
U2070	[X]Int self poison/exposure to oth autonomic drug at home
U2071	[X]Intent self poison oth autonomic drug at res institut
U2072	[X]Int self poison oth autonom drug school/pub admin area
U2073	[X]Int self poison oth autonom drug in sport/athletic area
U2074	[X]Intent self pois oth autonomic drug in street/highway
U2075	[X]Intent self pois oth autonomic drug trade/service area
U2076	[X]Int self pois oth autonomic drug indust/construct area
U2077	[X]Int self poison/exposure to oth autonomic drug on farm
U207y	[X]Int self poison oth autonomic drug other spec place
U207z	[X]Intent self poison oth autonomic drug unspecif place
U208.	[X]Int self poison/exposure to other/unspec drug/medicament
U2080	[X]Int self poison/exposure to oth/unsp drug/medicam home
U2081	[X]Intent self poison oth/unsp drug/medicam res institut
U2082	[X]Int self poison oth/uns drug/med school/pub admin area
U2083	[X]Int self poison oth/uns drug/med in sport/athletic area
U2084	[X]Intent self pois oth/unsp drug/medic in street/highway
U2085	[X]Intent self pois oth/unsp drug/medic trade/service area
U2086	[X]Int self pois oth/unsp drug/medic indust/construct area
U2087	[X]Int self poison/exposure to oth/unsp drug/medic on farm
U208y	[X]Int self poison oth/unsp drug/medic other spec place
U208z	[X]Intent self poison oth/unsp drug/medic unspecif place
U20A.	[X]Intentional self poison organ solvent,halogen hydrocarb
U20A0	[X]Intent self pois organ solvent,halogen hydrocarb, home
U20A1	[X]Int self poison org solvent,halogen hydrocarb,res instit
U20A2	[X]Int self poison org solvent,halogen hydrocarb, school
U20A3	[X]Int self poison org solvent,halogen hydrocarb,sport area
U20A4	[X]Int self poison org solvent,halogen hydrocarb,in highway
U20A5	[X]Int self poison org solvent,halogen hydrocarb,trade area
U20A6	[X]Int self pois org solvent,halogen hydrocarb,indust area
U20A7	[X]Int self poison org solvent,halogen hydrocarb,on farm
U20Ay	[X]Int self pois org solv,halogen hydrocarb,oth spec place
U20Az	[X]Int self pois org solv,halogen hydrocarb, unspec place
U20B.	[X]Intent self poison/exposure to other gas/vapour
U20B0	[X]Int self poison/exposure to other gas/vapour at home
U20B1	[X]Intent self poison other gas/vapour at res institut
U20B2	[X]Int self poison other gas/vapour school/pub admin area
U20B3	[X]Int self poison other gas/vapour in sport/athletic area
U20B4	[X]Intent self pois other gas/vapour in street/highway
U20B5	[X]Intent self pois other gas/vapour trade/service area
U20B6	[X]Int self pois other gas/vapour indust/construct area
U20B7	[X]Int self poison/exposure to other gas/vapour on farm
U20By	[X]Int self poison other gas/vapour other spec place

U20Bz	[X]Intent self poison other gas/vapour unspecif place
U20C.	[X]Intent self poison/exposure to pesticide
U20C0	[X]Int self poison/exposure to pesticide at home
U20C1	[X]Intent self poison pesticide at res institut
U20C2	[X]Int self poison pesticide school/pub admin area
U20C3	[X]Int self poison pesticide in sport/athletic area
U20C4	[X]Intent self pois pesticide in street/highway
U20C5	[X]Intent self pois pesticide trade/service area
U20C6	[X]Int self pois pesticide indust/construct area
U20C7	[X]Int self poison/exposure to pesticide on farm
U20Cy	[X]Int self poison pesticide other spec place
U20Cz	[X]Intent self poison pesticide unspecif place
U20y.	[X]Intent self poison/exposure to unspecif chemical
U20y0	[X]Int self poison/exposure to unspecif chemical at home
U20y1	[X]Intent self poison unspecif chemical at res institut
U20y2	[X]Int self poison unspecif chemical school/pub admin area
U20y3	[X]Int self poison unspecif chemical in sport/athletic area
U20y4	[X]Intent self pois unspecif chemical in street/highway
U20y5	[X]Intent self pois unspecif chemical trade/service area
U20y6	[X]Int self pois unspecif chemical indust/construct area
U20y7	[X]Int self poison/exposure to unspecif chemical on farm
U20yy	[X]Int self poison unspecif chemical other spec place
U20yz	[X]Intent self poison unspecif chemical unspecif place
U21..	[X]Intent self harm by hanging strangulation / suffocation
U210.	[X]Intent self harm by hanging strangulat/suffocat occ home
U211.	[X]Intent self harm by hangng strangult/suffoct resid instit
U212.	[X]Inten slf harm hang strang/suffc sch oth ins/pub adm area
U213.	[X]Intent self harm by hang strangl/suffc sport/athlet area
U214.	[X]Intent self harm by hangng strangult/suffoct street/h'way
U215.	[X]Intent self harm by hang strangl/suffc trade/service area
U216.	[X]Intent self harm by hang strangl/suffc indust/constr area
U217.	[X]Intent self harm by hanging strangulat/suffocat occ farm
U21y.	[X]Intent self harm by hangng strangul/suffoct oth spec plce
U21z.	[X]Intent self harm by hangng strangul/suffoct unspecif plce
U22..	[X]Intentional self harm by drowning and submersion
U220.	[X]Intent self harm by drowning/submersion occurrn at home
U221.	[X]Intent self harm by drowning/submersn occ resid instit'n
U222.	[X]Intent self harm drown/submers occ sch/ins/pub adm area
U223.	[X]Intent self harm by drown/submersn occ sport/athlet area
U224.	[X]Intent self harm by drowning/submersn occ street/highway
U225.	[X]Intent self harm by drown/submersn occ trade/servce area
U226.	[X]Intent self harm by drown/submers occ indust/constr area
U227.	[X]Intent self harm by drowning/submersion occurrn on farm
U22y.	[X]Intent self harm by drown/submersn occ oth specif place

U22z.	[X]Intent self harm by drown/submersn occ unspecified place
U23..	[X]Intentional self harm by handgun discharge
U230.	[X]Intention self harm by handgun discharge occurrn at home
U231.	[X]Intent self harm by handgun disch occ in resid instit'n
U232.	[X]Intent self harm h'gun disch occ sch oth ins/pub adm area
U233.	[X]Intent self harm by handgun disch occ sport/athlet area
U234.	[X]Intent self harm by handgun disch occ on street/highway
U235.	[X]Intent self harm by handgun disch occ trade/service area
U236.	[X]Intent self harm by handgun disch occ indust/constr area
U237.	[X]Intention self harm by handgun discharge occurrn on farm
U23y.	[X]Intent self harm by handgun disch occ at oth specif plce
U23z.	[X]Intent self harm by handgun disch occ at unspecif place
U24..	[X]Intent self harm by rifle shotgun/larger firearm disch
U240.	[X]Intent self harm rifle sh'gun/largr firarm disch occ home
U241.	[X]Int self harm rifl s'gun/lrg frarm disch occ resid instit
U242.	[X]Int slf hrm rifl s'gun/lrg frarm dis sch/ins/pub adm area
U243.	[X]Int self harm rifl s'gun/lrg frarm disch sprt/athlet area
U244.	[X]Int self harm rifl s'gun/lrg frarm disch occ street/h'way
U245.	[X]Int self harm rifl s'gun/lrg frarm disch trad/servce area
U246.	[X]Int slf hrm rifl s'gun/lrg frarm disch indust/constr area
U247.	[X]Intent self harm rifle sh'gun/largr firarm disch occ farm
U24y.	[X]Int self harm rifl s'gun/lrg frarm disch oth specif place
U24z.	[X]Int self harm rifl s'gun/lrg frarm disch occ unspec place
U25..	[X]Intent self harm by other/unspecified firearm discharge
U250.	[X]Intent self harm oth/unspecif firearm disch occ at home
U251.	[X]Intent self harm oth/unsp firearm disch occ resid instit
U252.	[X]Inten self harm oth/uns firarm disch sch/ins/pub adm area
U253.	[X]Inten self harm oth/uns firearm disch occ sprt/athl area
U254.	[X]Intent self harm oth/unsp firearm disch occ street/h'way
U255.	[X]Intent self harm oth/uns firearm disch trade/servce area
U256.	[X]Inten self harm oth/uns firearm disch indust/constr area
U257.	[X]Intent self harm oth/unspecif firearm disch occ on farm
U25y.	[X]Intent self harm oth/unsp firearm disch oth specif place
U25z.	[X]Intent self harm oth/unsp firearm disch occ unspecif plce
U26..	[X]Intentional self harm by explosive material
U260.	[X]Intention self harm by explosive material occurrn home
U261.	[X]Intention self harm by explosiv materl occ resid instit
U262.	[X]Intent self harm by explosiv materl sch/ins/pub adm area
U263.	[X]Intent self harm by explosv materl occ sport/athlet area
U264.	[X]Intention self harm by explosiv materl occ street/highway
U265.	[X]Intent self harm by explosv materl occ trade/servce area
U266.	[X]Intent self harm by explosv materl occ indust/constr area
U267.	[X]Intention self harm by explosive material occurrn farm
U26y.	[X]Intent self harm by explosiv materl occ oth specif place

U26z.	[X]Intent self harm by explosiv materl occ unspecif place
U27..	[X]Intentional self harm by smoke, fire and flames
U270.	[X]Intention self harm by smoke fire/flames occurrn at home
U271.	[X]Intent self harm by smoke fire/flame occ resid instit'n
U272.	[X]Intent self harm by smoke fire/flame sch/ins/pub adm area
U273.	[X]Intent self harm by smok fire/flam occ sport/athlet area
U274.	[X]Intent self harm by smoke fire/flame occ street/highway
U275.	[X]Intent self harm by smok fire/flam occ trade/servce area
U276.	[X]Intent self harm by smok fire/flam occ indust/constr area
U277.	[X]Intention self harm by smoke fire/flames occurrn on farm
U27y.	[X]Intent self harm by smoke fire/flame occ oth specif plce
U27z.	[X]Intent self harm by smoke fire/flames occ unspecif place
U28..	[X]Intentional self harm by steam hot vapours / hot objects
U280.	[X]Intent self harm by steam hot vapour/hot obj occ at home
U281.	[X]Intent self harm by steam hot vapour/obj occ resid instit
U282.	[X]Int self harm by steam hot vapor/obj sch/ins/pub adm area
U283.	[X]Int self harm by steam hot vapour/obj occ sport/athl area
U284.	[X]Intent self harm by steam hot vapour/obj occ street/h'way
U285.	[X]Int self harm by steam hot vapour/obj trade/service area
U286.	[X]Int self harm by steam hot vapour/obj indust/constr area
U287.	[X]Intent self harm by steam hot vapour/hot obj occ on farm
U28y.	[X]Intent self harm by steam hot vapour/obj oth specif place
U28z.	[X]Intent self harm by steam hot vapour/obj occ unspec place
U29..	[X]Intentional self harm by sharp object
U290.	[X]Intentional self harm by sharp object occurrence at home
U291.	[X]Intent self harm by sharp object occ resident instit'n
U292.	[X]Intent self harm sharp obj occ sch oth ins/pub adm area
U293.	[X]Intent self harm by sharp object occ sports/athlet area
U294.	[X]Intention self harm by sharp object occ street/highway
U295.	[X]Intent self harm by sharp object occ trade/service area
U296.	[X]Intent self harm by sharp object occ indust/constr area
U297.	[X]Intentional self harm by sharp object occurrence on farm
U29y.	[X]Intention self harm by sharp object occ oth specif place
U29z.	[X]Intentional self harm by sharp object occ unspecif place
U2A..	[X]Intentional self harm by blunt object
U2A0.	[X]Intentional self harm by blunt object occurrence at home
U2A1.	[X]Intent self harm by blunt object occ resident instit'n
U2A2.	[X]Intent self harm blunt obj occ sch oth ins/pub adm area
U2A3.	[X]Intent self harm by blunt object occ sports/athlet area
U2A4.	[X]Intention self harm by blunt object occ street/highway
U2A5.	[X]Intent self harm by blunt object occ trade/service area
U2A6.	[X]Intent self harm by blunt object occ indust/constr area
U2A7.	[X]Intentional self harm by blunt object occurrence on farm
U2Ay.	[X]Intention self harm by blunt object occ oth specif place

U2Az.	[X]Intentional self harm by blunt object occ unspecif place
U2B..	[X]Intentional self harm by jumping from a high place
U2B0.	[X]Intent self harm by jumping from high place occ at home
U2B1.	[X]Intent self harm by jump from high place occ resid instit
U2B2.	[X]Int self harm jump fr high place sch oth ins/pub adm area
U2B3.	[X]Intent self harm by jump from high place sport/athl area
U2B4.	[X]Intent self harm by jump from high place occ street/h'way
U2B5.	[X]Intent self harm by jump from high place trad/servce area
U2B6.	[X]Int self harm by jump from high place indust/constr area
U2B7.	[X]Intent self harm by jumping from high place occ on farm
U2By.	[X]Int self harm by jump from high place occ oth specif plce
U2Bz.	[X]Int self harm by jump from high place occ unspecif place
U2C..	[X]Intent self harm by jumping / lying before moving object
U2C0.	[X]Intent self harm by jump/lying befor moving obj occ home
U2C1.	[X]Int self harm jump/lying befr mov obje occ resid instit'n
U2C2.	[X]Int self harm jump/lying bef mov obj sch/ins/pub adm area
U2C3.	[X]Int self harm jump/lying bef mov obj occ sprt/athlet area
U2C4.	[X]Int self harm jump/lying befr mov obje occ street/highway
U2C5.	[X]Int self harm jump/lying bef mov obj occ trad/servce area
U2C6.	[X]Int self harm jump/lying befr mov obj indust/constr area
U2C7.	[X]Intent self harm by jump/lying befor moving obj occ farm
U2Cy.	[X]Int self harm jump/lying bef mov obje occ oth specif plce
U2Cz.	[X]Int self harm jump/lying bef mov obje occ unspecif place
U2D..	[X]Intentional self harm by crashing of motor vehicle
U2D0.	[X]Intent self harm by crash of motor vehicl occurrn at home
U2D1.	[X]Intent self harm by crash motor vehicl occ resid instit'n
U2D2.	[X]Int self harm crash motor vehicl occ sch/ins/pub adm area
U2D3.	[X]Intent self harm by crash motor vehicl occ sprt/athl area
U2D4.	[X]Intent self harm by crash motor vehicl occ street/highway
U2D5.	[X]Intent self harm crash motor vehicl occ trade/servce area
U2D6.	[X]Intent self harm crash motor vehic occ indust/constr area
U2D7.	[X]Intent self harm by crash of motor vehicl occurrn on farm
U2Dy.	[X]Intent self harm by crash motor vehic occ oth specif plce
U2Dz.	[X]Intent self harm by crash motor vehic occ unspecif place
U2E..	[X]Self mutilation
U2y..	[X]Intentional self harm by other specified means
U2y0.	[X]Intentionl self harm by oth specif means occurrn at home
U2y1.	[X]Intent self harm by oth specif means occ resid instit'n
U2y2.	[X]Intent self harm oth specif mean occ sch/ins/pub adm area
U2y3.	[X]Intent self harm by oth specif means occ sport/athl area
U2y4.	[X]Intent self harm by oth specif means occ street/highway
U2y5.	[X]Intent self harm by oth specif means occ trad/servce area
U2y6.	[X]Intent self harm oth specif means occ indust/constr area
U2y7.	[X]Intentionl self harm by oth specif means occurrn on farm

U2yy.	[X]Intent self harm oth specif means occ oth specif place
U2yz.	[X]Intent self harm by oth specif means occ unspecif place
U2z..	[X]Intentional self harm by unspecified means
U2z0.	[X]Intentional self harm by unspecif means occurrn at home
U2z1.	[X]Intent self harm by unspecif means occurrn resid instit'n
U2z2.	[X]Intent self harm by unspec mean occ sch/ins/pub adm area
U2z3.	[X]Intent self harm unspecif means occurrn sport/athlet area
U2z4.	[X]Intent self harm by unspecif means occurrn street/highway
U2z5.	[X]Intent self harm unspecif means occurrn trade/servce area
U2z6.	[X]Intent self harm unspecif mean occurrn indust/constr area
U2z7.	[X]Intentional self harm by unspecif means occurrn on farm
U2zy.	[X]Intent self harm by unspecif means occ oth specif place
U2zz.	[X]Intent self harm by unspecif means occ at unspecif place
U41..	[X]Hanging strangulation + suffocation undetermined intent
U44..	[X]Rifle shotgun+larger firearm discharge undetermin intent
U45..	[X]Other+unspecified firearm discharge undetermined intent
U4B..	[X]Falling jumping/pushed from high place undeterm intent
U4Bz.	[X]Fall jump/push frm high plce undt intnt occ unspecif plce
U72..	[X]Sequel intentn self-harm assault+event of undeterm intent
U720.	[X]Sequelae of intentional self-harm
ZRLfC12	Health of the Nation Outcome Scales item 2– nonaccidental self-injury
ZX...	Self-harm
ZX1..	Self-injurious behaviour
ZX11.	Biting self
ZX12.	Burning self
ZX13.	Cutting self
ZX15.	Drowning self
ZX18.	Hanging self
ZX19.	Hitting self
ZX191	Punching self
ZX192	Slapping self
ZX1B.	Jumping from height
ZX1B1	Jumping from building
ZX1B2	Jumping from bridge
ZX1B3	Jumping from cliff
ZX1C.	Nipping self
ZX1E.	Pinching self
ZX1G.	Scratches self
ZX1H.	Self-asphyxiation
ZX1H1	Self-strangulation
ZX1H2	Self-suffocation
ZX1I.	Self-scalding
ZX1J.	Self-electrocution
ZX1K.	Self-incineration

ZX1L.	Self-mutilation
ZX1L1	Self-mutilation of hands
ZX1L2	Self-mutilation of genitalia
ZX1L3	Self-mutilation of penis
ZX1L6	Self-mutilation of ears
ZX1LD	[X]Self mutilation
ZX1M.	Shooting self
ZX1N.	Stabbing self
ZX1Q.	Throwing self in front of train
ZX1R.	Throwing self in front of vehicle
ZX1S.	Throwing self onto floor

Table 5: Depression related ICD10 codes

**ICD10 codes**	**Descriptions**
F251	Schizoaffective disorder depressive type
F32	Depressive episode
F320	Mild depressive episode
F321	Moderate depressive episode
F322	Severe depressive episode without psychotic symptoms
F323	Severe depressive episode with psychotic symptoms
F328	Other depressive episodes
F329	Depressive episode, unspecified
F33	Recurrent depressive disorder
F330	Recurrent depressive disorder, current episode mild
F331	Recurrent depressive disorder, current episode moderate
F332	Recurrent depressive disorder, current episode severe without psychotic symptoms
F333	Recurrent depressive disorder, current episode severe with psychotic symptoms
F334	Recurrent depressive disorder, currently in remission
F338	Other recurrent depressive disorders
F339	Recurrent depressive disorder, unspecified
F341	Dysthymia

Table 6: Depression related READ codes

RAED codes	Descriptions
1B17.	Depressed
1B1U.	Symptoms of depression
1BQ..	Loss of capacity for enjoyment
1BT..	Depressed mood
1BU..	Loss of hope for the future
2257	O/E - depressed

E0013	Presenile dementia with depression
E0021	Senile dementia with depression
E112.	Single major depressive episode
E1120	Single major depressive episode, unspecified
E1121	Single major depressive episode, mild
E1122	Single major depressive episode, moderate
E1123	Single major depressive episode, severe, without psychosis
E1124	Single major depressive episode, severe, with psychosis
E1125	Single major depressive episode, partial or unspec remission
E1126	Single major depressive episode, in full remission
E112z	Single major depressive episode NOS
E113.	Recurrent major depressive episode
E1130	Recurrent major depressive episodes, unspecified
E1131	Recurrent major depressive episodes, mild
E1132	Recurrent major depressive episodes, moderate
E1133	Recurrent major depressive episodes, severe, no psychosis
E1134	Recurrent major depressive episodes, severe, with psychosis
E1135	Recurrent major depressive episodes,partial/unspec remission
E1136	Recurrent major depressive episodes, in full remission
E1137	Recurrent depression
E113z	Recurrent major depressive episode NOS
E118.	Seasonal affective disorder
E11y2	Atypical depressive disorder
E11z2	Masked depression
E130.	Reactive depressive psychosis
E135.	Agitated depression
E2003	Anxiety with depression
E204.	Neurotic depression reactive type
E291.	Prolonged depressive reaction
E2B..	Depressive disorder NEC
E2B0.	Postviral depression
E2B1.	Chronic depression
Eu204	[X]Post-schizophrenic depression
Eu251	[X]Schizoaffective disorder, depressive type
Eu32.	[X]Depressive episode
Eu320	[X]Mild depressive episode
Eu321	[X]Moderate depressive episode
Eu322	[X]Severe depressive episode without psychotic symptoms
Eu323	[X]Severe depressive episode with psychotic symptoms
Eu324	[X]Mild depression
Eu325	[X]Major depression, mild
Eu326	[X]Major depression, moderately severe
Eu327	[X]Major depression, severe without psychotic symptoms
Eu328	[X]Major depression, severe with psychotic symptoms

Eu32y	[X]Other depressive episodes
Eu32z	[X]Depressive episode, unspecified
Eu33.	[X]Recurrent depressive disorder
Eu330	[X]Recurrent depressive disorder, current episode mild
Eu331	[X]Recurrent depressive disorder, current episode moderate
Eu332	[X]Recurr depress disorder cur epi severe without psyc sympt
Eu333	[X]Recurrent depress disorder cur epi severe with psyc symp
Eu334	[X]Recurrent depressive disorder, currently in remission
Eu33y	[X]Other recurrent depressive disorders
Eu33z	[X]Recurrent depressive disorder, unspecified
Eu341	[X]Dysthymia
Eu412	[X]Mixed anxiety and depressive disorder

Table 7: Depression - medication READ codes

RAED codes	Descriptions
d11..	CHLORAL HYDRATE
d12..	CLOMETHIAZOLE EDISYLATE [HYPNOTIC]
d13..	*DICHLORALPHENAZONE
d14..	*FLUNITRAZEPAM
d15..	FLURAZEPAM
d16..	LOPRAZOLAM
d17..	LORMETAZEPAM
d18..	NITRAZEPAM
d1a..	TEMAZEPAM [HYPNOTIC]
d1b..	*TRIAZOLAM
d1c..	TRICLOFOS SODIUM
d1d..	ZOPICLONE
d1f..	ZOLPIDEM
d1g..	ZALEPLON
d1h..	MELATONIN
d21..	DIAZEPAM [ANXIOLYTIC]
d22..	ALPRAZOLAM
d23..	BROMAZEPAM
d24..	CHLORDIAZEPOXIDE
d25..	CHLORMEZANONE
d26..	CLOBAZAM
d27..	CLORAZEPATE DIPOTASSIUM
d28..	HYDROXYZINE HCL [ANXIOLYTIC]
d29..	*KETAZOLAM
d2a..	LORAZEPAM [ANXIOLYTIC]
d2b..	*MEDAZEPAM
d2c..	MEPROBAMATE
d2d..	OXAZEPAM

d2e..	*PRAZEPAM
d2f..	BUSPIRONE HYDROCHLORIDE
d2g..	FLUMAZENIL
d71..	AMITRIPTYLINE HYDROCHLORIDE [ANTIDEPRESSANT]
d72..	*BUTRIPTYLINE
d73..	CLOMIPRAMINE HYDROCHLORIDE
d74..	DESIPRAMINE HYDROCHLORIDE
d75..	DOSULEPIN HYDROCHLORIDE
d76..	DOXEPIN
d77..	IMIPRAMINE HYDROCHLORIDE [ANTIDEPRESSANT]
d78..	IPRINDOLE
d79..	LOFEPRAMINE
d7a..	MAPROTILINE HYDROCHLORIDE
d7b..	MIANSERIN HYDROCHLORIDE
d7c..	NORTRIPTYLINE
d7d..	PROTRIPTYLINE HYDROCHLORIDE
d7e..	TRAZODONE HYDROCHLORIDE
d7f..	TRIMIPRAMINE
d7g..	VILOXAZINE HYDROCHLORIDE
d7h..	AMOXAPINE
d81..	PHENELZINE
d83..	ISOCARBOXAZID
d84..	TRANYLCYPROMINE
d85..	MOCLOBEMIDE
d91..	COMPOUND ANTIDEPRESSANTS A-Z
da1..	FLUPENTIXOL [ANTIDEPRESSANT]
da2..	TRYPTOPHAN
da3..	FLUVOXAMINE MALEATE
da4..	FLUOXETINE HYDROCHLORIDE
da5..	SERTRALINE HYDROCHLORIDE
da6..	PAROXETINE HYDROCHLORIDE
da7..	VENLAFAXINE
da9..	CITALOPRAM
daA..	REBOXETINE
daB..	MIRTAZAPINE
daC..	ESCITALOPRAM
daD..	AGOMELATINE
gde..	DULOXETINE

Table 8: Serious Mental Illness related ICD10 codes

ICD10 codes	Descriptions
F200	Paranoid schizophrenia
F201	Hebephrenic schizophrenia

F202	Catatonic schizophrenia
F203	Undifferentiated schizophrenia
F204	Post-schizophrenic depression
F205	Residual schizophrenia
F206	Simple schizophrenia
F208	Other schizophrenia
F209	Schizophreniaunspecified
F21X	Schizotypal disorder
F220	Delusional disorder
F228	Other persistent delusional disorders
F229	Persistent delusional disorderunspecified
F230	Acute polymorphic psychot disord without symp of schizoph'a
F231	Acute polymorphic psychot disord with symp of schizophrenia
F232	Acute schizophrenia-like psychotic disorder
F233	Other acute predominantly delusional psychotic disorders
F238	Other acute and transient psychotic disorders
F239	Acute and transient psychotic disorderunspecified
F24X	Induced delusional disorder
F250	Schizoaffective disordermanic type
F251	Schizoaffective disorderdepressive type
F252	Schizoaffective disordermixed type
F258	Other schizoaffective disorders
F259	Schizoaffective disorderunspecified
F28X	Other nonorganic psychotic disorders
F29X	Unspecified nonorganic psychosis
F300	Hypomania
F301	Mania without psychotic symptoms
F302	Mania with psychotic symptoms
F308	Other manic episodes
F309	Manic episodeunspecified
F310	Bipolar affective disordercurrent episode hypomanic
F311	Bipolar affect disord cur epi manic without psychotic symp
F312	Bipolar affect disorder cur epi manic with psychotic symp
F313	Bipolar affect disorder cur epi mild or moderate depression
F314	Bipolar affect disord cur epi sev depres without psyc symp
F315	Bipolar affect disord cur epi severe depres with psyc symp
F316	Bipolar affective disordercurrent episode mixed
F317	Bipolar affective disordercurrently in remission
F318	Other bipolar affective disorders
F319	Bipolar affective disorderunspecified
F323	Severe depressive episode with psychotic symptoms
F333	Recurrent depress disorder cur epi severe with psyc symp
F39X	Unspecified mood [affective] disorder

Table 9: Serious mental Illness related READ codes

RAED codes	Descriptions
E10..	Schizophrenic disorders
E100.	Simple schizophrenia
E1000	Unspecified schizophrenia
E1001	Subchronic schizophrenia
E1002	Chronic schizophrenic
E1003	Acute exacerbation of subchronic schizophrenia
E1004	Acute exacerbation of chronic schizophrenia
E1005	Schizophrenia in remission
E100z	Simple schizophrenia NOS
E101.	Hebephrenic schizophrenia
E1010	Unspecified hebephrenic schizophrenia
E1011	Subchronic hebephrenic schizophrenia
E1012	Chronic hebephrenic schizophrenia
E1013	Acute exacerbation of subchronic hebephrenic schizophrenia
E1014	Acute exacerbation of chronic hebephrenic schizophrenia
E1015	Hebephrenic schizophrenia in remission
E101z	Hebephrenic schizophrenia NOS
E102.	Catatonic schizophrenia
E1020	Unspecified catatonic schizophrenia
E1021	Subchronic catatonic schizophrenia
E1022	Chronic catatonic schizophrenia
E1023	Acute exacerbation of subchronic catatonic schizophrenia
E1024	Acute exacerbation of chronic catatonic schizophrenia
E1025	Catatonic schizophrenia in remission
E102z	Catatonic schizophrenia NOS
E103.	Paranoid schizophrenia
E1030	Unspecified paranoid schizophrenia
E1031	Subchronic paranoid schizophrenia
E1032	Chronic paranoid schizophrenia
E1033	Acute exacerbation of subchronic paranoid schizophrenia
E1034	Acute exacerbation of chronic paranoid schizophrenia
E1035	Paranoid schizophrenia in remission
E103z	Paranoid schizophrenia NOS
E104.	Acute schizophrenic episode
E105.	Latent schizophrenia
E1050	Unspecified latent schizophrenia
E1051	Subchronic latent schizophrenia
E1052	Chronic latent schizophrenia
E1053	Acute exacerbation of subchronic latent schizophrenia
E1054	Acute exacerbation of chronic latent schizophrenia
E1055	Latent schizophrenia in remission
E105z	Latent schizophrenia NOS

E106.	Residual schizophrenia
E107.	Schizo-affective schizophrenia
E1070	Unspecified schizo-affective schizophrenia
E1071	Subchronic schizo-affective schizophrenia
E1072	Chronic schizo-affective schizophrenia
E1073	Acute exacerbation subchronic schizo-affective schizophrenia
E1074	Acute exacerbation of chronic schizo-affective schizophrenia
E1075	Schizo-affective schizophrenia in remission
E107z	Schizo-affective schizophrenia NOS
E10y.	Other schizophrenia
E10y0	Atypical schizophrenia
E10y1	Coenesthopathic schizophrenia
E10yz	Other schizophrenia NOS
E10z.	Schizophrenia NOS
E110.	Manic disorder, single episode
E1100	Single manic episode, unspecified
E1101	Single manic episode, mild
E1102	Single manic episode, moderate
E1103	Single manic episode, severe without mention of psychosis
E1104	Single manic episode, severe, with psychosis
E1105	Single manic episode in partial or unspecified remission
E1106	Single manic episode in full remission
E110z	Manic disorder, single episode NOS
E111.	Recurrent manic episodes
E1110	Recurrent manic episodes, unspecified
E1111	Recurrent manic episodes, mild
E1112	Recurrent manic episodes, moderate
E1113	Recurrent manic episodes, severe without mention psychosis
E1114	Recurrent manic episodes, severe, with psychosis
E1115	Recurrent manic episodes, partial or unspecified remission
E1116	Recurrent manic episodes, in full remission
E111z	Recurrent manic episode NOS
E1124	Single major depressive episode, severe, with psychosis
E1134	Recurrent major depressive episodes, severe, with psychosis
E114.	Bipolar affective disorder, currently manic
E1140	Bipolar affective disorder, currently manic, unspecified
E1141	Bipolar affective disorder, currently manic, mild
E1142	Bipolar affective disorder, currently manic, moderate
E1143	Bipolar affect disord, currently manic, severe, no psychosis
E1144	Bipolar affect disord, currently manic,severe with psychosis
E1145	Bipolar affect disord,currently manic, part/unspec remission
E1146	Bipolar affective disorder, currently manic, full remission
E114z	Bipolar affective disorder, currently manic, NOS
E115.	Bipolar affective disorder, currently depressed

E1150	Bipolar affective disorder, currently depressed, unspecified
E1151	Bipolar affective disorder, currently depressed, mild
E1152	Bipolar affective disorder, currently depressed, moderate
E1153	Bipolar affect disord, now depressed, severe, no psychosis
E1154	Bipolar affect disord, now depressed, severe with psychosis
E1155	Bipolar affect disord, now depressed, part/unspec remission
E1156	Bipolar affective disorder, now depressed, in full remission
E115z	Bipolar affective disorder, currently depressed, NOS
E116.	Mixed bipolar affective disorder
E1160	Mixed bipolar affective disorder, unspecified
E1161	Mixed bipolar affective disorder, mild
E1162	Mixed bipolar affective disorder, moderate
E1163	Mixed bipolar affective disorder, severe, without psychosis
E1164	Mixed bipolar affective disorder, severe, with psychosis
E1165	Mixed bipolar affective disorder, partial/unspec remission
E1166	Mixed bipolar affective disorder, in full remission
E116z	Mixed bipolar affective disorder, NOS
E117.	Unspecified bipolar affective disorder
E1170	Unspecified bipolar affective disorder, unspecified
E1171	Unspecified bipolar affective disorder, mild
E1172	Unspecified bipolar affective disorder, moderate
E1173	Unspecified bipolar affective disorder, severe, no psychosis
E1174	Unspecified bipolar affective disorder,severe with psychosis
E1175	Unspecified bipolar affect disord, partial/unspec remission
E1176	Unspecified bipolar affective disorder, in full remission
E117z	Unspecified bipolar affective disorder, NOS
E11y.	Other and unspecified manic-depressive psychoses
E11y0	Unspecified manic-depressive psychoses
E11y1	Atypical manic disorder
E11y3	Other mixed manic-depressive psychoses
E11yz	Other and unspecified manic-depressive psychoses NOS
E11z.	Other and unspecified affective psychoses
E11z0	Unspecified affective psychoses NOS
E11zz	Other affective psychosis NOS
E12..	Paranoid states
E120.	Simple paranoid state
E121.	Chronic paranoid psychosis
E122.	Paraphrenia
E123.	Shared paranoid disorder
E12y.	Other paranoid states
E12y0	Paranoia querulans
E12yz	Other paranoid states NOS
E12z.	Paranoid psychosis NOS
E13..	Other nonorganic psychoses

E130.	Reactive depressive psychosis
E131.	Acute hysterical psychosis
E132.	Reactive confusion
E133.	Acute paranoid reaction
E134.	Psychogenic paranoid psychosis
E13y.	Other reactive psychoses
E13y0	Psychogenic stupor
E13y1	Brief reactive psychosis
E13yz	Other reactive psychoses NOS
E13z.	Nonorganic psychosis NOS
E2122	Schizotypal personality
Eu2..	[X]Schizophrenia, schizotypal and delusional disorders
Eu20.	[X]Schizophrenia
Eu200	[X]Paranoid schizophrenia
Eu201	[X]Hebephrenic schizophrenia
Eu202	[X]Catatonic schizophrenia
Eu203	[X]Undifferentiated schizophrenia
Eu204	[X]Post-schizophrenic depression
Eu205	[X]Residual schizophrenia
Eu206	[X]Simple schizophrenia
Eu20y	[X]Other schizophrenia
Eu20z	[X]Schizophrenia, unspecified
Eu21.	[X]Schizotypal disorder
Eu22.	[X]Persistent delusional disorders
Eu220	[X]Delusional disorder
Eu221	[X]Delusional misidentification syndrome
Eu222	[X]Cotard syndrome
Eu223	[X]Paranoid state in remission
Eu22y	[X]Other persistent delusional disorders
Eu22z	[X]Persistent delusional disorder, unspecified
Eu23.	[X]Acute and transient psychotic disorders
Eu230	[X]Acute polymorphic psychot disord without symp of schizoph
Eu231	[X]Acute polymorphic psychot disord with symp of schizophren
Eu232	[X]Acute schizophrenia-like psychotic disorder
Eu233	[X]Other acute predominantly delusional psychotic disorders
Eu23y	[X]Other acute and transient psychotic disorders
Eu23z	[X]Acute and transient psychotic disorder, unspecified
Eu24.	[X]Induced delusional disorder
Eu25.	[X]Schizoaffective disorders
Eu250	[X]Schizoaffective disorder, manic type
Eu251	[X]Schizoaffective disorder, depressive type
Eu252	[X]Schizoaffective disorder, mixed type
Eu25y	[X]Other schizoaffective disorders
Eu25z	[X]Schizoaffective disorder, unspecified

Eu26.	[X]Nonorganic psychosis in remission
Eu2y.	[X]Other nonorganic psychotic disorders
Eu2z.	[X]Unspecified nonorganic psychosis
Eu30.	[X]Manic episode
Eu300	[X]Hypomania
Eu301	[X]Mania without psychotic symptoms
Eu302	[X]Mania with psychotic symptoms
Eu30y	[X]Other manic episodes
Eu30z	[X]Manic episode, unspecified
Eu31.	[X]Bipolar affective disorder
Eu310	[X]Bipolar affective disorder, current episode hypomanic
Eu311	[X]Bipolar affect disorder cur epi manic wout psychotic symp
Eu312	[X]Bipolar affect disorder cur epi manic with psychotic symp
Eu313	[X]Bipolar affect disorder cur epi mild or moderate depressn
Eu314	[X]Bipol aff disord, curr epis sev depress, no psychot symp
Eu315	[X]Bipolar affect dis cur epi severe depres with psyc symp
Eu316	[X]Bipolar affective disorder, current episode mixed
Eu317	[X]Bipolar affective disorder, currently in remission
Eu318	[X]Bipolar affective disorder type I
Eu319	[X]Bipolar affective disorder type II
Eu31y	[X]Other bipolar affective disorders
Eu31z	[X]Bipolar affective disorder, unspecified
Eu323	[X]Severe depressive episode with psychotic symptoms
Eu333	[X]Recurrent depress disorder cur epi severe with psyc symp
