# Supplementary material for: How does the local area deprivation influence life chances for children in poverty in Wales: A record linkage cohort study
Source: SSM Popul Health. 2023 Feb 23;22:101370. doi: 10.1016/j.ssmph.2023.101370 (PMC9986621; doi:10.1016/j.ssmph.2023.101370)
Supplement: Multimedia component 4 [file mmc4.pdf]

Table 1: Substance misuse related ICD10 codes

ICD10 codes	Descriptions
F11	Mental and behavioural disorders due to use of opioids
F110	Mental and behavioural disorders due to use of opioids
F111	Mental and behavioural disorders due to use of opioids
F112	Mental and behavioural disorders due to use of opioids
F113	Mental and behavioural disorders due to use of opioids
F114	Mental and behavioural disorders due to use of opioids
F115	Mental and behavioural disorders due to use of opioids
F116	Mental and behavioural disorders due to use of opioids
F117	Mental and behavioural disorders due to use of opioids
F118	Mental and behavioural disorders due to use of opioids
F119	Mental and behavioural disorders due to use of opioids
F12	Mental and behavioural disorders due to use of cannabinoids
F120	Mental and behavioural disorders due to use of cannabinoids
F121	Mental and behavioural disorders due to use of cannabinoids
F122	Mental and behavioural disorders due to use of cannabinoids
F123	Mental and behavioural disorders due to use of cannabinoids
F124	Mental and behavioural disorders due to use of cannabinoids
F125	Mental and behavioural disorders due to use of cannabinoids
F126	Mental and behavioural disorders due to use of cannabinoids
F127	Mental and behavioural disorders due to use of cannabinoids
F128	Mental and behavioural disorders due to use of cannabinoids
F129	Mental and behavioural disorders due to use of cannabinoids
F13	Mental and behavioural disorders due to use of sedatives or hypnotics
F130	Mental and behavioural disorders due to use of sedatives or hypnotics
F131	Mental and behavioural disorders due to use of sedatives or hypnotics
F132	Mental and behavioural disorders due to use of sedatives or hypnotics
F133	Mental and behavioural disorders due to use of sedatives or hypnotics
F134	Mental and behavioural disorders due to use of sedatives or hypnotics
F135	Mental and behavioural disorders due to use of sedatives or hypnotics
F136	Mental and behavioural disorders due to use of sedatives or hypnotics
F137	Mental and behavioural disorders due to use of sedatives or hypnotics
F138	Mental and behavioural disorders due to use of sedatives or hypnotics
F139	Mental and behavioural disorders due to use of sedatives or hypnotics
F14	Mental and behavioural disorders due to use of cocaine
F140	Mental and behavioural disorders due to use of cocaine
F141	Mental and behavioural disorders due to use of cocaine
F142	Mental and behavioural disorders due to use of cocaine
F143	Mental and behavioural disorders due to use of cocaine
F144	Mental and behavioural disorders due to use of cocaine
F145	Mental and behavioural disorders due to use of cocaine
F146	Mental and behavioural disorders due to use of cocaine

F147	Mental and behavioural disorders due to use of cocaine
F148	Mental and behavioural disorders due to use of cocaine
F149	Mental and behavioural disorders due to use of cocaine
F15	Mental and behavioural disorders due to use of other stimulants, including caffeine
F150	Mental and behavioural disorders due to use of other stimulants, including caffeine
F151	Mental and behavioural disorders due to use of other stimulants, including caffeine
F152	Mental and behavioural disorders due to use of other stimulants, including caffeine
F153	Mental and behavioural disorders due to use of other stimulants, including caffeine
F154	Mental and behavioural disorders due to use of other stimulants, including caffeine
F155	Mental and behavioural disorders due to use of other stimulants, including caffeine
F156	Mental and behavioural disorders due to use of other stimulants, including caffeine
F157	Mental and behavioural disorders due to use of other stimulants, including caffeine
F158	Mental and behavioural disorders due to use of other stimulants, including caffeine
F159	Mental and behavioural disorders due to use of other stimulants, including caffeine
F16	Mental and behavioural disorders due to use of hallucinogens
F160	Mental and behavioural disorders due to use of hallucinogens
F161	Mental and behavioural disorders due to use of hallucinogens
F162	Mental and behavioural disorders due to use of hallucinogens
F163	Mental and behavioural disorders due to use of hallucinogens
F164	Mental and behavioural disorders due to use of hallucinogens
F165	Mental and behavioural disorders due to use of hallucinogens
F166	Mental and behavioural disorders due to use of hallucinogens
F167	Mental and behavioural disorders due to use of hallucinogens
F168	Mental and behavioural disorders due to use of hallucinogens
F169	Mental and behavioural disorders due to use of hallucinogens
F18	Mental and behavioural disorders due to use of volatile solvents
F180	Mental and behavioural disorders due to use of volatile solvents
F181	Mental and behavioural disorders due to use of volatile solvents
F182	Mental and behavioural disorders due to use of volatile solvents
F183	Mental and behavioural disorders due to use of volatile solvents
F184	Mental and behavioural disorders due to use of volatile solvents
F185	Mental and behavioural disorders due to use of volatile solvents
F186	Mental and behavioural disorders due to use of volatile solvents
F187	Mental and behavioural disorders due to use of volatile solvents
F188	Mental and behavioural disorders due to use of volatile solvents
F189	Mental and behavioural disorders due to use of volatile solvents
F19	Mental and behavioural disorders due to multiple drug use and use of other psychoactive substances
F190	Mental and behavioural disorders due to multiple drug use and use of other psychoactive substances
F191	Mental and behavioural disorders due to multiple drug use and use of other psychoactive substances
F192	Mental and behavioural disorders due to multiple drug use and use of other psychoactive substances
F193	Mental and behavioural disorders due to multiple drug use and use of other psychoactive substances

F194	Mental and behavioural disorders due to multiple drug use and use of other psychoactive substances
F195	Mental and behavioural disorders due to multiple drug use and use of other psychoactive substances
F196	Mental and behavioural disorders due to multiple drug use and use of other psychoactive substances
F197	Mental and behavioural disorders due to multiple drug use and use of other psychoactive substances
F198	Mental and behavioural disorders due to multiple drug use and use of other psychoactive substances
F199	Mental and behavioural disorders due to multiple drug use and use of other psychoactive substances
O355	Maternal care for (suspected) damage to fetus by drugs
R781	Finding of opiate drug in blood
R782	Finding of cocaine in blood
R783	Finding of hallucinogen in blood
R784	Finding of other drugs of addictive potential in blood
R785	Finding of psychotropic drug in blood
T40	Poisoning by narcotics and psychodysleptics [hallucinogens]
T400	Poisoning: Opium
T401	Poisoning: Heroin
T402	Poisoning: Other opioids
T403	Poisoning: Methadone
T404	Poisoning: Other synthetic narcotics
T405	Poisoning: Cocaine
T406	Poisoning: Other and unspecified narcotics
T407	Poisoning: Cannabis (derivatives)
T408	Poisoning: Lysergide [LSD]
T409	Poisoning: Other and unspecified psychodysleptics [hallucinogens]
T436	Poisoning: Psychostimulants with abuse potential
X42	Accidental poisoning by and exposure to narcotics and psychodysleptics [hallucinogens], not elsewhere classified
X420	Accidental poisoning by and exposure to narcotics and psychodysleptics [hallucinogens], not elsewhere classified
X421	Accidental poisoning by and exposure to narcotics and psychodysleptics [hallucinogens], not elsewhere classified
X422	Accidental poisoning by and exposure to narcotics and psychodysleptics [hallucinogens], not elsewhere classified
X423	Accidental poisoning by and exposure to narcotics and psychodysleptics [hallucinogens], not elsewhere classified
X424	Accidental poisoning by and exposure to narcotics and psychodysleptics [hallucinogens], not elsewhere classified
X425	Accidental poisoning by and exposure to narcotics and psychodysleptics [hallucinogens], not elsewhere classified
X426	Accidental poisoning by and exposure to narcotics and psychodysleptics [hallucinogens], not elsewhere classified
X427	Accidental poisoning by and exposure to narcotics and psychodysleptics [hallucinogens], not elsewhere classified

X428	Accidental poisoning by and exposure to narcotics and psychodysleptics [hallucinogens], not elsewhere classified
X429	Accidental poisoning by and exposure to narcotics and psychodysleptics [hallucinogens], not elsewhere classified
X62	Intentional self-poisoning by and exposure to narcotics and psychodysleptics [hallucinogens], not elsewhere classified
X620	Intentional self-poisoning by and exposure to narcotics and psychodysleptics [hallucinogens], not elsewhere classified
X621	Intentional self-poisoning by and exposure to narcotics and psychodysleptics [hallucinogens], not elsewhere classified
X622	Intentional self-poisoning by and exposure to narcotics and psychodysleptics [hallucinogens], not elsewhere classified
X623	Intentional self-poisoning by and exposure to narcotics and psychodysleptics [hallucinogens], not elsewhere classified
X624	Intentional self-poisoning by and exposure to narcotics and psychodysleptics [hallucinogens], not elsewhere classified
X625	Intentional self-poisoning by and exposure to narcotics and psychodysleptics [hallucinogens], not elsewhere classified
X626	Intentional self-poisoning by and exposure to narcotics and psychodysleptics [hallucinogens], not elsewhere classified
X627	Intentional self-poisoning by and exposure to narcotics and psychodysleptics [hallucinogens], not elsewhere classified
X628	Intentional self-poisoning by and exposure to narcotics and psychodysleptics [hallucinogens], not elsewhere classified
X629	Intentional self-poisoning by and exposure to narcotics and psychodysleptics [hallucinogens], not elsewhere classified
Y12	Poisoning by and exposure to narcotics and psychodysleptics [hallucinogens], not elsewhere classified, undetermined intent
Y120	Poisoning by and exposure to narcotics and psychodysleptics [hallucinogens], not elsewhere classified, undetermined intent
Y121	Poisoning by and exposure to narcotics and psychodysleptics [hallucinogens], not elsewhere classified, undetermined intent
Y122	Poisoning by and exposure to narcotics and psychodysleptics [hallucinogens], not elsewhere classified, undetermined intent
Y123	Poisoning by and exposure to narcotics and psychodysleptics [hallucinogens], not elsewhere classified, undetermined intent
Y124	Poisoning by and exposure to narcotics and psychodysleptics [hallucinogens], not elsewhere classified, undetermined intent
Y125	Poisoning by and exposure to narcotics and psychodysleptics [hallucinogens], not elsewhere classified, undetermined intent
Y126	Poisoning by and exposure to narcotics and psychodysleptics [hallucinogens], not elsewhere classified, undetermined intent
Y127	Poisoning by and exposure to narcotics and psychodysleptics [hallucinogens], not elsewhere classified, undetermined intent
Y128	Poisoning by and exposure to narcotics and psychodysleptics [hallucinogens], not elsewhere classified, undetermined intent
Y129	Poisoning by and exposure to narcotics and psychodysleptics [hallucinogens], not elsewhere classified, undetermined intent
Z503	Drug rehabilitation
Z715	Drug abuse counselling and surveillance

Z722	Drug use

Table 2: Substance misuse related READ codes

READ codes	Descriptions
13c..	Drug user
13c0.	Injecting drug user
13c1.	Intravenous drug user
13c2.	Never injecting drug user
13c3.	Intramuscular drug user
13c4.	Intranasal drug user
13c5.	Substance misuse increased
13c6.	Substance misuse decreased
13c7.	Current drug user
13c8.	Reduced drugs misuse
13c9.	Subcutaneous drug user
13cA.	Smokes drugs
13cB.	Misuses drugs orally
13cC.	Continuous use of drugs
13cD.	Episodic use of drugs
13cE.	Prolong high dose use cannabis
13cF.	Preoccup with substance misuse
13cG.	Drug tolerance
13cG0	Opioid tolerant
13cG1	Opioid naive
13cH.	Persistent substance misuse
13cJ.	Previously injecting drug user
13cK.	Current non recreat drug user
13cL.	Has never injected drugs
13cM.	Substance misuse
13cM0	Novl psychactve sbstnce misuse
13cM1	Opioid analgesic dependence
13cN.	Has nvr shrd drg injctn equipt
13cQ.	Behavioural tolerance to drug
13cR.	Physical tolerance to drug
13cS.	Psychological drug tolerance
13cT.	Reverse tolerance to drug
1463	H/O: drug dependency
146C.	Failed heroin detoxification
146E.	H/O: recreational drug use
146F.	H/O: drug abuse
1P30.	Compul uncontrollable drug tak
1P31.	Compulsive drug taking
1T...	History of substance misuse

1T0..	H/O heroin misuse
1T00.	H/O daily heroin misuse
1T01.	H/O weekly heroin misuse
1T02.	Prev history of heroin misuse
1T03.	H/O infrequent heroin misuse
1T1..	H/O methadone misuse
1T10.	H/O daily methadone misuse
1T11.	H/O weekly methadone misuse
1T12.	H/O infrequent methadone misus
1T13.	Prev history methadone misuse
1T2..	H/O ecstasy misuse
1T20.	H/O daily ecstasy misuse
1T21.	H/O weekly ecstasy misuse
1T22.	H/O infrequent ecstasy misuse
1T23.	Prev history of ecstasy misuse
1T3..	H/O benzodiazepine misuse
1T30.	H/O daily benzodiazepin misuse
1T31.	H/O weekly benzodiazep misuse
1T32.	H/O infreq benzodiazep misuse
1T33.	Prev H/O benzodiazepine misuse
1T4..	H/O amphetamine misuse
1T40.	H/O daily amphetamine misuse
1T41.	H/O weekly amphetamine misuse
1T42.	H/O infrequent amphetam misuse
1T43.	Prev H/O amphetamine misuse
1T5..	H/O cocaine misuse
1T50.	H/O daily cocaine misuse
1T51.	H/O weekly cocaine misuse
1T52.	H/O infrequent cocaine misuse
1T53.	Prev H/O cocaine misuse
1T6..	H/O crack cocaine misuse
1T60.	H/O daily crack cocaine misuse
1T61.	H/O weekly crack cocain misuse
1T62.	H/O infrequ crack cocain misus
1T63.	Prev H/O crack cocaine misuse
1T7..	H/O hallucinogen misuse
1T70.	H/O daily hallucinogen misuse
1T71.	H/O weekly hallucinogen misuse
1T72.	H/O infrequ hallucinog misuse
1T73.	Prev H/O hallucinogen misuse
1T8..	H/O cannabis misuse
1T80.	H/O daily cannabis misuse
1T81.	H/O weekly cannabis misuse
1T82.	H/O infrequent cannabis misuse

1T83.	Prev H/O cannabis misuse
1T9..	H/O solvent misuse
1T90.	H/O daily solvent misuse
1T91.	H/O weekly solvent misuse
1T92.	H/O infrequent solvent misuse
1T93.	Prev history of solvent misuse
1TA..	H/O barbiturate misuse
1TA0.	H/O daily barbiturate misuse
1TA1.	H/O weekly barbiturate misuse
1TA2.	H/O infrequ barbiturate misuse
1TA3.	Prev H/O barbiturate misuse
1TB..	H/O major tranquilliser misuse
1TB0.	H/O daily maj tranquilli misus
1TB1.	H/O weekl maj tranquilli misus
1TB2.	H/O infreq maj trnquillis miss
1TB3.	Prev H/O major tranq misuse
1TC..	H/O anti-depressant misuse
1TC0.	H/O daily anti-depress misuse
1TC1.	H/O weekly anti-depress misuse
1TC2.	H/O infreq anti-depress misuse
1TC3.	Prev H/O anti-depressnt misuse
1TD..	H/O opiate misuse
1TD0.	H/O daily opiate misuse
1TD1.	H/O weekly opiate misuse
1TD2.	H/O infrequent opiate misuse
1TD3.	Prev history of opiate misuse
1TE..	Uses heroin on top subst ther
1TF..	Dsnt use heroin top subst ther
1TG..	H/O nov psychoact subst misuse
1V...	Drug misuse behaviour
1V0..	Misuses drugs
1V00.	Occasional drug user
1V01.	Long-term drug misuser
1V02.	Poly-drug misuser
1V03.	Misuses drugs sublingually
1V04.	Misuses drugs rectally
1V05.	Misuses drugs vaginally
1V06.	Uses drug paraphernalia
1V07.	Notified addict
1V08.	Smokes drugs in cigarette form
1V09.	Smokes drugs through a pipe
1V0A.	Chases the dragon
1V0B.	Sniffs drugs
1V0C.	Drug addict

1V0D.	Am spent per day on drug habit
1V0E.	Health prob sec to drug misuse
1V1..	Time devotd drug rel activties
1V10.	Time spent obtaining drugs
1V11.	Time spent taking drugs
1V12.	Time spent recover from drugs
1V2..	Frequency of drug misuse
1V22.	Age at starting drug misuse
1V23.	Time since stopped drug misuse
1V24.	Total time drugs misused
1V26.	Misused drugs in past
1V3..	Drug injection behaviour
1V30.	Injects drugs subcutaneously
1V31.	Injects drugs intramuscularly
1V32.	Neck injector
1V33.	Groin injector
1V34.	Does not inject drugs
1V35.	Shares drug equipment
1V36.	Frontloading
1V37.	Drug inject equipment hygiene
1V38.	Sharing drug inject equipment
1V3A.	Not share drug inject equipmen
1V3B.	Shares syringes
1V3C.	Shares needles
1V3D.	Cleaning of needles
1V3E.	Cleans own needles
1V3F.	Cleans needles with bleach
1V3G.	Does not clean needles
1V3H.	Obtains clean needles
1V3J.	Uses needle exchange scheme
1V3K.	Obtains clean syringes
1V3L.	Needle syringe exch scheme use
1V3M.	Needle + syringe exch not used
1V3N.	Needle and syringe exch used
1V4..	Priority of drug activity
1V40.	No priority to drug activities
1V41.	Priority to drug activities
1V42.	Drug priority ov social obligs
1V43.	Drug priority over family
1V44.	Drug priority ov finance oblig
1V5..	Routine drug-related activity
1V50.	No routine of drug activities
1V51.	Has routine of drug activities
1V52.	Same drug routine every day

1V53.	Drug-related rituals
1V54.	Follows drug-related rituals
1V55.	Not follow drug-relate rituals
1V6..	Drug-relat offending behaviour
1V64.	Illicit drug use
1V65.	Heroin misuse
1V66.	Ecstasy misuse
677T.	Subst misuse structurd counsel
7P220	Delivery rehab drug addiction
8AA..	Drug abuse monitoring
8B23.	Drug addiction therapy
8B230	Drug add maint ther naltrexone
8B231	Drug add maint ther lofexidine
8B2M.	Buprenorphine maintenance ther
8B2N.	Drug add detox ther methadone
8B2P.	Drug add maint ther methadone
8B2Q.	Drug add maint ther buprenorph
8B2R.	Drug add detox ther buprenorph
8B2S.	Opioid agonist substitut thera
8B2T.	Opioid antagonist therapy
8BA9.	Detoxification dependence drug
8BAW.	Drug depen self detoxification
8BAX.	Drug depen home detoxification
8BAc.	Subs mis mgt stop - self withd
8BAd.	Opiate dependence detoxificatn
8BAt.	Drug relapse prevention
8BAv.	Drug harm reduction programme
8BAx.	Drug twelve step programme
8BE..	Maintenance therapy
8BE0.	Reinduct methadone maint thera
8BE1.	Reinduct buprenorph maint ther
8CR9.	Benzodiazepi clinical mgt plan
8FB..	Drug rehabilitation
8FB0.	Drug detox programme completed
8H7x.	Refer to drug abuse counsellor
8HHL.	Ref to comm drug dependen team
8HHd.	Referral to drug treatment cen
8HHe.	Referral to com drug alco team
8Hh1.	Self refer substanc misus serv
8HkF.	Refer substance misuse service
8Hl5.	Referral to drugs therapist
8Hl6.	Referral to drugs worker
8Hq..	Admsn substnc misuse detox cnt
8I2N.	Drug depend home detox contra

8IE7.	Substance misuse assess declin
9G2..	Drug addiction notification
9G21.	Drug addict notific to CMO
9G22.	Drug addict re-notific due
9G23.	Drug addict re-notif to CMO
9G2Z.	Drug addiction notif NOS
9HC..	Substance misuse monitoring
9HC0.	Initial substance misuse asses
9HC1.	Follow up substa misuse assess
9HC2.	Subst mis clin man plan agreed
9HC3.	Subst mis clin man plan review
9HC4.	Sub misuse treatment withdrawn
9HC5.	Sub misus treat prog completed
9HC6.	Substance misuse treatm declin
9HC7.	Subst misuse treat not availbl
9HC8.	Decl to give subst misuse hist
9HC9.	Snc mse tmnt gvn othr hcr prdr
9HCA.	Sbstnce misuse mntr 6 mnth rvw
9HCB.	Substance misuse mntr annl rvw
9HCC.	On substance misuse programme
9N0Z.	Seen in drug rehab centre
9N1yJ	Seen in drug misuse clinic
9N6a.	Refer by drug statutor service
9N6b.	Ref by drug non-statutory serv
9N6g.	Refer by syringe excha service
9NN1.	Under care community drug team
9NX2.	In-house subs misuse treatment
9NdN.	Declnd consnt notif drug misus
9No5.	Seen in substance misuse clinc
9k5..	Drug misuse - enhan serv admin
9k50.	Drug misuse - enh serv complet
9k51.	Share care drug misu trt - ESA
9k52.	Drug misus trt prim care - ESA
9k53.	Phrmcy attend drug misus - ESA
9kS..	Drug mis asse decl - enha serv
E02..	Drug psychoses
E020.	Drug withdrawal syndrome
E021.	Drug-induced paranoia/hallucin
E0210	Drug-induced paranoid state
E0211	Drug-induced hallucinosis
E021z	Drug-induc.paranoia/halluc NOS
E022.	Pathological drug intoxication
E02y.	Other drug psychoses
E02y0	Drug-induced delirium

E02y1	Drug-induced dementia
E02y2	Drug-induced amnestic syndrome
E02y3	Drug-induced depressive state
E02y4	Drug-induced personality dis.
E02yz	Other drug psychoses NOS
E02z.	Drug psychosis NOS
E24..	Drug dependence
E240.	Opioid type drug dependence
E2400	Opioid dependence-unspecified
E2401	Opioid dependence-continuous
E2402	Opioid dependence - episodic
E2403	Opioid dependence-in remission
E240z	Opioid drug dependence NOS
E241.	Hypnotic/anxiolytic dependence
E2410	Hypnotic/anxiol.depend.-unspec
E2411	Hypnot/anxiol.dep.-continuous
E2412	Hypnot/anxiol.dep.-episodic
E2413	Hypnot/anxiol.dep-in remission
E241z	Hypnotic/anxiolytic depend.NOS
E242.	Cocaine type drug dependence
E2420	Cocaine dependence-unspecified
E2421	Cocaine dependence-continuous
E2422	Cocaine dependence-episodic
E2423	Cocaine depend. - in remission
E242z	Cocaine drug dependence NOS
E243.	Cannabis type drug dependence
E2430	Cannabis dependence-unspecif.
E2431	Cannabis dependence-continuous
E2432	Cannabis dependence-episodic
E2433	Cannabis depend.- in remission
E243z	Cannabis drug dependence NOS
E244.	Amphetamine/psychostim.depend.
E2440	Amphetamine depend.-unspecif.
E2441	Amphetamine depend.-continuous
E2442	Amphetamine depend.-episodic
E2443	Amphetamine dep.-in remission
E244z	Amphetamine dependence NOS
E245.	Hallucinogen dependence
E2450	Hallucinogen depend.-unspecif.
E2451	Hallucinogen depend-continuous
E2452	Hallucinogen depend.-episodic
E2453	Hallucinogen dep.-in remission
E245z	Hallucinogen dependence NOS
E246.	Glue sniffing dependence

E2460	Glue sniffing - unspecified
E2461	Glue sniffing - continuous
E2462	Glue sniffing - episodic
E2463	Glue sniffing - in remission
E246z	Glue sniffing dependence NOS
E247.	Other specified drug dependen.
E2470	Other drug dependence unspecif
E2471	Other drug depend.-continuous
E2472	Other drug depend.-episodic
E2473	Other drug dep.-in remission
E247z	Other drug dependence NOS
E248.	Combined opioid+other drug dep
E2480	Opioid+other drug dep. unspec.
E2481	Continuous opioid+other depen.
E2482	Episodic opioid+other depend.
E2483	In remission-opioid+other dep.
E248z	Opioid+other drug depend. NOS
E249.	Combined drug dep. excl.opioid
E2490	Comb.drug dep ex opioid-unspec
E2491	Comb.drug dep ex opioid-contin
E2492	Comb.drug dep ex opioid-episod
E2493	Comb.drug dep ex opioid-in rem
E249z	Comb.drug dep ex opioid NOS
E24A.	Ecstasy type drug dependence
E24z.	Drug dependence NOS
E25..	Nondependent abuse of drugs
E252.	Nondependent cannabis abuse
E2520	Nondep cannabis abuse - unspec
E2521	Nondep cannabis abuse - contin
E2522	Nondep cannabis abuse - episod
E2523	Nondep cannabis abuse in remis
E252z	Nondep cannabis abuse NOS
E253.	Nondependen hallucinogen abuse
E2530	Nondep hallucinogen abuse-unsp
E2531	Nondep hallucinogen abuse-cont
E2532	Nondep hallucinogen abuse-epis
E2533	Nondep hallucin abuse-in remis
E253z	Nondep hallucinogen abuse NOS
E254.	Nondep hypnot/anxiolytic abuse
E2540	Nondep hypnot/anxio.abuse-unsp
E2541	Nondep hypnot/anxio.abuse-cont
E2542	Nondep hypnot/anxio.abuse-epis
E2543	Nondep hypn/anxio.abuse-in rem
E254z	Nondep hypnot/anxiol abuse NOS

E255.	Nondependent opioid abuse
E2550	Nondep opioid abuse - unspecif
E2551	Nondep opioid abuse - continuo
E2552	Nondep opioid abuse - episodic
E2553	Nondep opioid abuse - in remis
E255z	Nondependent opioid abuse NOS
E256.	Nondependent cocaine abuse
E2560	Nondep cocaine abuse - unspec.
E2561	Nondep cocaine abuse - contin.
E2562	Nondep cocaine abuse - episod.
E2563	Nondep cocaine abuse -in remis
E256z	Nondependent cocaine abuse NOS
E257.	Nondep amphetamine type abuse
E2570	Nondep amphet type abuse -unsp
E2571	Nondep amphet type abuse -cont
E2572	Nondep amphet type abuse -epis
E2573	Nondep amph. type abuse-in rem
E257z	Nondep amphet. type abuse NOS
E258.	Nondep antidepress type abuse
E2580	Nondep antidep type abuse-unsp
E2581	Nondep antidep type abuse-cont
E2582	Nondep antidep type abuse-epis
E2583	Nondep antidep tp abuse-in rem
E258z	Nondep antidep type abuse NOS
E259.	Nondependent mixed drug abuse
E2590	Nondep mixed drug abuse-unspec
E2591	Nondep mixed drug abuse-contin
E2592	Nondep mixed drug abuse-episod
E2593	Nondep mixed drug abuse-in rem
E2594	Misuse of prescription drugs
E259z	Nondep mixed drug abuse NOS
E25y.	Nondependent other drug abuse
E25y0	Nondep other drug abuse-unspec
E25y1	Nondep other drug abuse-contin
E25y2	Nondep other drug abuse-episod
E25y3	Nondep other drug abuse-in rem
E25yz	Nondep other drug abuse NOS
E25z.	Misuse of drugs NOS
Eu1..	[X]Mental dis, psychoact subst
Eu11.	[X]Mental dis due to opioids
Eu110	[X]Acute opioid intoxication
Eu111	[X]Harmful use of opioids
Eu112	[X]Opioid dependence syndrome
Eu113	[X]Opioid withthdrawal state

Eu114	[X]Opioid withdrawal delirium
Eu115	[X]Psychot dis due to opioids
Eu116	[X]Amnesic synd due to opioids
Eu117	[X]Resid psychotic due opioid
Eu11y	[X]Oth ment/beh dis due opioid
Eu11z	[X]Uns ment/beh dis due opioid
Eu12.	[X]Mental dis due cannabinoids
Eu120	[X]Acute cannabis intoxication
Eu121	[X]Harmful use of cannabis
Eu122	[X]Cannabis dependence syndrom
Eu123	[X]Cannabis withdrawal state
Eu124	[X]Cannabis withdrawl delirium
Eu125	[X]Psychot dis due to cannabis
Eu126	[X]Amnesic synd due cannabis
Eu127	[X]Resid psychot due cannabis
Eu12y	[X]Oth ment/beh dis cannabinds
Eu12z	[X]Unsp mnt/beh dis cannabinds
Eu13.	[X]Mental dis due sedat/hypnot
Eu130	[X]Acute sedat/hypnotic intox
Eu131	[X]Harmful use sedat/hypnotic
Eu132	[X]Sedat/hypnotic depend syndr
Eu133	[X]Sedat/hypnot withdraw state
Eu134	[X]Sed/hypn withdraw delirium
Eu135	[X]Psychot dis due sedat/hypn
Eu136	[X]Amnesic synd due sedat/hypn
Eu137	[X]Resid psychot due sed/hypn
Eu13y	[X]Oth ment/beh dis sed/hypnot
Eu13z	[X]Uns ment/beh dis sed/hypnot
Eu14.	[X]Mental dis due use cocaine
Eu140	[X]Acute cocaine intoxication
Eu141	[X]Harmful use of cocaine
Eu142	[X]Cocaine dependence syndrome
Eu143	[X]Cocaine withdrawal state
Eu144	[X]Cocaine withdrawal delirium
Eu145	[X]Psychot dis due to cocaine
Eu146	[X]Amnesic synd due to cocaine
Eu147	[X]Resid psychot due cocaine
Eu14y	[X]Ot ment/beh dis due cocaine
Eu14z	[X]Uns ment/bh dis due cocaine
Eu15.	[X]Ment dis oth stimul/caffein
Eu150	[X]Acute intoxic oth stimulant
Eu151	[X]Harmful use other stimulant
Eu152	[X]Oth stimulant dependen synd
Eu153	[X]Oth stimulant withdr state

Eu154	[X]Oth stimulant withdr delir
Eu155	[X]Psychotic dis oth stimulant
Eu156	[X]Amnesic syndr oth stimulant
Eu157	[X]Resid psychot oth stimulant
Eu15y	[X]Oth ment/beh dis stimulant
Eu15z	[X]Uns ment/beh dis stimulant
Eu16.	[X]Mental disord hallucinogens
Eu160	[X]Acute hallucinogen intoxic
Eu161	[X]Harmful use hallucinogens
Eu162	[X]Hallucinogen depend synd
Eu163	[X]Hallucinogen withdraw state
Eu164	[X]Hallucin withdraw delirium
Eu165	[X]Psychotic due hallucinogen
Eu166	[X]Amnesic synd due hallucinog
Eu167	[X]Resid psychot hallucinogen
Eu16y	[X]Oth ment/beh dis hallucinog
Eu16z	[X]Uns ment/beh dis hallucinog
Eu18.	[X]Ment dis volatile solvents
Eu180	[X]Acute solvent intoxication
Eu181	[X]Harmful use of solvents
Eu182	[X]Solvent dependence syndrome
Eu183	[X]Solvent withdrawal state
Eu184	[X]Solvent withdrawal delirium
Eu185	[X]Psychotic dis due solvent
Eu186	[X]Amnesic syndr due solvent
Eu187	[X]Resid psychotic due solvent
Eu18y	[X]Ot ment/beh dis due solvent
Eu18z	[X]Uns ment/beh due solvent
Eu19.	[X]Ment disord multi drug use
Eu190	[X]Acute intox multi drug use
Eu191	[X]Harmful use multiple drugs
Eu192	[X]Multiple drug dependence
Eu193	[X]Multiple drug withdrawal
Eu194	[X]Multi drug withdr delirium
Eu195	[X]Psychotic due multi drugs
Eu196	[X]Amnesic syn due multi drugs
Eu197	[X]Resid psychotic multi drugs
Eu19y	[X]Ot ment/beh due multi drugs
Eu19z	[X]Un ment/beh due multi drugs
Eu1A.	[X]Men behav dis due crack coc
Eu1A0	[X]Acute crack cocaine intoxic
Eu1A1	[X]Harmful use crack cocaine
Eu1A2	[X]Crack cocaine depend synd
Eu1A3	[X]Crack cocaine withdraw stat

Eu1A4	[X]Crack coc withdraw stat del
Eu1A5	[X]Crack cocaine psychotic dis
Eu1A6	[X]Crack cocaine amnesic synd
Eu1A7	[X]Cra coc res late-on psy dis
Eu1Ay	[X]Crac coc other ment beh dis
Eu1Az	[X]Crack coc unsp men beh dis
L183.	Pregnancy+drug dependence
L1830	Preg.+drug dependence unspecif
L1831	Preg.+drug dependence-deliver.
L1832	Preg.+drug depend-del+p/n comp
L1833	Preg.+drug depend-not deliver.
L1834	Preg.+drug depend.+p/n complic
L183z	Preg.+drug dependence NOS
L255.	Fetus+drug damage
L2550	Fetus+drug damage unspecified
L2551	Fetus+drug damage-delivered
L2552	Fetus+drug damage+a/n problem
L255z	Fetus+drug damage NOS
R10B0	[D]Finding of cocain in blood
R10B1	[D]Find hallucinogen in blood
R10B2	[D]Find psychotrop drug blood
R10B4	[D]Finding, opiate drug in bld
Ryu86	[X]Find ot drg addic poten,bld
SL50.	Opiate/narcotic poisoning
SL500	Unspecified opium poisoning
SL501	Heroin poisoning
SL502	Methadone poisoning
SL50z	Opiate/narcotic poisoning NOS
SL850	Cocaine poisoning
SL96.	Hallucinogen poisoning
SL960	Cannabis poisoning
SL961	Lysergide (LSD) poisoning
SL963	Mescaline poisoning
SL964	Psilocybin poisoning
SL96z	Hallucinogen poisoning NOS
SL97.	Psychostimulant poisoning
SL970	Amfetamine poisoning
SL972	Ecstasy poisoning
SL97z	Psychostimulant poisoning NOS
SyuFB	[X]Poisoning by other opioids
SyuFC	[X]Poisoning by oth synth narc
SyuFD	[X]Poisoning by oth/unsp narc
SyuFE	[X]Pois,oth/un psychodysl/hall
T800.	Accid.pois.- heroin

T801.	Accid.pois.- methadone
T8023	Accid.pois.- opium
T841.	Accid.pois.- hallucinogens
T8410	Accid.pois.- cannabis derivat.
T8413	Accid.pois.- mescaline
T8414	Accid.pois.- psilocin
T8415	Accid.pois.- psilocybin
T842.	Accid.pois.- psychostimulants
T8420	Accid.pois.- amphetamine
T8520	Accid.pois.- cocaine
U1A5.	[X]Accident poisoning narcotic
U1A50	[X]Acc poison narcotic home
U1A51	[X]Ac pois narcotic res ins
U1A52	[X]Ac pois narcotic pub ins
U1A53	[X]Ac pois narcotic sport ar
U1A54	[X]Ac pois narcotic on hway
U1A55	[X]Ac pois narcotic trade ar
U1A56	[X]Ac pois narcotic indus ar
U1A57	[X]Ac pois narcotic on farm
U1A5y	[X]Ac pois narcotic OS place
U1A5z	[X]Ac pois narcotic unsp pl
U1A6.	[X]Acc poisoning hallucinogens
U1A60	[X]Acc poison hallucinog home
U1A61	[X]Ac pois hallucinog res ins
U1A62	[X]Ac pois hallucinog pub ins
U1A63	[X]Ac pois hallucinog sport ar
U1A64	[X]Ac pois hallucinog on hway
U1A65	[X]Ac pois hallucinog trade ar
U1A66	[X]Ac pois hallucinog indus ar
U1A67	[X]Ac pois hallucinog on farm
U1A6y	[X]Ac pois hallucinog OS place
U1A6z	[X]Ac pois hallucinog unsp pl
U205.	[X]Intent self poison narcotic
U2050	[X]Self pois narcotic home
U2051	[X]S/pois narcotic res ins
U2052	[X]S/pois narcotic pub ins
U2053	[X]S/pois narcotic sport ar
U2054	[X]S/pois narcotic on hway
U2055	[X]S/pois narcotic trade ar
U2056	[X]S/pois narcotic indus ar
U2057	[X]S/pois narcotic on farm
U205y	[X]S/pois narcotic OS place
U205z	[X]S/pois narcotic unsp pl
U206.	[X]Int s/poising hallucinogens

U2060	[X]Self pois hallucinog home
U2061	[X]S/pois hallucinog res ins
U2062	[X]S/pois hallucinog pub ins
U2063	[X]S/pois hallucinog sport ar
U2064	[X]S/pois hallucinog on hway
U2065	[X]S/pois hallucinog trade ar
U2066	[X]S/pois hallucinog indus ar
U2067	[X]S/pois hallucinog on farm
U206y	[X]S/pois hallucinog OS place
U206z	[X]S/pois hallucinog unsp pl
U405.	[X]Poisoning ?intent narcotic
U4050	[X]Pois ?intent narcotic home
U4051	[X]Pois ?int narcotic resid
U4052	[X]Pois ?int narcotic pub ins
U4053	[X]Pois ?int narcotic sport ar
U4054	[X]Pois ?int narcotic on hway
U4055	[X]Pois ?int narcotic trade ar
U4056	[X]Pois ?int narcotic indus ar
U4057	[X]Pois ?int narcotic on farm
U405y	[X]Pois ?int narcotic OS place
U405z	[X]Pois ?int narcotic unsp pl
U406.	[X]Poison ?intent hallucinogen
U4060	[X]Pois ?intent hallucin home
U4061	[X]Pois ?int hallucinog resid
U4062	[X]Pois ?int hallucin pub ins
U4063	[X]Pois ?int hallucin sport ar
U4064	[X]Pois ?int hallucin on hway
U4065	[X]Pois ?int hallucin trade ar
U4066	[X]Pois ?int hallucin indus ar
U4067	[X]Pois ?int hallucin on farm
U406y	[X]Pois ?int hallucin OS place
U406z	[X]Pois ?int hallucin unsp pl
ZV114	[V]Pers hist subst abuse
ZV4K1	[V]Drug use
ZV6D7	[V]Drug abuse counsel+surveiln
dj36.	SUBUTEX 400micrograms s/l tabs
dj37.	SUBUTEX 2mg sublingual tablets
dj38.	SUBUTEX 8mg sublingual tablets
dj3D.	BUPRNRPHNE+NALOXN 2/0.5mg tabs
dj3E.	SUBOXONE 2mg/0.5mg s/l tabs
dj3F.	BUPRNRPHNE+NALOXN 8mg/2mg tabs
dj3G.	SUBOXONE 8mg/2mg s/l tabs
dj3K.	NATZON 400micrograms s/l tabs
dj3L.	NATZON 2mg sublingual tablets

dj3M.	NATZON 8mg sublingual tablets
dj3N.	GABUP 400micrograms s/l tabs
dj3O.	GABUP 1mg sublingual tablets
dj3P.	GABUP 2mg sublingual tablets
dj3Q.	GABUP 4mg sublingual tablets
dj3R.	GABUP 6mg sublingual tablets
dj3S.	GABUP 8mg sublingual tablets
dj3T.	BUPRENORPHINE 1mg s/l tabs
dj3U.	BUPRENORPHINE 4mg s/l tabs
dj3V.	BUPRENORPHINE 6mg s/l tabs
dj3c.	PREFIBIN 400mcg sublingual tab
dj3d.	PREFIBIN 2mg sublingual tabs
dj3e.	PREFIBIN 8mg sublingual tabs
dj3u.	BUPRENORPHINE 2mg s/l tabs
dj3v.	BUPRENORPHINE 8mg s/l tabs
djc..	METHADONE HCL [ANALGESIC]
djc1.	PHYSEPTONE 5mg tablets
djc2.	PHYSEPTONE 10mg/1mL injection
djc3.	METHADONE 1mg/1mL mixture
djc4.	METHADONE HCL 50mg/5mL s/f liq
djc5.	MARTINDALE METHADONE DTF mixt
djc6.	METHODEX 1mg/1mL mixture
djc7.	METHADOSE 10mg/mL s/f liq
djc8.	METHADONE HCL 20mg/mL s/f liq
djc9.	METHADOSE 20mg/mL s/f liq
djcA.	METHADONE DILUENT liquid
djcB.	METHADOSE DILUENT liquid
djcC.	METHADONE 1mg/1mL s/f mixt
djcD.	METHAROSE 1mg/1mL s/f soln
djcE.	*PINADONE 1mg/1mL mixture
djcF.	*PINADONE 1mg/1mL s/f mixt
djcG.	PHYSEPTONE 20mg/2mL injection
djcH.	PHYSEPTONE 35mg/3.5mL inj
djcJ.	PHYSEPTONE 50mg/5mL injection
djcK.	PHYSEPTONE 1mg/1mL s/f mixture
djcL.	PHYSEPTONE 1mg/1mL mixture
djcM.	SYNASTONE 10mg/1mL injection
djcN.	SYNASTONE 20mg/2mL injection
djcO.	SYNASTONE 35mg/3.5mL injection
djcP.	SYNASTONE 50mg/5mL injection
djcQ.	SYNASTONE 50mg/2mL injection
djcR.	SYNASTONE 50mg/1mL injection
djcS.	PHYSEPTONE 50mg/2mL injection
djcT.	PHYSEPTONE 50mg/1mL injection

djcU.	EPTADONE 1mg/mL oral solution
djcV.	EPTADONE 5mg/mL oral solution
djcW.	EPTADONE 20mg/20mL oral soln
djcX.	EPTADONE 40mg/40mL oral soln
djcY.	EPTADONE 60mg/60mL oral soln
djcZ.	EPTADONE 100mg/20mL oral soln
djco.	METHADONE 20mg/20mL oral soln
djcp.	METHADONE 40mg/40mL oral soln
djcq.	METHADONE 60mg/60mL oral soln
djcr.	METHADONE 100mg/20mL oral soln
djcs.	METHADONE 5mg/mL oral solution
djct.	METHADONE HCL 50mg/2mL inj
djcu.	METHADONE HCL 50mg/1mL inj
djcv.	METHADONE HCL 20mg/2mL inj
djcw.	METHADONE HCL 35mg/3.5mL inj
djcx.	METHADONE HCL 50mg/5mL inj
djcy.	METHADONE HCL 5mg tablets
djcz.	METHADONE HCL 10mg/1mL inj
du2..	NALTREXONE HYDROCHLORIDE
du21.	NALTREXONE HCL 50mg tablets
du22.	NALOREX 50mg tablets
du23.	OPIZONE 50mg tablets
du24.	ADEPEND 50mg tablets
du4..	LOFEXIDINE HYDROCHLORIDE
du41.	BRITLOFEX 200mcg tablets
du42.	LOFEXIDINE HCL 200mcg tablets
