# Supplementary material for: How does the local area deprivation influence life chances for children in poverty in Wales: A record linkage cohort study
Source: SSM Popul Health. 2023 Feb 23;22:101370. doi: 10.1016/j.ssmph.2023.101370 (PMC9986621; doi:10.1016/j.ssmph.2023.101370)
Supplement: Multimedia component 5 [file mmc5.pdf]

Table 1: Alcohol related ICD10 codes

ICD10 Codes	Descriptions
E244	Alcohol-induced pseudo-Cushing's syndrome
E512	Wernicke's encephalopathy
F10	Mental and behavioural disorders due to use of alcohol
F100	Mental and behavioural disorders due to use of alcohol
F101	Mental and behavioural disorders due to use of alcohol
F102	Mental and behavioural disorders due to use of alcohol
F103	Mental and behavioural disorders due to use of alcohol
F104	Mental and behavioural disorders due to use of alcohol
F105	Mental and behavioural disorders due to use of alcohol
F106	Mental and behavioural disorders due to use of alcohol
F107	Mental and behavioural disorders due to use of alcohol
F108	Mental and behavioural disorders due to use of alcohol
F109	Mental and behavioural disorders due to use of alcohol
G312	Degeneration of nervous system due to alcohol
G621	Alcoholic polyneuropathy
G721	Alcoholic myopathy
I426	Alcoholic cardiomyopathy
K292	Alcoholic gastritis
K70	Alcoholic liver disease
K700	Alcoholic fatty liver
K701	Alcoholic hepatitis
K702	Alcoholic fibrosis and sclerosis of liver
K703	Alcoholic cirrhosis of liver
K704	Alcoholic hepatic failure
K709	Alcoholic liver disease, unspecified
K852	Alcohol-induced acute pancreatitis
K860	Alcohol-induced chronic pancreatitis
O354	Maternal care for (suspected) damage to fetus from alcohol
R780	Finding of alcohol in blood
T51	Toxic effect of alcohol
T510	Toxic effect: Ethanol
T511	Toxic effect: Methanol
T512	Toxic effect: 2-Propanol
T513	Toxic effect: Fusel oil
T518	Toxic effect: Other alcohols
T519	Toxic effect: Alcohol, unspecified
X45	Accidental poisoning by and exposure to alcohol
X450	Accidental poisoning by and exposure to alcohol
X451	Accidental poisoning by and exposure to alcohol
X452	Accidental poisoning by and exposure to alcohol
X453	Accidental poisoning by and exposure to alcohol
X454	Accidental poisoning by and exposure to alcohol

X455	Accidental poisoning by and exposure to alcohol
X456	Accidental poisoning by and exposure to alcohol
X457	Accidental poisoning by and exposure to alcohol
X458	Accidental poisoning by and exposure to alcohol
X459	Accidental poisoning by and exposure to alcohol
X65	Intentional self-poisoning by and exposure to alcohol
X650	Intentional self-poisoning by and exposure to alcohol
X651	Intentional self-poisoning by and exposure to alcohol
X652	Intentional self-poisoning by and exposure to alcohol
X653	Intentional self-poisoning by and exposure to alcohol
X654	Intentional self-poisoning by and exposure to alcohol
X655	Intentional self-poisoning by and exposure to alcohol
X656	Intentional self-poisoning by and exposure to alcohol
X657	Intentional self-poisoning by and exposure to alcohol
X658	Intentional self-poisoning by and exposure to alcohol
X659	Intentional self-poisoning by and exposure to alcohol
Y15	Poisoning by and exposure to alcohol, undetermined intent
Y150	Poisoning by and exposure to alcohol, undetermined intent
Y151	Poisoning by and exposure to alcohol, undetermined intent
Y152	Poisoning by and exposure to alcohol, undetermined intent
Y153	Poisoning by and exposure to alcohol, undetermined intent
Y154	Poisoning by and exposure to alcohol, undetermined intent
Y155	Poisoning by and exposure to alcohol, undetermined intent
Y156	Poisoning by and exposure to alcohol, undetermined intent
Y157	Poisoning by and exposure to alcohol, undetermined intent
Y158	Poisoning by and exposure to alcohol, undetermined intent
Y159	Poisoning by and exposure to alcohol, undetermined intent
Y573	Alcohol deterrents
Y900	Blood alcohol level of less than 20 mg/100 ml
Y901	Blood alcohol level of 20-39 mg/100 ml
Y902	Blood alcohol level of 40-59 mg/100 ml
Y903	Blood alcohol level of 60-79 mg/100 ml
Y904	Blood alcohol level of 80-99 mg/100 ml
Y905	Blood alcohol level of 100-119 mg/100 ml
Y906	Blood alcohol level of 120-199 mg/100 ml
Y907	Blood alcohol level of 200-239 mg/100 ml
Y908	Blood alcohol level of 240 mg/100 ml or more
Y909	Presence of alcohol in blood, level not specified
Y910	Mild alcohol intoxication
Y911	Moderate alcohol intoxication
Y912	Severe alcohol intoxication
Y913	Very severe alcohol intoxication
Y919	Alcohol involvement, not otherwise specified
Z502	Alcohol rehabilitation

Z714	Alcohol abuse counselling and surveillance
Z721	Alcohol use

Table 2: Alcohol related READ codes

READ codes	Descriptions
136..	Alcohol consumption
1362.	Trivial drinker - <1u/day
1363.	Light drinker - 1-2u/day
1364.	Moderate drinker - 3-6u/day
1365.	Heavy drinker - 7-9u/day
1366.	Very heavy drinker - >9u/day
1368.	Alcohol consumption unknown
1369.	Suspect alcohol abuse - denied
136F.	Spirit drinker
136G.	Beer drinker
136H.	Drinks beer and spirits
136I.	Drinks wine
136J.	Social drinker
136K.	Alcohol intake above recommended sensible limits
136L.	Alcohol intake within recommended sensible limits
136N.	Light drinker
136O.	Moderate drinker
136P.	Heavy drinker
136Q.	Very heavy drinker
136R.	Binge drinker
136S.	Hazardous alcohol use
136T.	Harmful alcohol use
136V.	Alcohol units per week
136W.	Alcohol misuse
136X.	Alcohol units consumed on heaviest drinking day
136Y.	Drinks in morning to get rid of hangover
136Z.	Alcohol consumption NOS
136a.	Increasing risk drinking
136b.	Feels should cut down drinking
136c.	Higher risk drinking
136d.	Lower risk drinking
136e.	Declines to state current alcohol consumption
13Y8.	Alcoholics anonymous
13ZY.	Disqualified from driving due to excess alcohol
1462.	H/O: alcoholism
1B1c.	Alcohol induced hallucinations
1F9D.	Replaces meals with drinks
2126C	Alcohol dependence resolved

2577.	O/E - breath - alcohol smell
388u.	Fast alcohol screening test
38D2.	Single alcohol screening questionnaire
38D3.	Alcohol use disorders identification test
38D4.	Alcohol use disorder identification test consumption questionnaire
38D5.	Alcohol use disorder identification test Piccinelli consumption questionnaire
38Df.	Five-shot questionnaire on heavy drinking
38Dz.	Severity of alcohol dependence questionnaire
38P03	Health of the Nation Outcome Scale for Children and Adolescents item 4 - alcohol, substance/solvent misuse
38QA.	CIWA-Ar - Clinical Institute Withdrawal Assessment for Alcohol scale, revised
38QE.	Addiction Research Foundation Clinical Institute Withdrawal Assessment for Alcohol
44X3.	Blood ethanol level
66e..	Alcohol disorder monitoring
66e0.	Alcohol abuse monitoring
6792.	Health ed. - alcohol
67A5.	Pregnancy alcohol advice
67H0.	Lifestyle advice regarding alcohol
67K6.	Cycle of change stage, alcohol
6892.	Alcohol consumption screen
68S..	Alcohol consumption screen
7P221	Delivery of rehabilitation for alcohol addiction
8BA8.	Alcohol detoxification
8BAs.	Alcohol relapse prevention
8BAu.	Alcohol harm reduction programme
8BAw.	Alcohol twelve step programme
8CAM.	Patient advised about alcohol
8CAM0	Advised to abstain from alcohol consumption
8CAv.	Advised to contact primary care alcohol worker
8CE1.	Alcohol leaflet given
8CdK.	Specialist alcohol treatment service signposted
8G32.	Aversion therapy - alcoholism
8H35.	Admitted to alcohol detoxification centre
8H7p.	Referral to community alcohol team
8HHe.	Referral to community drug and alcohol team
8HkG.	Referral to specialist alcohol treatment service
8HkJ.	Referral to alcohol brief intervention service
8IA7.	Alcohol consumption screening test declined
8IAF.	Brief intervention for excessive alcohol consumption declined
8IAJ.	Declined referral to specialist alcohol treatment service
8IAt.	Extended intervention for excessive alcohol consumption declined
8IEA.	Referral to community alcohol team declined
8IH4.	Alcohol Use Disorders Identification Test declined
8W2..	Referral to mental health services deferred until alcohol misuse resolved

9EQ..	HO/RTS-police:venesect alc
9EVD.	Hospital alcohol liaison team report received
9NJz.	In-house alcohol detoxification
9NN2.	Under care of community alcohol team
9NgzH	Withdrawn from alcohol detoxification programme
9Nz9.	Emrgcy dept attn alcohl consum
9NzA.	Hospital attendance related to personal alcohol consumption
9k1..	Alcohol misuse - enhanced services administration
9k10.	Comm detoxification registered
9k11.	Alcohol consumption counselling
9k12.	Alcohol misuse - enhanced service completed
9k13.	Alcohol questionnaire completed
9k14.	Alcohol counselling by other agencies
9k15.	Alcohol screen - alcohol use disorder identification test completed
9k16.	Alcohol screen - fast alcohol screening test completed
9k17.	Alcohol screen - alcohol use disorder identification test consumption questions completed
9k18.	Alcohol screen - alcohol use disorder identification test Piccinelli consumption questions completed
9k19.	Alcohol assessment declined - enhanced services administration
9k1A.	Brief intervention for excessive alcohol consumption completed
9k1B.	Extended intervention for excessive alcohol consumption completed
C1505	Alcohol-induced pseudo-Cushing's syndrome
C253.	Wernickes encephalopathy
E01..	Alcoholic psychoses
E010.	Alcohol withdrawal delirium
E011.	Alcohol amnestic syndrome
E0110	Korsakov's alcoholic psychosis
E0111	Korsakov's alcoholic psychosis with peripheral neuritis
E0112	Wernicke-Korsakov syndrome
E011z	Alcohol amnestic syndrome NOS
E012.	Other alcoholic dementia
E0120	Chronic alcoholic brain syndrome
E013.	Alcohol withdrawal hallucinosis
E014.	Pathological alcohol intoxication
E015.	Alcoholic paranoia
E01y.	Other alcoholic psychosis
E01y0	Alcohol withdrawal syndrome
E01yz	Other alcoholic psychosis NOS
E01z.	Alcoholic psychosis NOS
E23..	Alcohol dependence syndrome
E230.	Acute alcoholic intoxication in alcoholism
E2300	Acute alcoholic intoxication, unspecified, in alcoholism
E2301	Continuous acute alcoholic intoxication in alcoholism
E2302	Episodic acute alcoholic intoxication in alcoholism

E2303	Acute alcoholic intoxication in remission, in alcoholism
E230z	Acute alcoholic intoxication in alcoholism NOS
E231.	Chronic alcoholism
E2310	Unspecified chronic alcoholism
E2311	Continuous chronic alcoholism
E2312	Episodic chronic alcoholism
E2313	Chronic alcoholism in remission
E231z	Chronic alcoholism NOS
E23z.	Alcohol dependence syndrome NOS
E250.	Nondependent alcohol abuse
E2500	Nondependent alcohol abuse, unspecified
E2501	Nondependent alcohol abuse, continuous
E2502	Nondependent alcohol abuse, episodic
E2503	Nondependent alcohol abuse in remission
E250z	Nondependent alcohol abuse NOS
Eu10.	[X]Mental and behavioural disorders due to use of alcohol
Eu100	[X]Mental and behavioural disorders due to use of alcohol: acute intoxication
Eu101	[X]Mental and behavioural disorders due to use of alcohol: harmful use
Eu102	[X]Mental and behavioural disorders due to use of alcohol: dependence syndrome
Eu103	[X]Mental and behavioural disorders due to use of alcohol: withdrawal state
Eu104	[X]Mental and behavioural disorders due to use of alcohol: withdrawal state with delirium
Eu105	[X]Mental and behavioural disorders due to use of alcohol: psychotic disorder
Eu106	[X]Mental and behavioural disorders due to use of alcohol: amnesic syndrome
Eu107	[X]Mental and behavioural disorders due to use of alcohol: residual and late-onset psychotic disorder
Eu108	[X]Alcohol withdrawal-induced seizure
Eu10y	[X]Mental and behavioural disorders due to use of alcohol: other mental and behavioural disorders
Eu10z	[X]Mental and behavioural disorders due to use of alcohol: unspecified mental and behavioural disorder
F11x0	Cerebral degeneration due to alcoholism
F1440	Cerebellar ataxia due to alcoholism
F25B.	Alcohol-induced epilepsy
F375.	Alcoholic polyneuropathy
F3941	Alcoholic myopathy
G555.	Alcoholic cardiomyopathy
G8523	Oesophageal varices in alcoholic cirrhosis of the liver
J153.	Alcoholic gastritis
J610.	Alcoholic fatty liver
J611.	Acute alcoholic hepatitis
J612.	Alcoholic cirrhosis of liver
J6120	Alcoholic fibrosis and sclerosis of liver
J613.	Alcoholic liver damage unspecified
J6130	Alcoholic hepatic failure

J617.	Alcoholic hepatitis
J6170	Chronic alcoholic hepatitis
J6708	Alcohol-induced acute pancreatitis
J6710	Alcohol-induced chronic pancreatitis
L2553	Maternal care for (suspected) damage to fetus from alcohol
PK80.	Fetal alcohol syndrome
PK83.	Fetus and newborn affected by maternal use of alcohol
Q0071	Fetus or neonate affected by placental or breast transfer of alcohol
R103.	[D]Alcohol blood level excessive
SLH3.	Alcohol deterrent poisoning
SM0..	Alcohol causing toxic effect
SM00.	Ethyl alcohol causing toxic effect
SM000	Ethanol causing toxic effect
SM001	Denatured alcohol-toxic effect
SM002	Grain alcohol causing toxic effect
SM00z	Ethyl alcohol causing toxic effect NOS
SM01.	Methyl alcohol - toxic effect
SM010	Methanol - toxic effect
SM011	Wood alcohol - toxic effect
SM01z	Methyl alcohol-toxic eff.NOS
SM02.	Isopropyl alcohol-toxic effect
SM020	Dimethyl carbinol-toxic effect
SM021	Isopropanol - toxic effect
SM022	Rubbing alcohol - toxic effect
SM02z	Isopropyl alcohol-tox.eff.NOS
SM03.	Fusel oil - toxic effect
SM030	Amyl alcohol - toxic effect
SM031	Butyl alcohol - toxic effect
SM032	Propyl alcohol - toxic effect
SM03z	Fusel oil - toxic effect NOS
SM0y.	Other alcohol - toxic effect
SM0z.	Alcohol causing toxic effect NOS
SyuG0	[X]Toxic eff of oth alcohols
T90..	Accidental poisoning by alcohol, NEC
T900.	Accidental poisoning by alcoholic beverages
T901.	Accidental poisoning by other ethyl alcohol and its products
T9010	Accid.pois.- denatured alcohol
T9011	Accid.pois.- methylated spirit
T9012	Accidental poisoning by grain alcohol NOS
T901z	Accidental poisoning by ethyl alcohol NOS
T902.	Accid.pois.- methyl alcohol
T903.	Accid.pois.- isopropyl alcohol
T9032	Accid.pois.- rubbing alc.subst
T904.	Accid.pois.- fusel oil

T90z.	Accidental poisoning by alcohol NOS
TJH3.	Adverse reaction to alcohol deterrents
U1A9.	[X]Accidental poisoning by and exposure to alcohol
U1A90	[X]Accidental poisoning by and exposure to alcohol, occurrence at home
U1A91	[X]Accidental poisoning by and exposure to alcohol, occurrence in residential institution
U1A92	[X]Accidental poisoning by and exposure to alcohol, occurrence at school, other institution and public administrative area
U1A93	[X]Accidental poisoning by and exposure to alcohol, occurrence at sports and athletics area
U1A94	[X]Accidental poisoning by and exposure to alcohol, occurrence on street and highway
U1A95	[X]Accidental poisoning by and exposure to alcohol, occurrence at trade and service area
U1A96	[X]Accidental poisoning by and exposure to alcohol, occurrence at industrial and construction area
U1A97	[X]Accidental poisoning by and exposure to alcohol, occurrence on farm
U1A9y	[X]Accidental poisoning by and exposure to alcohol, occurrence at other specified place
U1A9z	[X]Accidental poisoning by and exposure to alcohol, occurrence at unspecified place
U209.	[X]Intentional self poisoning by and exposure to alcohol
U2090	[X]Intentional self poisoning by and exposure to alcohol, occurrence at home
U2091	[X]Intentional self poisoning by and exposure to alcohol, occurrence in residential institution
U2092	[X]Intentional self poisoning by and exposure to alcohol, occurrence at school, other institution and public administrative area
U2093	[X]Intentional self poisoning by and exposure to alcohol, occurrence at sports and athletics area
U2094	[X]Intentional self poisoning by and exposure to alcohol, occurrence on street and highway
U2095	[X]Intentional self poisoning by and exposure to alcohol, occurrence at trade and service area
U2096	[X]Intentional self poisoning by and exposure to alcohol, occurrence at industrial and construction area
U2097	[X]Intentional self poisoning by and exposure to alcohol, occurrence on farm
U209y	[X]Intentional self poisoning by and exposure to alcohol, occurrence at other specified place
U209z	[X]Intentional self poisoning by and exposure to alcohol, occurrence at unspecified place
U409.	[X]Poisoning ?intent alcohol
U4090	[X]Poison ?intent alcohol home
U4091	[X]Pois ?int alcohol res ins
U4092	[X]Pois ?int alcohol pub inst
U4093	[X]Pois ?int alcohol sport ar
U4094	[X]Pois ?int alcohol on hway
U4095	[X]Pois ?int alcohol trade ar
U4096	[X]Pois ?int alcohol indust ar

U4097	[X]Poisoning by and exposure to alcohol, occurrence on farm, undetermined intent
U409y	[X]Pois ?int alcohol OS place
U409z	[X]Pois ?int alcohol unsp plc
U60H3	[X]Alcohol deterrents causing adverse effects in therapeutic use
U8...	[X]Supplementary factors related to causes of morbidity and mortality classified elsewhere
U80..	[X]Evidence of alcohol involvement determined by blood alcohol level
U800.	[X]Evidence of alcohol involvement determined by blood alcohol level of less than 20 mg/100 ml
U801.	[X]Evidence of alcohol involvement determined by blood alcohol level of 20-39 mg/100 ml
U802.	[X]Evidence of alcohol involvement determined by blood alcohol level of 40-59 mg/100 ml
U803.	[X]Evidence of alcohol involvement determined by blood alcohol level of 60-79 mg/100 ml
U804.	[X]Evidence of alcohol involvement determined by blood alcohol level of 80-99 mg/100 ml
U805.	[X]Evidence of alcohol involvement determined by blood alcohol level of 100-119 mg/100 ml
U806.	[X]Evidence of alcohol involvement determined by blood alcohol level of 120-199 mg/100 ml
U807.	[X]Evidence of alcohol involvement determined by blood alcohol level of 200-239 mg/100 ml
U808.	[X]Evidence of alcohol involvement determined by blood alcohol level of 240 mg/100 ml or more
U80z.	[X]Evidence of alcohol involvement determined by presence of alcohol in blood, level not specified
U81..	[X]Evidence of alcohol involvement determined by level of intoxication
U810.	[X]Evidence of alcohol involvement determined by level of intoxication, mild alcohol intoxication
U811.	[X]Evidence of alcohol involvement determined by level of intoxication, moderate alcohol intoxication
U812.	[X]Evidence of alcohol involvement determined by level of intoxication, severe alcohol intoxication
U813.	[X]Evidence of alcohol involvement determined by level of intoxication, very severe alcohol intoxication
U814.	[X]Evidence of alcohol involvement determined by level of intoxication, alcohol involvement, not otherwise specified
ZV113	[V]Personal history of alcoholism
ZV4KC	[V] Alcohol use
ZV57A	[V]Alcohol rehabilitation
ZV6D6	[V]Alcohol abuse counselling and surveillance
ZV704	[V]Medicolegal examination
ZV70L	[V]Blood-alcohol and blood-drug test
ZV791	[V]Screening for alcoholism
du1..	DISULFIRAM
du11.	DISULFIRAM 200mg tablets
du12.	ANTABUSE 200mg tablets

du5..	ACAMPROSATE CALCIUM
du51.	ACAMPROSATE CAL 333mg e/c tabs
du52.	CAMPRAL EC 333mg e/c tablets
