# Supplementary material for: How does the local area deprivation influence life chances for children in poverty in Wales: A record linkage cohort study
Source: SSM Popul Health. 2023 Feb 23;22:101370. doi: 10.1016/j.ssmph.2023.101370 (PMC9986621; doi:10.1016/j.ssmph.2023.101370)
Supplement: Multimedia component 6 [file mmc6.pdf]

> omcdiag(mod1.1, Inter = TRUE)

Call:
omcdiag(mod = mod1.1, Inter = TRUE)

Overall Multicollinearity Diagnostics

	MC Results	detection
Determinant |X'X|:	0.0279	0
Farrar Chi-Square:	569247.6107	1
Red Indicator:	0.1892	0
Sum of Lambda Inverse:	26.7808	0
Theil's Method:	0.6360	1
Condition Number:	4099.8150	1

1 --> COLLINEARITY is detected by the test
0 --> COLLINEARITY is not detected by the test

> imcdiag(mod1.1)

All Individual Multicollinearity Diagnostics Result

	VIF	TOL	Wi	Fi	Leamer	CVIF	Klein	IND1	IND2
INC_WIMD	4.8513	0.2061	40852.8535	43771.1896	0.4540	6.8168	1	0e+00	3.4200
HEA_WIMD	2.8898	0.3460	20046.0999	21478.0991	0.5883	4.0606	1	0e+00	2.8173
ACC_WIMD	1.6831	0.5942	7245.8284	7763.4363	0.7708	2.3650	1	1e-04	1.7484
COM_WIMD	3.9019	0.2563	30781.8968	32980.8109	0.5062	5.4828	1	0e+00	3.2039
PHYENV_WIMD	1.0603	0.9431	639.5007	685.1836	0.9712	1.4899	0	1e-04	0.2450
HOUS_WIMD	1.5472	0.6463	5804.9749	6219.6551	0.8039	2.1741	1	1e-04	1.5237
FSM	1.1727	0.8528	1831.6378	1962.4815	0.9234	1.6478	0	1e-04	0.6343
EXAM_YEAR	1.0066	0.9935	69.9074	74.9013	0.9967	1.4144	0	1e-04	0.0282
GNDR_CD	1.0186	0.9818	196.7749	210.8316	0.9909	1.4312	0	1e-04	0.0785
LIVING_AREA	1.3749	0.7273	3976.5289	4260.5934	0.8528	1.9319	1	1e-04	1.1746
ADULT_HHM	1.0364	0.9648	386.4502	414.0564	0.9823	1.4564	0	1e-04	0.1514
CHILD_HHM	1.0510	0.9515	540.9152	579.5556	0.9754	1.4768	0	1e-04	0.2090
HHM_ALCOHOL_HA	1.0366	0.9647	388.7128	416.4806	0.9822	1.4567	0	1e-04	0.1523
HHM_DEPRESSION	1.0662	0.9379	702.5241	752.7091	0.9684	1.4982	0	1e-04	0.2676
HHM_SMI	1.0224	0.9781	237.7611	254.7456	0.9890	1.4367	0	1e-04	0.0944
SEN	1.0619	0.9417	657.0684	704.0063	0.9704	1.4922	0	1e-04	0.2513

1 --> COLLINEARITY is detected by the test
0 --> COLLINEARITY is not detected by the test

PHYENV_WIMD , HHM_SMI , coefficient(s) are non-significant may be due to multicollinearity

R-square of y on all x: 0.2052

* use method argument to check which regressors may be the reason of collinearity

Discussion:

An overall collinearity test has been performed using ‘mctest’ packages in R. It provides the Farrar-Glauber test and other relevant tests for multicollinearity. An overall and individual level diagnostic check for multicollinearity have been performed using ‘omcdiag’ and ‘imcdiag’ function. The VIF column provides the diagnostic output for variance inflation factor. High multicollinearity can be assessed using the VIF statistic. According to collinearity guidelines in the National Survey it is generally suggested that individual variable VIF is no greater than 10 and the mean VIF is no greater than 2.5, suggesting that there is no cause for concern of collinearity in the models. The results clearly show that VIF no greater than 10, so the concern around the high degree of correlation between predictor variables in a regression model can be avoided in this case.
