# Supplementary material for: How does the local area deprivation influence life chances for children in poverty in Wales: A record linkage cohort study
Source: SSM Popul Health. 2023 Feb 23;22:101370. doi: 10.1016/j.ssmph.2023.101370 (PMC9986621; doi:10.1016/j.ssmph.2023.101370)
Supplement: Multimedia component 8 [file mmc8.pdf]

Table 1: Logistic regression models of the association between WIMD components and achieving each of the PLP components for the FSM and non-FSM children

	Achieving KS4	Not having mental health problem	Not having substance misuse and alcohol problem
FSM children			
WIMD	OR (Lower CI, Upper CI)		
1(Most deprived)	1	1	1
2	1.32 (1.22, 1.42)	0.88 (0.79, 0.97)	0.83 (0.73, 0.93)
3	1.52 (1.39, 1.66)	0.91 (0.81, 1.03)	0.82 (0.71, 0.94)
4	1.82 (1.62, 2.04)	0.86 (0.73, 1.01)	0.79 (0.66, 0.95)
5 (Least deprived)	2.53 (2.23, 2.88)	0.76 (0.64, 0.91)	0.83 (0.67, 1.03)
Non-FSM children:			
1(Most deprived)	1	1	1
2	1.42 (1.37, 1.47)	1.17 (1.08, 1.26)	0.98 (0.91, 1.06)
3	1.94 (1.87, 2.02)	1.23 (1.14, 1.33)	0.97 (0.89, 1.05)
4	2.50 (2.40, 2.60)	1.31 (1.21, 1.43)	1.03 (0.95, 1.13)
5 (Least deprived)	3.91 (3.76, 4.06)	1.30 (1.21, 1.41)	1.21 (1.12, 1.32)

*Models area adjusted for Exam Year, Gender, Living area, Number of adults in the household, Number of children in the household, Living with someone who had alcohol problem, Living with someone who had depression, Living with someone who had serious mental illness, Special Education Need
