# Supplementary material for: How does the local area deprivation influence life chances for children in poverty in Wales: A record linkage cohort study
Source: SSM Popul Health. 2023 Feb 23;22:101370. doi: 10.1016/j.ssmph.2023.101370 (PMC9986621; doi:10.1016/j.ssmph.2023.101370)
Supplement: Multimedia component 9 [file mmc9.pdf]

Table 2: Unadjusted Logistic regression model with interaction between WIMD and FSM and their association with PLP

Variables	OR	Lowe CI	Upper CI
FSM: WIMD1 (most deprived)	1		
FSM: WIMD2	1.28	1.18	1.38
FSM: WIMD3	1.49	1.36	1.63
FSM: WIMD4	1.79	1.60	2.00
FSM: WIMD5 (least deprived)	2.35	2.08	2.65
Non-FSM: WIMD1 (most deprived)	2.74	2.60	2.89
Non-FSM: WIMD2	1.14	1.05	1.24
Non-FSM: WIMD3	1.35	1.22	1.48
Non-FSM: WIMD4	1.42	1.27	1.60
Non-FSM: WIMD5 (least deprived)	1.67	1.47	1.90

**Intercept (0.22 (0.21 – 0.24)*
