# Supplementary material for: How does the local area deprivation influence life chances for children in poverty in Wales: A record linkage cohort study
Source: SSM Popul Health. 2023 Feb 23;22:101370. doi: 10.1016/j.ssmph.2023.101370 (PMC9986621; doi:10.1016/j.ssmph.2023.101370)
Supplement: Multimedia component 10 [file mmc10.pdf]

Table 3: Adjusted Logistic regression model with interaction between WIMD and FSM and their association with PLP

Variables	OR	Lowe CI	Upper CI
FSM: WIMD1 (most deprived)	1		
FSM: WIMD2	1.26	1.16	1.37
FSM: WIMD3	1.42	1.29	1.56
FSM: WIMD4	1.74	1.54	1.96
FSM: WIMD5 (least deprived)	2.19	1.92	2.50
Non-FSM: WIMD1 (most deprived)	2.09	1.98	2.22
Non-FSM: WIMD2	1.11	1.02	1.22
Non-FSM: WIMD3	1.33	1.21	1.47
Non-FSM: WIMD4	1.36	1.20	1.54
Non-FSM: WIMD5 (least deprived)	1.60	1.40	1.84

**Intercept (0.27 (0.25 – 0.28)*

**Adjusted by exam year, living area, Number of children in the household, living with someone who had alcohol problem, living with someone who had depression, Special Education Need*
